# Supplementary material for: The circadian cryptochrome, CRY1, is a pro-tumorigenic factor that rhythmically modulates DNA repair
Source: Nat Commun. 2021 Jan 15;12:401. doi: 10.1038/s41467-020-20513-5 (PMC7810852; doi:10.1038/s41467-020-20513-5)
Supplement: Supplementary file 1 — Supplementary Information [file 41467_2020_20513_MOESM1_ESM.pdf]

Supplementary Figure 1

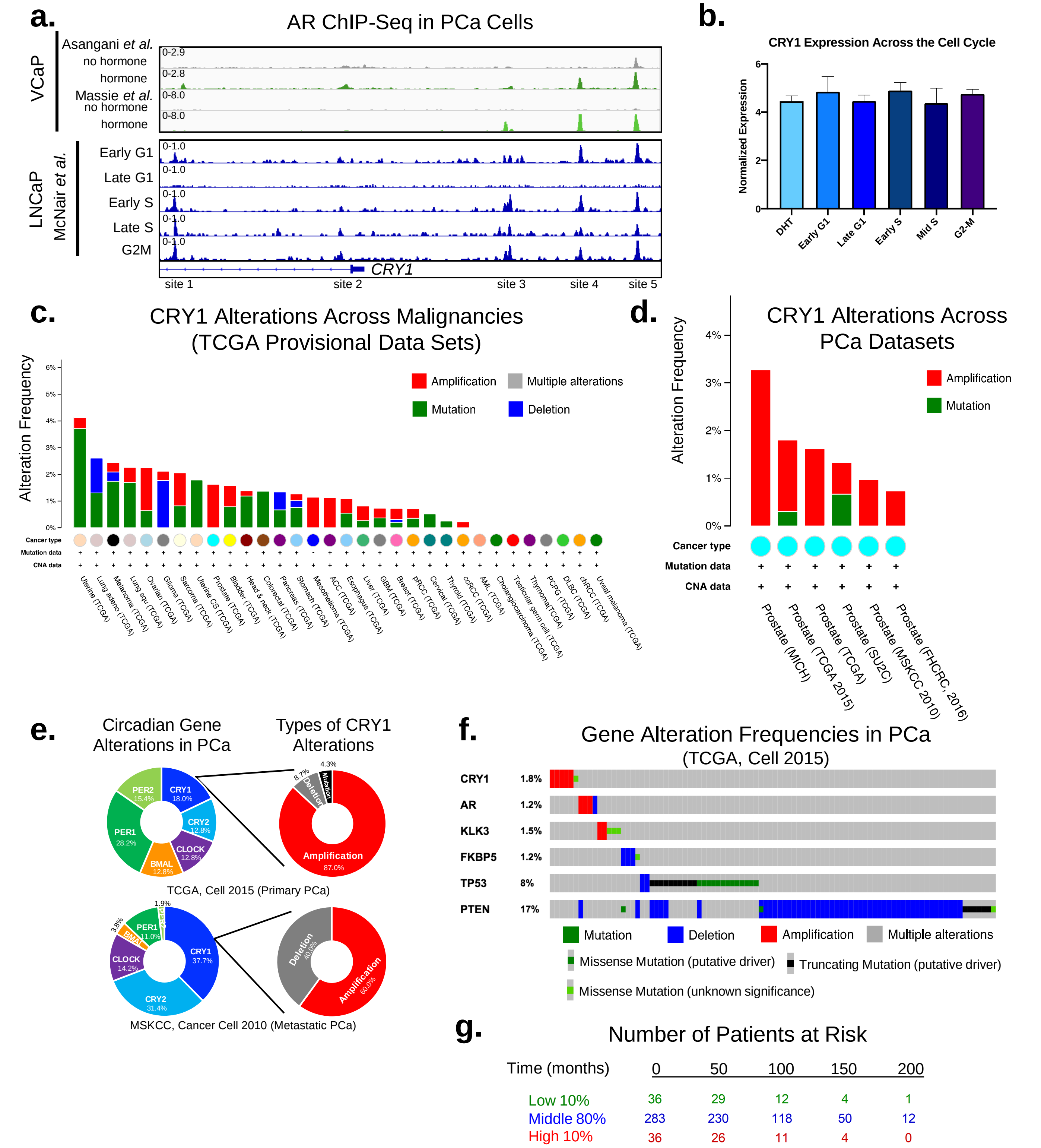

Supplementary Figure 1. Altered CRY1 expression is associated with different types of cancer. **A.** AR binding sites on CRY1 in PCa data sets of VCaP cells (Asangani *et al.* 2014 and Massie *et al.* 2011) and LNCaP cells (McNair *et al.* 2017, in early G1, late G1, early S, late S, and G2M phase). Genomic traces showing AR binding sites on CRY1. **B.** CRY1 mRNA expression across the phases of the cell cycle. **C-D.** Frequency of CRY1 alterations (i.e. amplifications, mutations, and/or deletions) in different cancer types (C) and in primary and/or metastatic PCa datasets (D) from cBioPortal. **E.** Frequency of core circadian clock gene alterations (i.e. amplifications, mutations, and/or deletions) in primary and metastatic PCa datasets from cBioPortal. **F.** Frequency of CRY1, AR, KLK3, FKBP5, TP53, and PTEN alterations in primary PCa. **G.** Number of patients at risk in each cohort (low, middle, and high) in the JHMI retrospective cohort for poor metastatic PCa outcome.

Supplementary Figure 2

a.

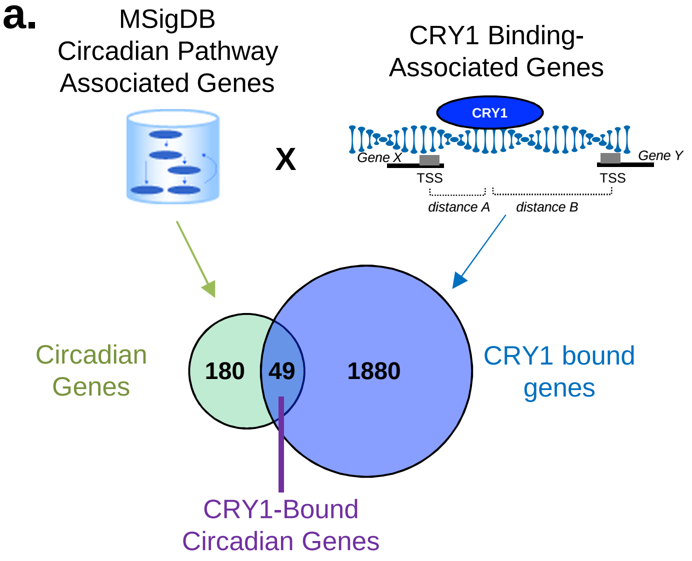

b.

Gene Sets Used for Circadian Clock Regulation
BIOCARTA CIRCADIAN PATHWAY
GO CIRCADIAN REGULATION OF GENE EXPRESSION
GO CIRCADIAN RHYTHM
GO CIRCADIAN SLEEP WAKE CYCLE
GO CIRCADIAN SLEEP WAKE CYCLE NON REM SLEEP
GO CIRCADIAN SLEEP WAKE CYCLE REM SLEEP
GO CIRCADIAN SLEEP WAKE CYCLE SLEEP
GO ENTRAINMENT OF CIRCADIAN CLOCK
GO ENTRAINMENT OF CIRCADIAN CLOCK BY PHOTOPERIOD
GO NEGATIVE REGULATION OF CIRCADIAN RHYTHM
GO NEGATIVE REGULATION OF CIRCADIAN SLEEP WAKE CYCLE SLEEP
GO POSITIVE REGULATION OF CIRCADIAN RHYTHM
GO POSITIVE REGULATION OF CIRCADIAN SLEEP WAKE CYCLE SLEEP
GO REGULATION OF CIRCADIAN RHYTHM
GO REGULATION OF CIRCADIAN SLEEP WAKE CYCLE
KEGG CIRCADIAN RHYTHM MAMMAL
PID CIRCADIAN PATHWAY
REACTOME BMAL1:CLOCK NPAS2 ACTIVATES CIRCADIAN GENE EXPRESSION
REACTOME CIRCADIAN CLOCK

c.

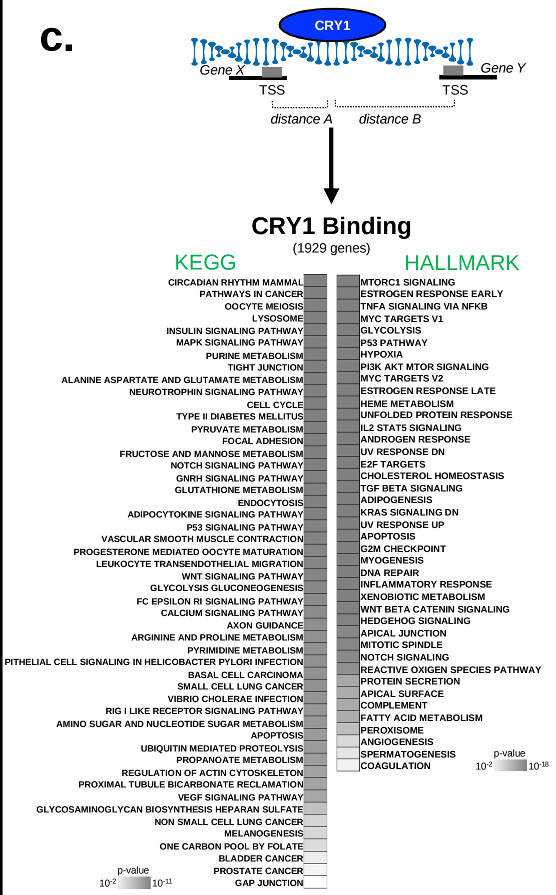

d.

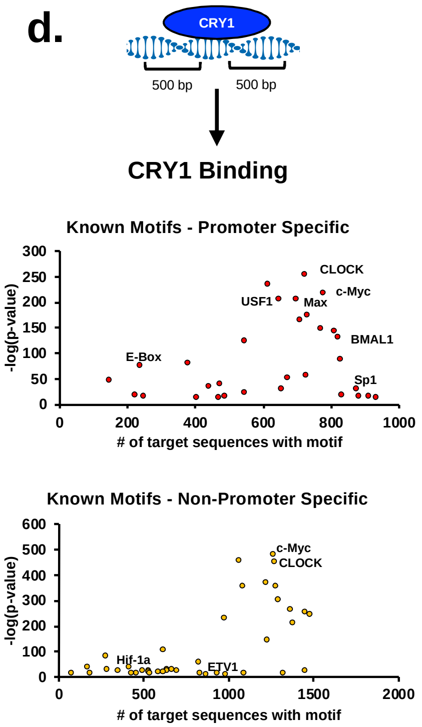

e.

List of Known Motifs of (Promoter Specific)

bHLHE40	KLF10	NFY
bHLHE41	KLF14	NPAS
BMAL1	KLF3	NPAS2
CLOCK	Klf4	Sp1
c-Myc	KLF5	Sp2
c-Myc	KLF6	Sp5
CRE	Klf9	TFE3
E-box	Max	USF1
Elk1	MITF	Usf2
HIF-1a	MNT	
HIF-1b	n-Myc	

List of Known Motifs of (Non-Promoter Specific)

bHLHE40	ETV4	Foxo3	NF1:FOXA1
bHLHE41	Fox:Ebox	FOXP1	NPAS
BMAL1	FOXA1	HEB	NPAS2
CLOCK	FOXA1	HIF-1a	Ptf1a
c-Myc	Foxa2	HIF-1b	Sp1
c-Myc	Foxa3	Max	TFE3
CTCF	Foxf1	MITF	USF1
E-box	FOXK1	MNT	Usf2
EHF	FOXK2	n-Myc	
ERG	FoxL2	NF1	
ETV1	FOXM1	NF1-halfsite	

Supplementary Figure 2. The CRY1 cistrome is enriched for cancer-associated pathways. A. Schematic describing the comparison of ChIP-Seq data with known circadian factors. Briefly, the CRY1-bound circadian genes were identified by comparing all the circadian genes (curated from the MSigDB of circadian pathways) to the genes with a CRY1 binding site within a TSS (transcriptional start site) of binding. **B.** List of all the MSigDB Gene Sets used for curating the list of circadian genes used in the comparison. **C.** KEGG and Hallmark Pathway analysis of genes bound by CRY1 within a transcriptional start site (TSS) of a gene with CRY1 binding with an FDR<0.25. **D.** Known motif enrichments for promoter specific and non-promoter specific motifs of CRY1 binding in vehicle treated C4-2 cells within 500 bp binding window on each side of the center of binding. **E.** List of known motifs factors for promoter specific and non-promoter specific regions.

Supplementary Figure 3

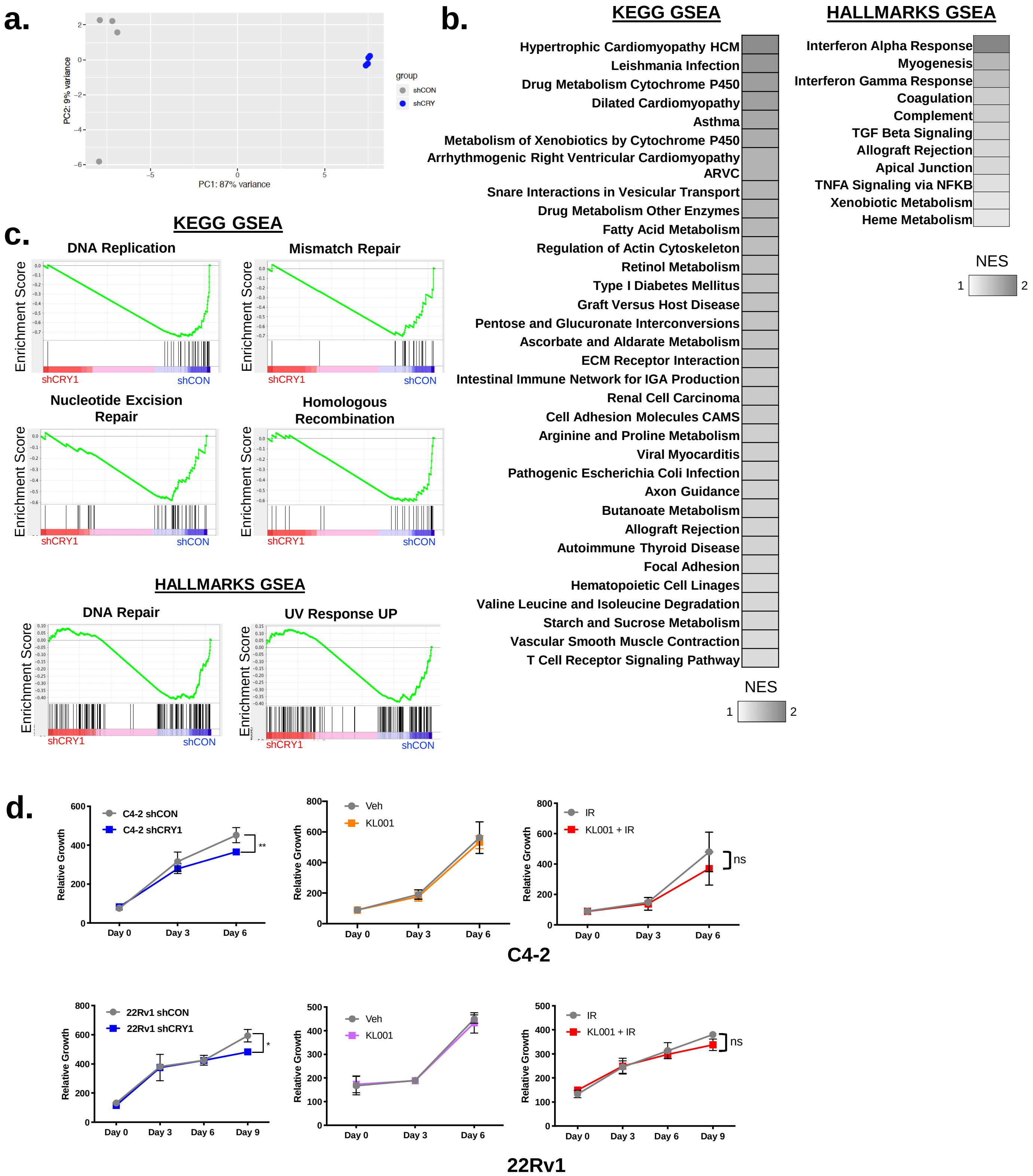

Supplementary Figure 3. Genome-wide assessment of CRY1 transcriptome identifies several DNA repair processes. A. Principal component analysis (PCA) plots of the RNA-Seq samples run. **B.** GSEA of RNA-Seq (KEGG and HALLMARKS Pathway Analysis from MSigDB) was used to identify enriched and deenriched pathways for CRY1-regulated pathways using FDR<0.25. **C.** Leading-edge plots of the DNA repair pathways identified from the GSEA of the RNA-Seq data (KEGG and Hallmarks Pathway Analysis). **D.** CRY1 expression was knocked down in C4-2-shCRY1 and 22Rv1-shCRY1 cells (in hormone-proficient media) for indicated days. C4-2 and 22Rv1 cells were treated with 10 µM KL001 at Day 0 and harvested at days 0, 3, and 6 for Pico Green to assess relative growth. Cells were counted for each time point and graphed as relative growth. N=3 independent experiments. Data are presented as mean values +/− SEM and analyzed using one-way Anova (*p<0.05, **p<0.01, ***p<0.001, &, ****p<0.0001). Statistical significance was evaluated at 0.05 alpha level with GraphPadPrism, version 8.3.1, Mac. Source data are provided in the Source Data file.

Supplementary Figure 4

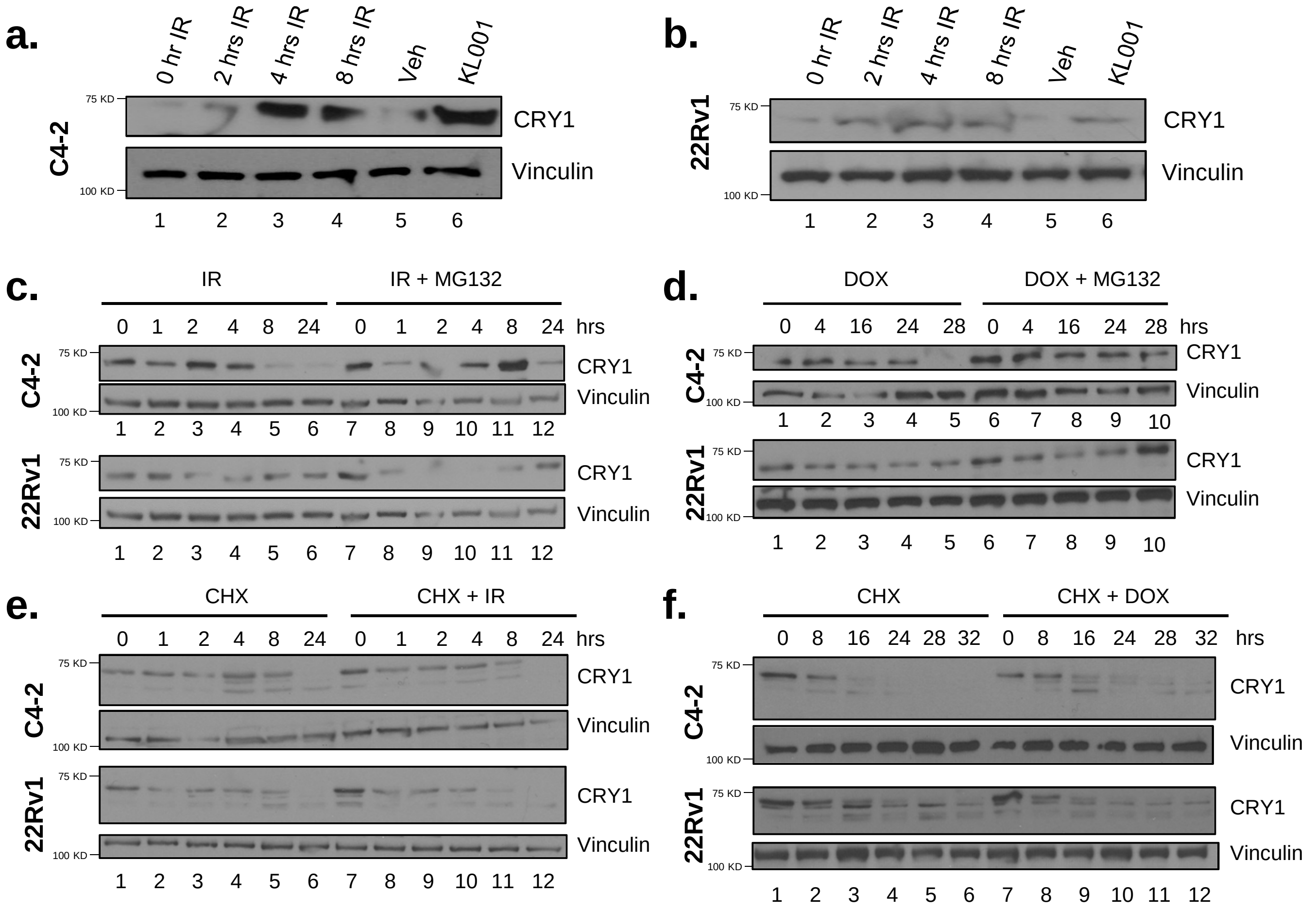

Supplementary Figure 4. DNA damage stabilizes CRY1 protein expression. A-B. C4-2 and 22Rv1 cells were treated 5 Gy IR for 0-8 hours and with 10 µM KL001 for 6 and 24 hours, respectively. **C.** C4-2 and 22Rv1 cells were treated 1 µM MG132 and 2 or 5 Gy IR for 1, 2, 4, 8, and 24 hrs. **D.** C4-2 and 22Rv1 cells were treated 1 µM MG132 and 10 nM DOX for 8, 16, 24, 28, and 32 hrs. **E.** C4-2 and 22Rv1 cells were treated 10 µg/mL CHX and 2 or 5 Gy IR for 1, 2, 4, 8, and 24 hrs. **F.** C4-2 and 22Rv1 cells were treated 10 µg/mL CHX and 10 nM DOX for 8, 16, 24, 28, and 32 hrs. **A-F.** Cells were harvested and protein expression of CRY1 and Vinculin was analyzed. N=3 independent experiments.

Supplementary Figure 5

a.

Gene Sets Used for Each Type of DDR
**Homologous Recombination (HR)**
GO REGULATION OF DOUBLE STRAND BREAK REPAIR VIA HOMOLOGOUS RECOMBINATION
KEGG HOMOLOGOUS RECOMBINATION
REACTOME HOMOLOGOUS RECOMBINATION REPAIR OF REPLICATION INDEPENDENT DOUBLE STRAND BREAKS
**Mismatch Repair (MMR)**
GO MISMATCH REPAIR
GO MISMATCH REPAIR COMPLEX
GO MISMATCHED DNA BINDING
KEGG MISMATCH REPAIR
**Nucleotide Excision Repair (NER)**
GO GLOBAL GENOME NUCLEOTIDE EXCISION REPAIR
GO NUCLEOTIDE EXCISION REPAIR
GO NUCLEOTIDE EXCISION REPAIR COMPLEX
GO NUCLEOTIDE EXCISION REPAIR DNA DAMAGE RECOGNITION
GO NUCLEOTIDE EXCISION REPAIR DNA DUPLEX UNWINDING
GO NUCLEOTIDE EXCISION REPAIR DNA GAP FILLING
GO NUCLEOTIDE EXCISION REPAIR DNA INCISION
GO NUCLEOTIDE EXCISION REPAIR PREINCISION COMPLEX ASSEMBLY
GO NUCLEOTIDE EXCISION REPAIR PREINCISION COMPLEX STABILIZATION
GO TRANSCRIPTION COUPLED NUCLEOTIDE EXCISION REPAIR
KEGG NUCLEOTIDE EXCISION REPAIR
NUCLEOTIDE EXCISION REPAIR
**Base Excision Repair (BER)**
BASE EXCISION REPAIR
GO BASE EXCISION REPAIR
KEGG BASE EXCISION REPAIR
REACTOME BASE EXCISION REPAIR
REACTOME NUCLEOTIDE EXCISION REPAIR
**Non-Homologous End Joining (NHEJ)**
KEGG NON HOMOLOGOUS END JOINING

b.

Genes in Each List

HR	MMR	NER			BER		NHEJ
ATM	ABL1	AQR	NTHL1	SUMO3	APEX1	UNG	DCLRE1C
BLM	EXO1	ATXN3	OGG1	TCEA1	APEX2	USP47	DNTT
BRCA1	LIG1	BRCA2	PARP1	TP53	CCNO	WRN	FEN1
BRCA2	MLH1	CCNH	PCNA	UBA52	DNA2	XPA	LIG4
BRIP1	MLH3	CDK7	PNKP	UBB	ERCC6	XRCC1	LOC731751
C12orf48	MSH2	CETN2	POLB	UBC	FEN1		MRE11A
CHEK1	MSH3	CHD1L	POLD1	UBE2I	HMGA1		NHEJ1
EME1	MSH4	CHRAC1	POLD2	UBE2N	HMGA2		POLL
FIGNL1	MUTYH	COPS4	POLD3	UBE2V2	HMGB1P10		RAD50
FIGNL2	PCNA	COPS5	POLD4	USP45	HMGB1P40		XRCC4
H2AFX	PMS1	COPS6	POLE	USP7	HMGB2		XRCC5
HELB	PMS2	COPS7A	POLE2	XAB2	HUWE1		XRCC6
KDM1A	PMS2CL	COPS7B	POLE3	XPA	LIG1		
KIAA0146	PMS2P1	COPS8	POLE4	XPC	LIG3		
LIG1	PMS2P3	CUL4A	POLK	XPCCETN2	MBD4		
LOC651610	PMS2P5	CUL4B	POLL	XRCC1	MPG		
MDC1	POLD1	DDB1	POLR2A	ZNF830	MSH2		
MRE11A	POLD2	DDB2	POLR2B		MSH3		
MUS81	POLD3	EP300	POLR2C		MSH6		
NBN	POLD4	ERCC1	POLR2D		MUTYH		
POLD1	RFC1	ERCC2	POLR2E		NEIL1		
POLD2	RFC2	ERCC3	POLR2F		NEIL2		
POLD3	RFC3	ERCC4	POLR2G		NEIL3		
POLD4	RFC4	ERCC5	POLR2H		NTHL1		
POLQ	RFC5	ERCC6	POLR2I		OGG1		
PPP4C	RNASEH2A	ERCC8	POLR2J		PARP1		
PPP4R2	RPA1	FAN1	POLR2K		PARP2		
RAD50	RPA2	GPS1	POLR2L		PARP3		
RAD51	RPA3	GTF2H1	PPIE		PARP4		
RAD51AP1	RPA4	GTF2H2	PRPF19		PCNA		
RAD51B	SETD2	GTF2H2B	RAD23A		POLB		
RAD51C	SSBP1	GTF2H2C	RAD23B		POLD1		
RAD51D	TDG	GTF2H2D	RAD51D		POLD2		
RAD52	TP73	GTF2H3	RBBP8		POLD3		
RAD54B	TREX1	GTF2H4	RBX1		POLD4		
RAD54L	WRN	GTF2H5	RFC1		POLE		
RPA1	XPC	HMGN1	RFC2		POLE2		
RPA2		HUS1	RFC3		POLE3		
RPA3		HUS1B	RFC4		POLE4		
RPA4		ISY1	RFC5		POLG		
RTEL1		KIAA1530	RNF111		POLL		
SHFM1		LIG1	RPA1		POLQ		
SIRT6		LIG3	RPA2		PRMT6		
SSBP1		LIG4	RPA3		RECQL4		
TERF2IP		LOC652672	RPA4		RPA1		
TEX15		LOC652857	RPS27A		RPA2		
TOP3A		MMS19	SIRT1		RPA3		
TOP3B		MNAT1	SLC30A9		SIRT6		
TP53BP1		NEIL1	SLX4		SMUG1		
XRCC2		NEIL2	SUMO1		TDG		
XRCC3		NEIL3	SUMO2		TP53		

c.

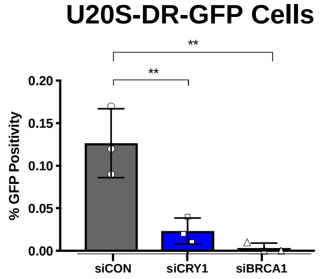

Supplementary Figure 5. Genome-wide assessment of CRY1 identifies direct regulation of DNA repair processes. A. List of all the MSigDB Gene Sets used for all the different types of DDR – HR, MMR, NER, BER, and NHEJ used in the comparison of RNA-Seq and ChIP-Seq. **B.** List of all the DDR genes regulated by CRY1 in each specific type of DDR pathway. **C.** CRY1 expression was knocked down in U20S-DR-GFP cells for 72 hrs via siRNA or cells were treated siBRCA1 for 24 hrs and then transfected with I-Sce1 to induce double-strand breaks. Cells were harvested for flow cytometry to analyze GFP positive cells. N=3 independent experiments. Data are presented as mean values +/− SEM and analyzed using one-way Anova (**p<0.01). Statistical significance was evaluated at 0.05 alpha level with GraphPadPrism, version 8.3.1, Mac. Source data are provided in the Source Data file.

Supplementary Figure 6

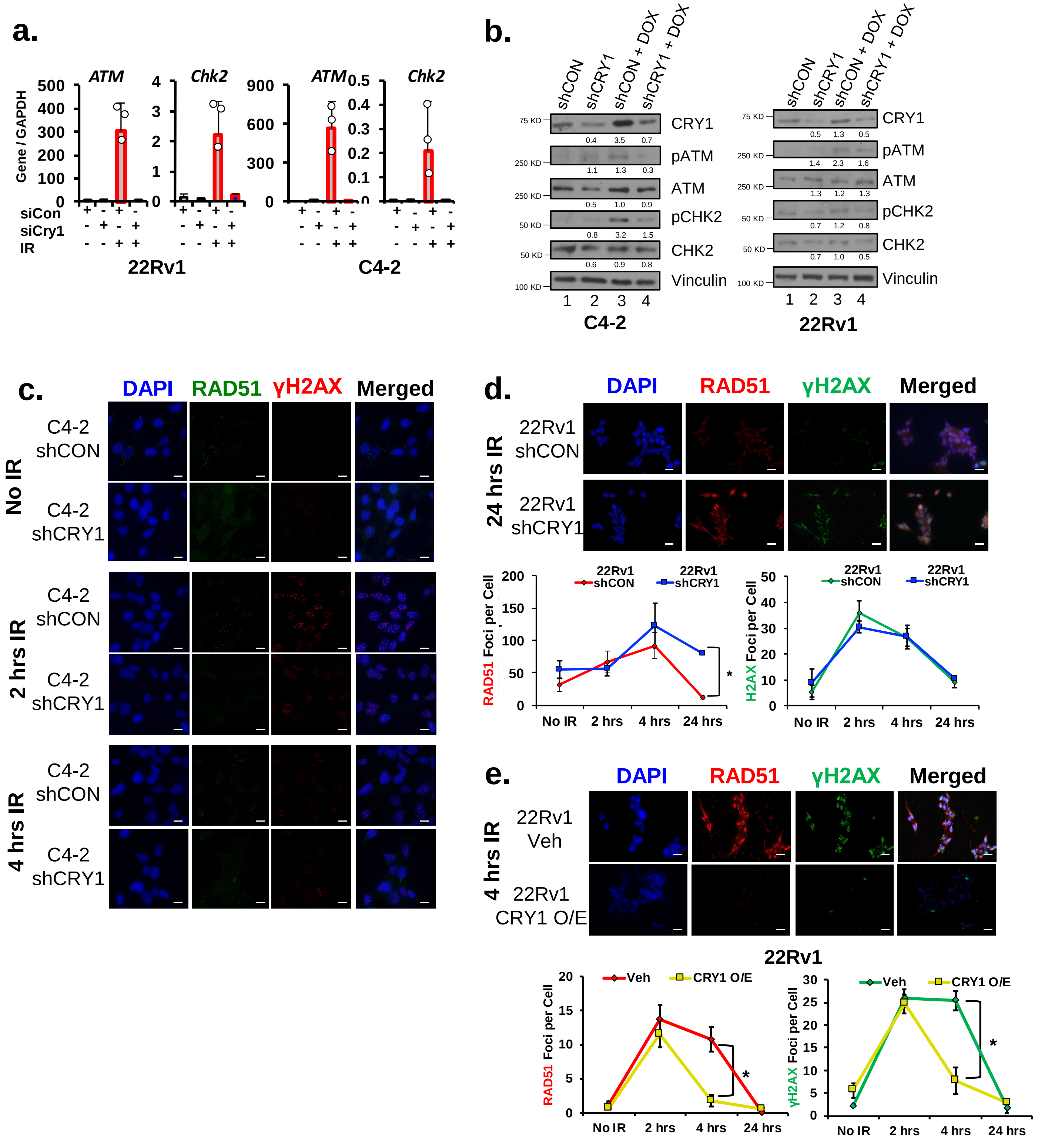

Supplementary Figure 6. CRY1 promotes homologous recombination (HR)-mediated DNA damage response. A. C4-2 and 22Rv1 cells were treated with siCON and siCRY1 for 72 hours, treated with IR, and harvested for RNA. **B-D.** CRY1 expression was knocked down in C4-2-shCRY1 and 22Rv1-shCRY1 cells for 72 hrs. **B.** Cells were treated with 10 µM DOX for 24 hrs. Cells were harvested for protein. Expression of CRY1, pATM, ATM, pCHK2, CHK2, and Vinculin protein were analyzed. **C-D.** Cells were treated with 5 Gy for 2, 4, or 24 hrs. Cells were fixed at the indicated time points, stained with γ-H2AX and RAD51 antibodies, and imaged by confocal microscopy. Scale bar 250 µm. **E.** CRY1 expression was transiently overexpressed with transfection of a CRY1 plasmid for 48 hrs and then treated with 5 Gy IR for 2, 4, and 24 hrs in 22Rv1 cells. Cell were stained with γ-H2AX & RAD51 antibodies and imaged by confocal microscopy. Scale bar 250 µm. N=3 independent experiments. Data are presented as mean values +/− SEM and analyzed using one-way Anova (*p<0.05). Statistical significance was evaluated at 0.05 alpha level with GraphPadPrism, version 8.3.1, Mac. Source data are provided in the Source Data file.

Supplementary Figure 7

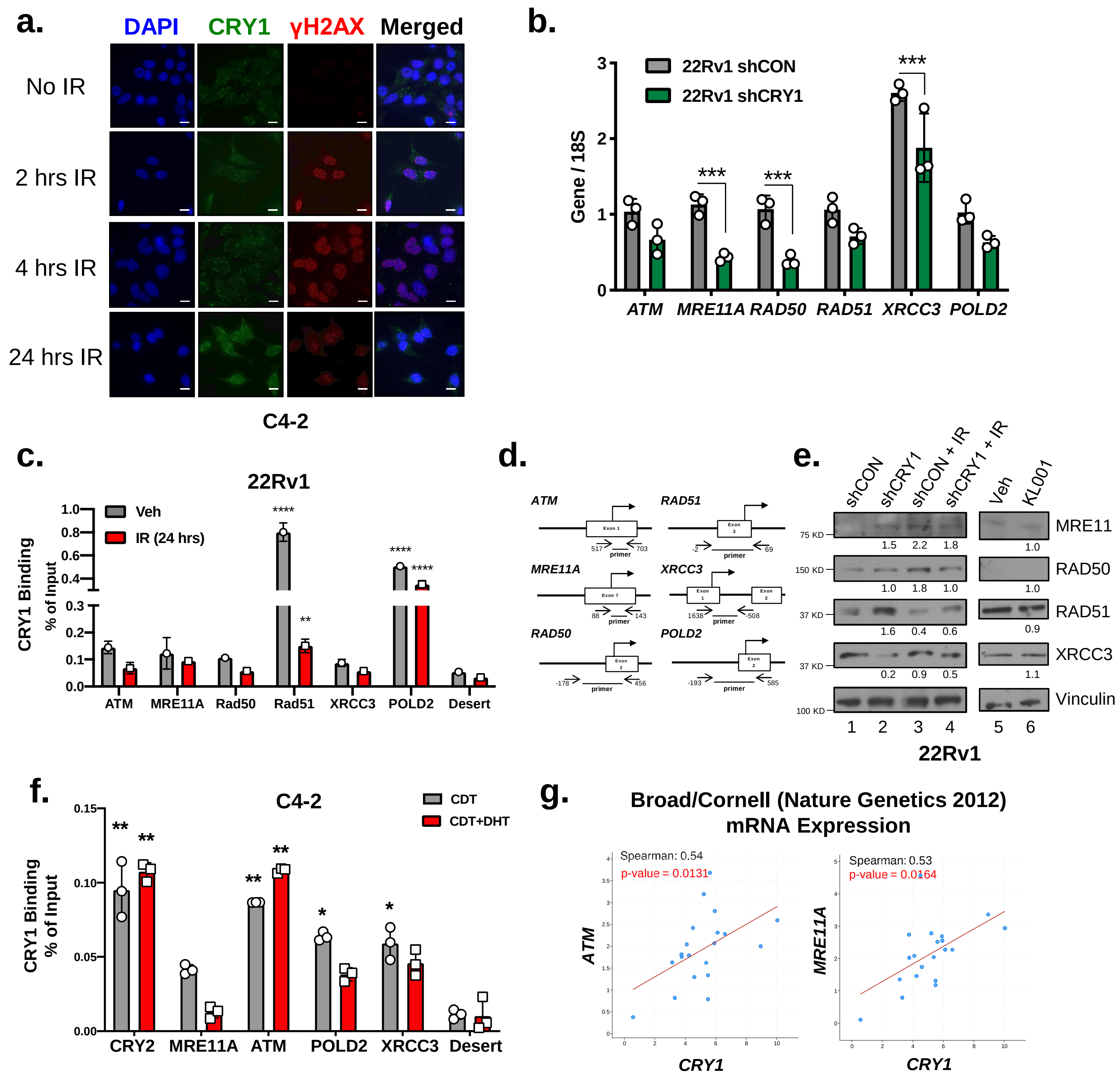

Supplementary Figure 7. CRY1 directly binds to promoters of HR genes and regulates HR gene expression to promote DNA repair. A. C4-2 cells were treated with 5 Gy IR for 2, 4, and 24 hrs. Cells were fixed at the indicated time points, stained with γ-H2AX and CRY1 antibodies, and imaged by confocal microscopy. Scale bar 250 μm. **B.** CRY1 expression was knocked down in 22Rv1-shCRY1 cells for 72 hrs. Cells were harvested for RNA. *ATM, Mre11A, Rad50, Rad51, XRCC3, POLD2,* and *18S* mRNA. **C.** CRY1 ChIP qPCR was performed on the promoter of ATM, Rad51, Mre11A, Rad50, XRCC3, POLD2, and a desert region to determine the binding of CRY1 after 24 hrs of 5 Gy IR treatment in 22Rv1 cells and in vehicle condition in 22Rv1 cells. Binding is plotted as percent input. **D.** Schematic of primers designed for each CRY1 ChIP site on the HR genes. Numbers indicate nucleotides from start of exon. **E.** CRY1 expression was knocked down in 22Rv1-shCRY1 cells for 72 hrs and then cells were treated with 5 Gy IR for 24 hrs. 22Rv1 cells were treated with 10 μM KL001 (CRY1 activator) for 24 hrs. Cells were harvested for protein. MRE11A, RAD50, RAD51, XRCC3, and vinculin were analyzed. **F.** CRY1 ChIP qPCR was performed on CRY2, Mre11A, ATM, XRCC3, POLD2, and a desert region to determine the binding of CRY1 in CDT and CDT+DHT (10 nM, 2 hours) treatment in C4-2 cells. Binding is plotted as percent input. **G.** Co-expression of CRY1 and either ATM, MRE11A, and RAD50 mRNA in PCa tissue from publicly available data from Broad/Cornell (Nature Genetics 2012). N=3 independent experiments. Data are presented as mean values +/− SEM and analyzed using one-way Anova (*p<0.05, **p<0.01, ***p<0.001, &, ****p<0.0001). Statistical significance was evaluated at 0.05 alpha level with GraphPadPrism, version 8.3.1, Mac. Source data are provided in the Source Data file.

Supplementary Table 1

ChIP qPCR Primer Sequences

	Sequence (5' to 3')	
Target	Forward	Reverse
CCND3	ACGGAGGCTCAGGTGTGG	GGATAGCACGTGGAGGAGTT
CCNE1	AATGCACAGGGGCTCTTAGG	GAGCACCTACGTACAGCCAG
CDK2	GGAGTTGTGTACAAAGCCAGAA	ACGTGTCCAGGCGGATTTT
CDK4	TGTGACCAGCTGCCAAAGAG	AGAGCAATGTCAAGCGGTCA
CUL4A	AGAGCAATGTCAAGCGGTCA	TCACCTGGTAGAGCTCCTCG
E2F3	CACTTCCTCCTGCTCGCC	GAGGAGAGGGAGGGTGGC
MCM7	GCCATCGCTTCCGCTCTTA	CGGTGTCTCTGGGTGTGATG
POLD2	GAGCTTTTGGCTCGAACGTG	TGAGTGGGGTCCAGGGAAAC
POLE4	CTAGGCGCGTGGGAAGAG	CCACACCCTGTGTAGGCG
ATM	AAAACCACAGCAGGAACCAC	TCCAAGTCTGAGGACGGAAG
MRE11A	CTTGTACGACTGCGAGTGGA	TTCACCCATCCCTCTTTCTG
RAD50	CTTTGGGATCATTGCCCTGTG	CGAAGTGGTGGTCTTGTTGCT
RAD51	CACTCTGTCGCCCAGGC	TCATCTTGGGCCAGGTG
XRCC3	ATCTGCACGGAGGGATGGTT	GCCAACCGGTGAGTCTGTTATC
Desert	CTAGGAGGGTGGAGGTAGGG	GCCCCAAACAGGAGTAATGA

Supplementary Table 2

qPCR Primer Sequences

	Sequence (5' to 3')	
**Target**	**Forward**	**Reverse**
CRY1	CAACCTCCATTCATCTTTCC	CTCATAGCCGACACCTTC
p21	GGCAGACCAGCATGACAGATT	GCGGATTAGGGCTTCCTCT
FAS	GGGCATCTGGACCCTCCTAC	GATAATCTAGCAACAGACGTAAGAACCA
CCND3	CTCATCAAGAGCTATCTGTTCC	TTTAAGGTCTCGGTGGAGG
CCNE1	TTATGAAGCTGTTGGATCTCTG	AATGATACAAGGCCGAAGC
CDK2	CTCATCAAGAGCTATCTGTTCC	TTTAAGGTCTCGGTGGAGG
CDK4	TGTAGACCAGGACCTAAGGA	CGCATCAGATCCTTGATCG
CUL4A	CACAGATCCTTCCGTTTAGAG	GATCATGATCATTTGTCTGCAG
BRCA1	TTTGGAGTATGCAAACAGCT	TCTGTAGCCCATACTTTGGA
BRCA2	CAAAGTTTGTGAAGGGTCGT	ACCAAGACATATCAGGATCCA
GTF2H1	CTGTCTAGAGTTGTAGCTTCC	TCTTCAGATGAGGTTGCCA
MCM7	AGTATGGGAACCAGTTGGT	ATTTACCACTTCCCTCTCCT
POLD2	AATGAGACCCTTCCTGGAG	CTTCACTCCCACTCCACTG
POLD4	GTTGTGAAGAGGAGGGAGG	TAGAGATGCCAGAGACGGT
POLE4	CTGTTTGTGGAGACCATTGC	GGCAATCAATCTAAAGTACCTTCC
PTTG1	TGGACCTTCAATCAAAGCC	TTTAGGTAAGGCTGGTGGG
RFC3	GAGATAATAATGAAGGGCCTTCTC	TAGTAAGCTGCCATTTGTGC
TEX15	GAATACTCGTGAAGTCAATCCT	ACGTGCATCTATTCTTTCTCAG
ATM	GCGTTGCTTCTTCCTCCAGA	ATCACTGTCACTGCACTCGG
MRE11A	AGAAGATAGACATTAGTCCGGT	CATCTGGAATGGATCCTAAACC
RAD50	GAGATGGAGCAGTTAAACCA	GTTCATCTTTGTCAGCTTTGTC
RAD51	TCACGGTTAGAGCAGTGTG	AACAGCCTCCACAGTATGG
RAD54B	TGCCATTAAGACAACTACAGC	TCATTCTGAATTGGAGTACCAG
XRCC3	CATCCTTACAGCACTGCAG	TTCCGTGCAGATGTAGACG
