# Supplementary material for: The circadian cryptochrome, CRY1, is a pro-tumorigenic factor that rhythmically modulates DNA repair
Source: Nat Commun. 2021 Jan 15;12:401. doi: 10.1038/s41467-020-20513-5 (PMC7810852; doi:10.1038/s41467-020-20513-5)
Supplement: Supplementary file 3 — Reporting Summary [file 41467_2020_20513_MOESM3_ESM.pdf]

nature research

Corresponding author(s): Karen E. Knudsen

Last updated by author(s): 2020-11-11

Reporting Summary

Nature Research wishes to improve the reproducibility of the work that we publish. This form provides structure for consistency and transparency in reporting. For further information on Nature Research policies, see our Editorial Policies and the Editorial Policy Checklist.

Please do not complete any field with "not applicable" or n/a. Refer to the help text for what text to use if an item is not relevant to your study. For final submission: please carefully check your responses for accuracy; you will not be able to make changes later.

Statistics

For all statistical analyses, confirm that the following items are present in the figure legend, table legend, main text, or Methods section.

n/a	Confirmed	
☐	☒	The exact sample size (n) for each experimental group/condition, given as a discrete number and unit of measurement
☐	☒	A statement on whether measurements were taken from distinct samples or whether the same sample was measured repeatedly
☐	☒	The statistical test(s) used AND whether they are one- or two-sided<br>*Only common tests should be described solely by name; describe more complex techniques in the Methods section.*
☐	☒	A description of all covariates tested
☐	☒	A description of any assumptions or corrections, such as tests of normality and adjustment for multiple comparisons
☐	☒	A full description of the statistical parameters including central tendency (e.g. means) or other basic estimates (e.g. regression coefficient) AND variation (e.g. standard deviation) or associated estimates of uncertainty (e.g. confidence intervals)
☐	☒	For null hypothesis testing, the test statistic (e.g. F, t, r) with confidence intervals, effect sizes, degrees of freedom and P value no<br>*Give P values as exact values whenever suitable.*
☒	☐	For Bayesian analysis, information on the choice of priors and Markov chain Monte Carlo settings
☒	☐	For hierarchical and complex designs, identification of the appropriate level for tests and full reporting of outcomes
☐	☒	Estimates of effect sizes (e.g. Cohen's d, Pearson's r), indicating how they were calculated

Our web collection on statistics for biologists contains articles on many of the points above.

Software and code

Policy information about availability of computer code

Data collection	Clinical sample ChIP-seq data. AR ChIP-seq data from normal prostate tissue (n=8) and primary prostate tumor samples (n=8) were merged with samtools (v1.5) Then, the samtools views function was utilized to generate merged files with comparable read numbers Sample profiles were then plotted with the deepTools plotProfile function as described in the methods section.
Data analysis	ChIP-Seq data analyses used FASTQC v0.11.5, bowtie2 v2.3.2, MACS2 v2.1.1, deepTools v2.5.7, & Homer v4.10.3. RNA-Seq data analyses used STAR v2.5.2a and DESeq2 v1.12.4 as described in the methods section.

For manuscripts utilizing custom algorithms or software that are central to the research but not yet described in published literature, software must be made available to editors and reviewers. We strongly encourage code deposition in a community repository (e.g. GitHub). See the Nature Research guidelines for submitting code & software for further information.

Data

Policy information about availability of data

All manuscripts must include a data availability statement. This statement should provide the following information, where applicable:

- Accession codes, unique identifiers, or web links for publicly available datasets
- A list of figures that have associated raw data
- A description of any restrictions on data availability

The datasets generated during the current study have been deposited in public repositories. ChIP-Seq data have been deposited in the NCBI Gene Expression Omnibus (GEO) with the accession code GSE144960. RNA-Seq data have been deposited in the NCBI GEO with the accession code GSE144961. Molecular Signature Database was utilized for pathway analyses. The source data underlying Figs. 1B, 3C-G, 4A-E, 5B-C, 6A, 6C-D, and 7A-B and Supplemental Figs. 3D, 5C, 6D-E, 7B-C, and 7F are provided as a Source Data file.

Field-specific reporting

Please select the one below that is the best fit for your research. If you are not sure, read the appropriate sections before making your selection.

- [x] Life sciences
- [ ] Behavioural & social sciences
- [ ] Ecological, evolutionary & environmental sciences

For a reference copy of the document with all sections, see nature.com/documents/nr-reporting-summary-flat.pdf

Life sciences study design

All studies must disclose on these points even when the disclosure is negative.

Sample size

All experiments were performed in technical triplicate with at least 3 independent biological replicates per condition. Data are displayed as mean ± standard error of the mean (SEM). Statistical significance ($p < 0.05$) was determined using Student's t-test, one-way ANOVA, and two-way ANOVA on GraphPad Prism Software as appropriate and indicated in applicable figure legends.

Data exclusions

No data was excluded from the analyses in this study.

Replication

All experiments in this study were performed at least 3 independent times for biological replicates with at least technical triplicates for each individual biological replicate. The details for each specific experiment are described in the methods section and in each figure legend.

Randomization

For xenograft studies, mice were randomized into the two categories as described in the methods section. For in vitro studies, conditions were randomized into control and experimental conditions as described in each assay.

Blinding

Blinding was not relevant to majority of the assays performed in this study since they were in vitro cell culture and in vivo xenograft studies in which the treatment groups needed to be clear when performing the experiments. For the PDE (patient-derived explant) studies, deidentified tissues was utilized for examination of CRY1 expression levels in non-neoplastic, tumor, and IR treated samples. Investigators were blinded to patient tissue identifiers and were given matched tumor and non-neoplastic tissue to treat accordingly.

Behavioural & social sciences study design

All studies must disclose on these points even when the disclosure is negative.

Study description

N/A

Research sample

N/A

Sampling strategy

N/A

Data collection

N/A

Timing

N/A

Data exclusions

N/A

Non-participation

N/A

Randomization

N/A

Ecological, evolutionary & environmental sciences study design

All studies must disclose on these points even when the disclosure is negative.

Study description

N/A

Research sample

N/A

Research sample	N/A
Sampling strategy	N/A
Data collection	N/A
Timing and spatial scale	N/A
Data exclusions	N/A
Reproducibility	N/A
Randomization	N/A
Blinding	N/A

Did the study involve field work? ☐ Yes ☒ No

Field work, collection and transport

Field conditions	N/A
Location	N/A
Access & import/export	N/A
Disturbance	N/A

Reporting for specific materials, systems and methods

We require information from authors about some types of materials, experimental systems and methods used in many studies. Here, indicate whether each material, system or method listed is relevant to your study. If you are not sure if a list item applies to your research, read the appropriate section before selecting a response.

Materials & experimental systems

n/a	Involved in the study	
☐	☒	Antibodies
☐	☒	Eukaryotic cell lines
☒	☐	Palaeontology and archaeology
☐	☒	Animals and other organisms
☐	☒	Human research participants
☒	☐	Clinical data
☒	☐	Dual use research of concern

Methods

n/a	Involved in the study	
☐	☒	ChIP-seq
☐	☒	Flow cytometry
☒	☐	MRI-based neuroimaging

Antibodies

Antibodies used	CRY1 (Bethyl A302-614A), ATM (Cell Signaling Technology (CST) 2873), phospho-ATM (Ser1981) (CST 5883S), CHK2 (Bethyl A300-619A), phospho-CHK2 (Thr68) (CST 2661T), MRE11 (CST 8344T), RAD50 (CST 8344T), RAD51 (Abcam ab63801), XRCC3 (Novus NB100-165), and Vinculin (Sigma-Aldrich V9264).
Validation	The antibodies listed above are all commercially available and were used at 1:1000 dilution per manufacturer's instructions and protocols. Bethly, Cell Signaling Technology, Abcam, Novus, and Sigma-Alridch validated their antibodies and the description can be found on their websites for specific antibodies listed above. Additionally, the commercially available antibodies used in this study were produced by immunizing animals with recombinant human CRY1, ATM, pATM, CHK2, pCHK2, MRE11, RAD50, RAD51, XRCC3, or Vinculin.

Eukaryotic cell lines

Policy information about cell lines

Cell line source(s)	C4-2, 22Rv1, and LNCaP cells were purchased from ATCC.

Authentication	C4-2, 22Rv1, and LNCaP cells were purchased from ATCC and authenticated directly by ATCC using their morphology, karyotyping, and PCR based approaches to confirm the identity of human cell lines.
Mycoplasma contamination	All cell lines utilized in this study (i.e. C4-2, 22Rv1, and LNCaP cells and their inducible shCON and shCRY1 lines) were all tested for mycoplasma upon thawing of cells. All tests were negative for myco contamination.
Commonly misidentified lines (See ICLAC register)	Cells were authenticated directly by ATCC. No commonly misidentified lines were used in this study.

Palaeontology and Archaeology

Specimen provenance	N/A
Specimen deposition	N/A
Dating methods	N/A

☐ Tick this box to confirm that the raw and calibrated dates are available in the paper or in Supplementary Information.

Ethics oversight	N/A

Note that full information on the approval of the study protocol must also be provided in the manuscript.

Animals and other organisms

Policy information about studies involving animals; ARRIVE guidelines recommended for reporting animal research

Laboratory animals	Nude, SCID, male mice at least 8 weeks of age were utilized for xenograft studies as described in the methods section.
Wild animals	No wild animals were used in this study.
Field-collected samples	No field collected samples were used in this study.
Ethics oversight	The Institutional Animal Care and Use Committee (IACUC) at Thomas Jefferson University approved all protocols for this study

Note that full information on the approval of the study protocol must also be provided in the manuscript.

Human research participants

Policy information about studies involving human research participants

Population characteristics	Patient characteristics for data for ChIP-Seq and survival data is described in detail for each specific cohort (JHMI, Decipher, Netherlands, Pomerantz, cBioPortal) in the methods section with tables providing the specific characteristics.
Recruitment	Samples used in this study are from previously published cohorts (JHMI, Decipher, Netherlands, Pomerantz, cBioPortal) and their recruitment was not applicable to this study.
Ethics oversight	The use of patient and clinical material was approved by the ethical committees from each of the following institutes: the Sidney Kimmel Cancer Center at Thomas Jefferson University (Pennsylvania, USA), the Department of Radiation Oncology at the University of California at San Francisco (California, USA), and Division of Oncogenomics in the Oncode Institute and the Netherlands Cancer Institute (Amsterdam, The Netherlands).

Note that full information on the approval of the study protocol must also be provided in the manuscript.

Clinical data

Policy information about clinical studies

All manuscripts should comply with the ICMJE guidelines for publication of clinical research and a completed CONSORT checklist must be included with all submissio

Clinical trial registration	N/A
Study protocol	N/A
Data collection	N/A
Outcomes	N/A

Dual use research of concern

Policy information about dual use research of concern

Hazards

Could the accidental, deliberate or reckless misuse of agents or technologies generated in the work, or the application of information presented in the manuscript, pose a threat to:

No	Yes	
■	☐	Public health
■	☐	National security
■	☐	Crops and/or livestock
■	☐	Ecosystems
■	☐	Any other significant area

Experiments of concern

Does the work involve any of these experiments of concern:

No	Yes	
■	☐	Demonstrate how to render a vaccine ineffective
■	☐	Confer resistance to therapeutically useful antibiotics or antiviral agents
■	☐	Enhance the virulence of a pathogen or render a nonpathogen virulent
■	☐	Increase transmissibility of a pathogen
■	☐	Alter the host range of a pathogen
■	☐	Enable evasion of diagnostic/detection modalities
■	☐	Enable the weaponization of a biological agent or toxin
■	☐	Any other potentially harmful combination of experiments and agents

ChIP-seq

Data deposition

■ Confirm that both raw and final processed data have been deposited in a public database such as GEO.

■ Confirm that you have deposited or provided access to graph files (e.g. BED files) for the called peaks.

Data access links<br>*May remain private before publication.*	https://www.ncbi.nlm.nih.gov/geo/query/acc.cgi?acc=GSE144962 with token ivwzqcmczbixxqz
Files in database submission	ChIP-Seq – Veh IP and Input for CRY1 in C4-2 cells. RNA-Seq – shCON and shCRY1 in C4-2 cells.
Genome browser session<br>(e.g. UCSC)	GSE144960 and GSE144961

Methodology

Replicates	Two biological replicates for Veh condition for CRY1 IP in C4-2 cells with corresponding input.
Sequencing depth	FASTQ files were assessed for quality using FASTQC v0 11 5 Reads were aligned to the human genome reference version hg19 using bowtie2 v2.3.2Click or tap here to enter text. with default parameters.
Antibodies	CRY1 antibody previously used for ChIP-Seq in human U2OS cells in Hoffmann, J. et al. Non-circadian expression masking clock-driven weak transcription rhythms in U2OS cells. PloS one **9**, e102238 (2014).
Peak calling parameters	Peak calling was performed using MACS2 v2.1.1[72] with combined replicates, utilizing a q < 0.05 cutoff.
Data quality	FDR > 1 5 and q < 0 05 was used for the peak calling cutoff as described in the methods section in detail.
Software	The ChIP-Seq libraries were constructed using the Swift BioSciences ACCEL-NGS 2S Plus DNA Library kit with approximately 10 ng of ChIP DNA NextSeq 500 sequencer from Illumina was utilized to sequence samples. ChIP-Seq binding heatmaps and profiles were generated using deepTools v2.5.7. Peak annotation and motif analysis performed using Homer v4 10 3[74] using the parameters indicated.

Flow Cytometry

Plots

Confirm that:

- [x] The axis labels state the marker and fluorochrome used (e.g. CD4-FITC).
- [x] The axis scales are clearly visible. Include numbers along axes only for bottom left plot of group (a 'group' is an analysis of identical markers).
- [x] All plots are contour plots with outliers or pseudocolor plots.
- [x] A numerical value for number of cells or percentage (with statistics) is provided.

Methodology

Sample preparation	All C4-2 and 22Rv1 derived cells were plated at equal densities in hormone-proficient media.
Instrument	Millipore Guava flow cytometry machine
Software	Analysis was performed using InCyte software (Guava) for cell-cycle profile with BrdU incorporation and PI (propidium iodide).
Cell population abundance	Once all treatments were completed, cells were incubated with BrdU (1:1000) for 2 hrs prior to harvesting. At least 10,000 events per sample were assessed.
Gating strategy	Gating was established to measure G0, G1, S, and G2/M phases and calculate percent of population in each phase with BrdU and PI incorporation.

- [x] Tick this box to confirm that a figure exemplifying the gating strategy is provided in the Supplementary Information.

Magnetic resonance imaging

Experimental design

Design type	N/A
Design specifications	N/A
Behavioral performance measures	N/A

Acquisition

Imaging type(s)	N/A
Field strength	N/A
Sequence & imaging parameters	N/A
Area of acquisition	N/A

Diffusion MRI [ ] Used [x] Not used

Preprocessing

Preprocessing software	N/A
Normalization	N/A
Normalization template	N/A
Noise and artifact removal	N/A

Volume censoring | N/A

Statistical modeling & inference

Model type and settings | N/A

Effect(s) tested | N/A

Specify type of analysis: ☐ Whole brain ☐ ROI-based ☐ Both

Statistic type for inference
(See Eklund et al. 2016) | N/A

Correction | N/A

Models & analysis

n/a	Involved in the study
☒	☐ Functional and/or effective connectivity
☒	☐ Graph analysis
☒	☐ Multivariate modeling or predictive analysis

Functional and/or effective connectivity | N/A

Graph analysis | N/A

Multivariate modeling and predictive analysis | N/A

his checklist template is licensed under a Creative Commons Attribution 4.0 International License, which permits use, sharing, adaptation, distribution and reproduction in any medium or format, as long as you give ppropriate credit to the original author(s) and the source, provide a link to the Creative Commons license, and indicate if changes were made. The images or other third party material in this article are included in e article's Creative Commons license, unless indicated otherwise in a credit line to the material. If material is not included in the article's Creative Commons license and your intended use is not permitted by atutory regulation or exceeds the permitted use, you will need to obtain permission directly from the copyright holder. To view a copy of this license, visit http://creativecommons.org/licenses/by/4.0/
