# Supplementary material for: The circadian cryptochrome, CRY1, is a pro-tumorigenic factor that rhythmically modulates DNA repair
Source: Nat Commun. 2021 Jan 15;12:401. doi: 10.1038/s41467-020-20513-5 (PMC7810852; doi:10.1038/s41467-020-20513-5)
Supplement: Supplementary file 4 — Source data [file 41467_2020_20513_MOESM4_ESM.zip › Full unedited gels.pptx]

## Slide 1
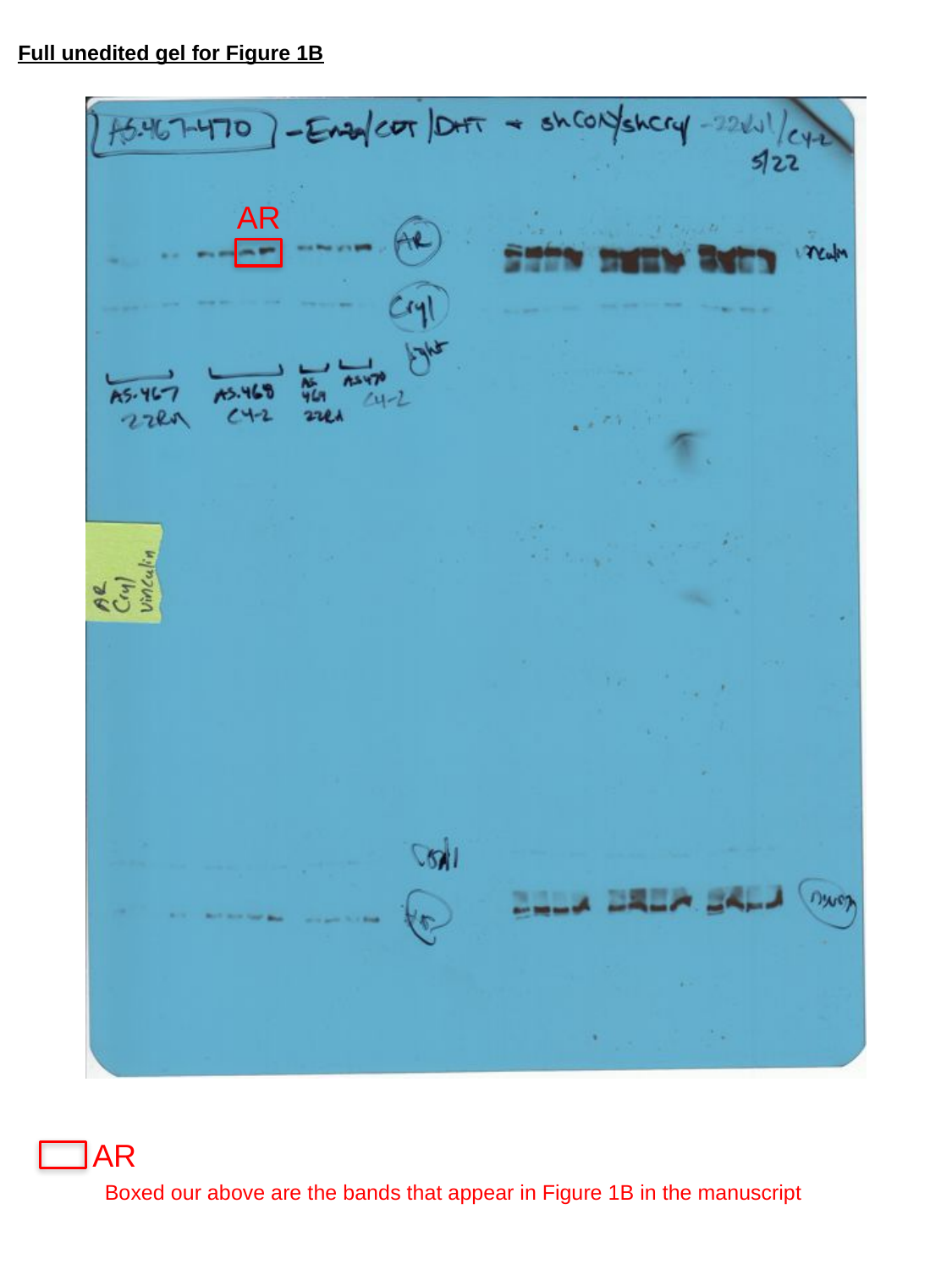

# Full unedited gel for Figure 1B
AR
AR
Boxed our above are the bands that appear in Figure 1B in the manuscript

## Slide 2
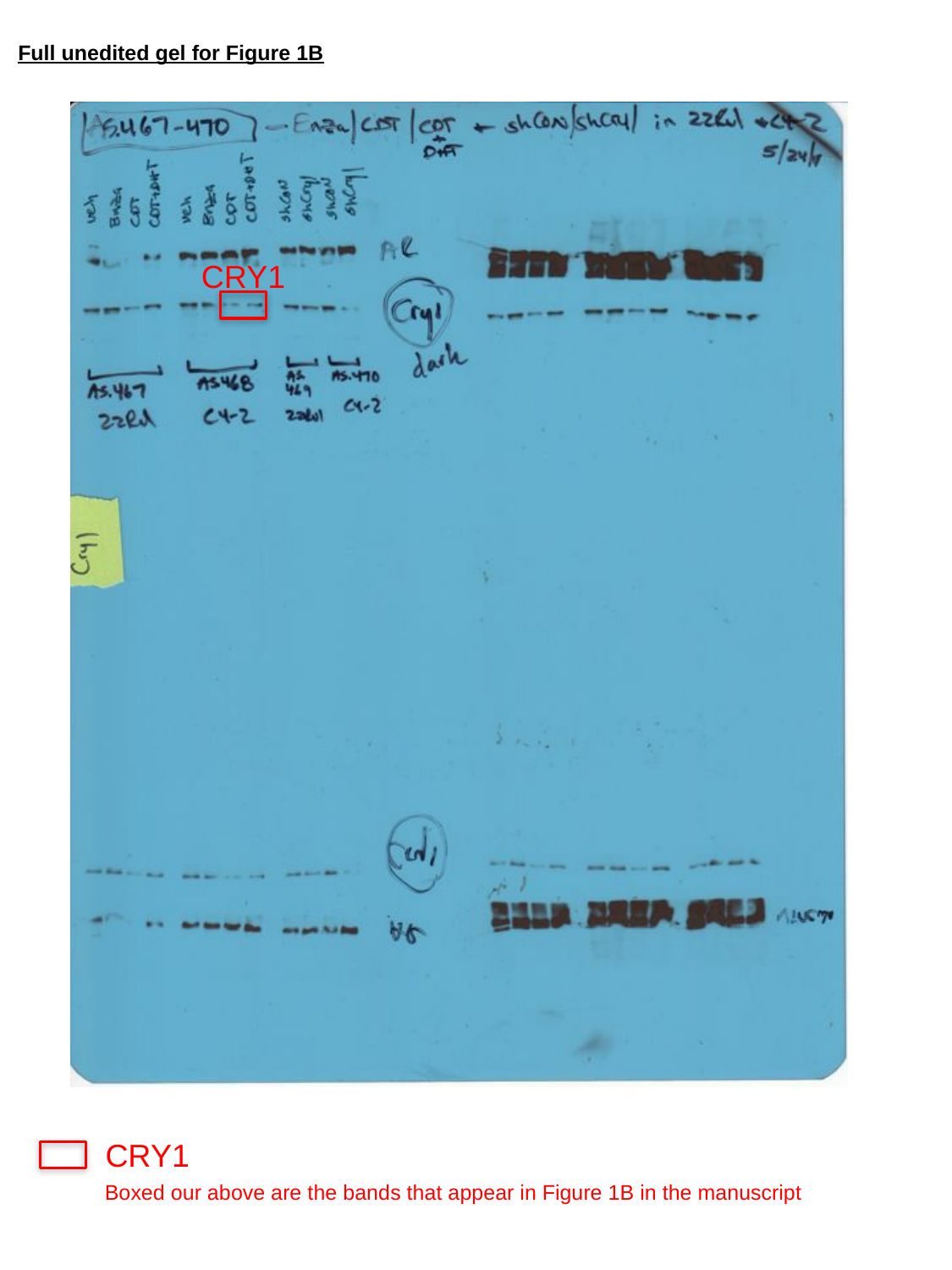

# Full unedited gel for Figure 1B
CRY1
CRY1
Boxed our above are the bands that appear in Figure 1B in the manuscript

## Slide 3
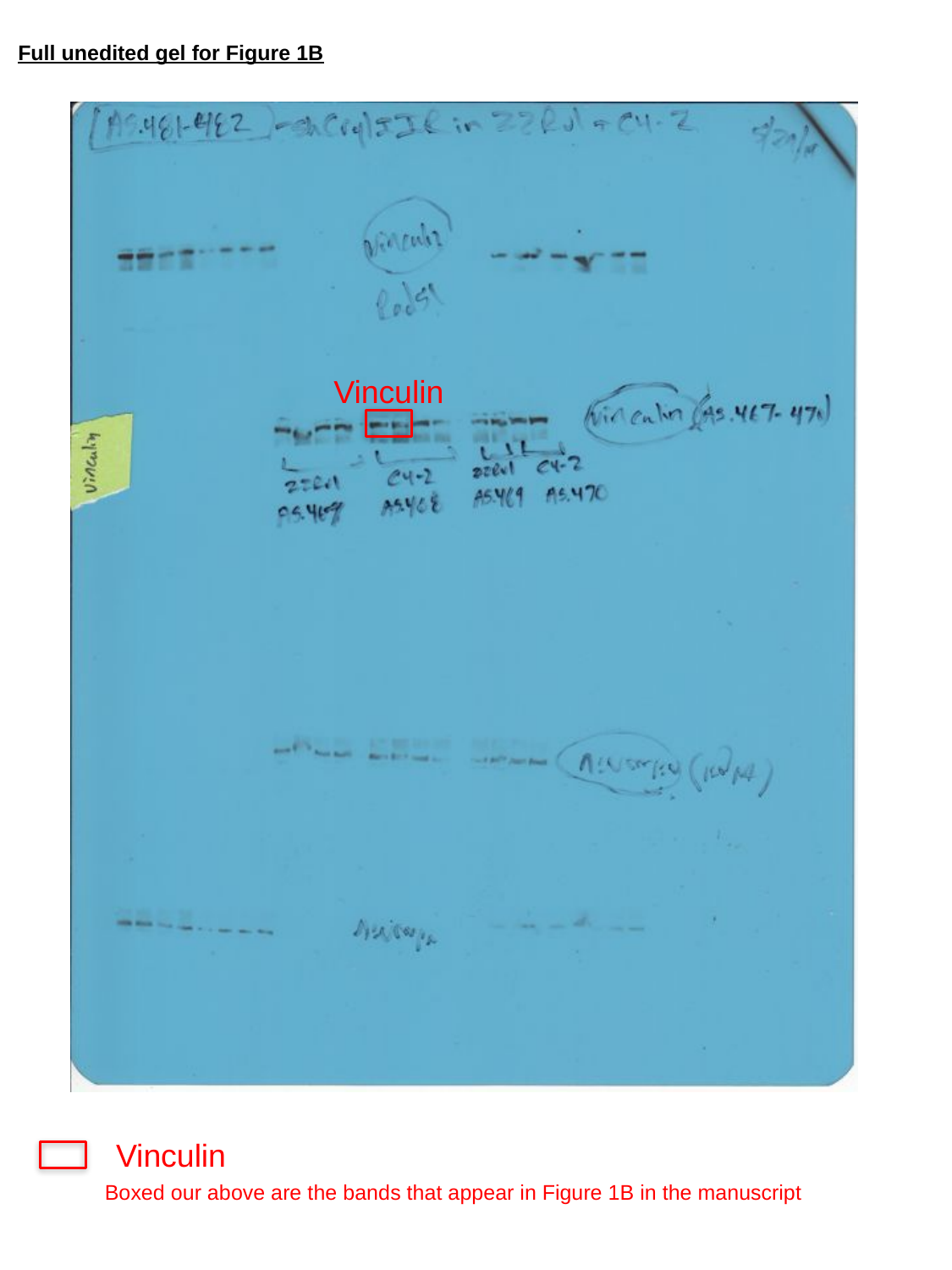

# Full unedited gel for Figure 1B
Vinculin
Vinculin
Boxed our above are the bands that appear in Figure 1B in the manuscript

## Slide 4
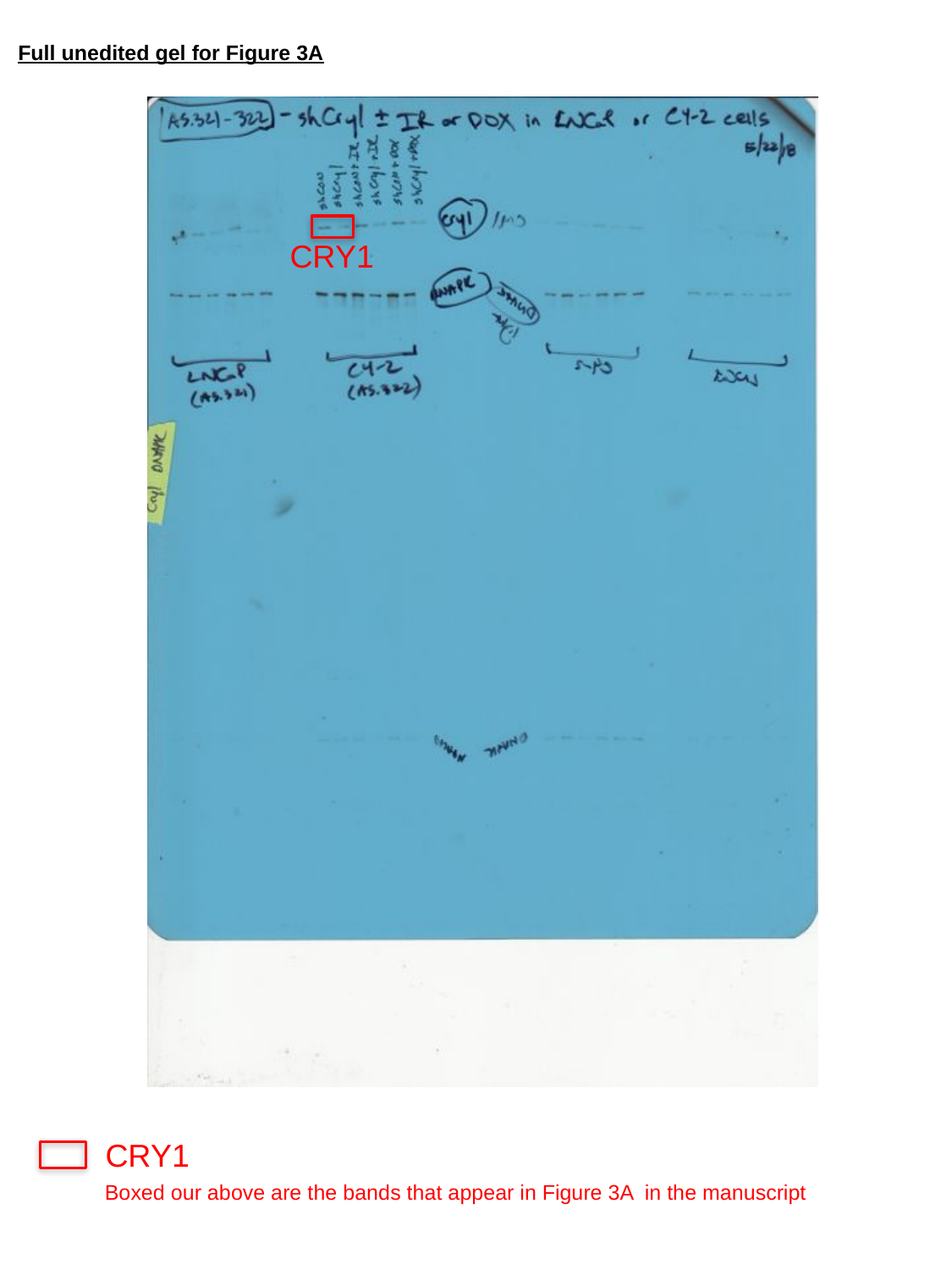

Full unedited gel for Figure 3A
CRY1
CRY1
Boxed our above are the bands that appear in Figure 3A in the manuscript

## Slide 5
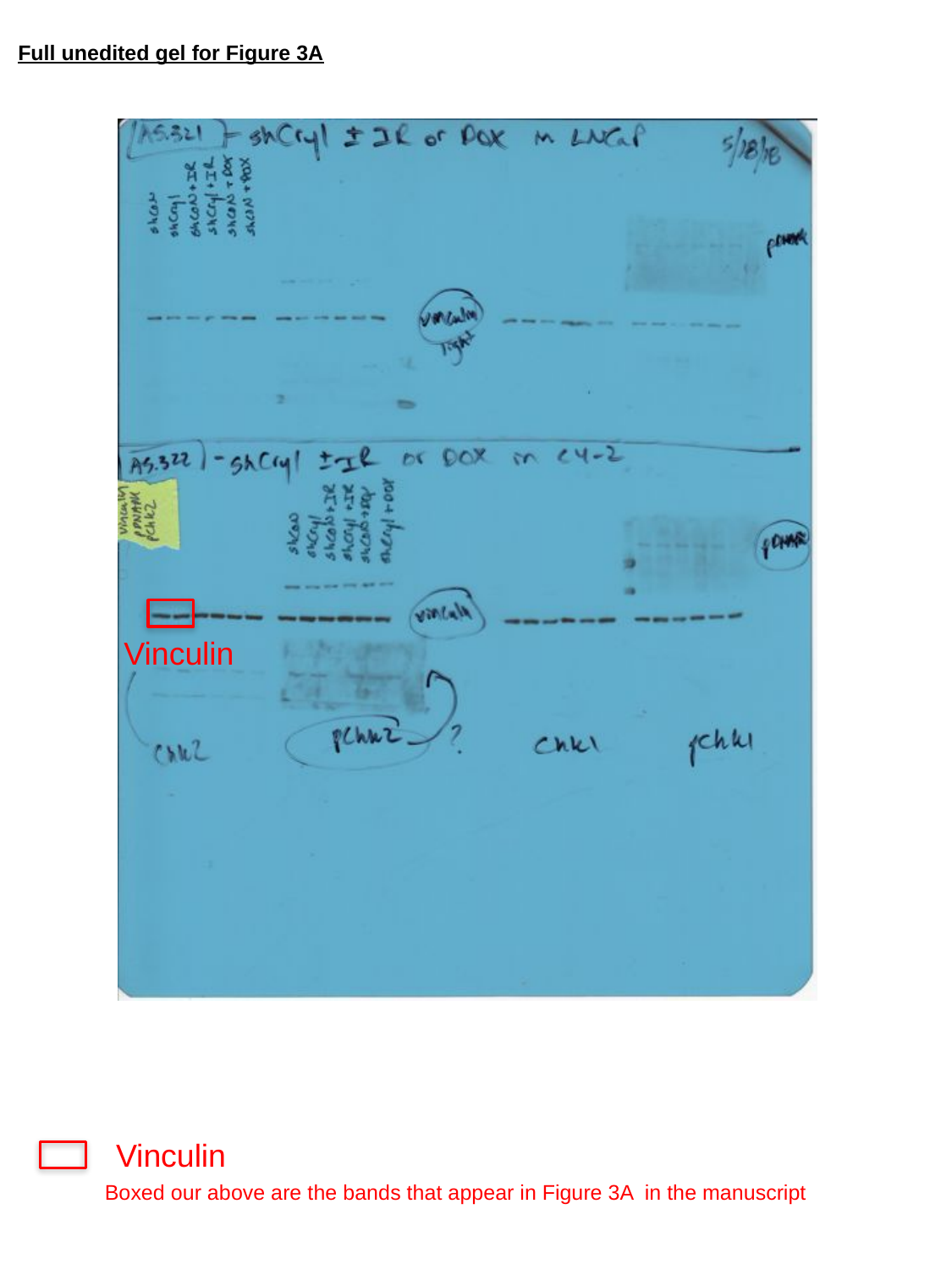

Full unedited gel for Figure 3A
Vinculin
Vinculin
Boxed our above are the bands that appear in Figure 3A in the manuscript

## Slide 6
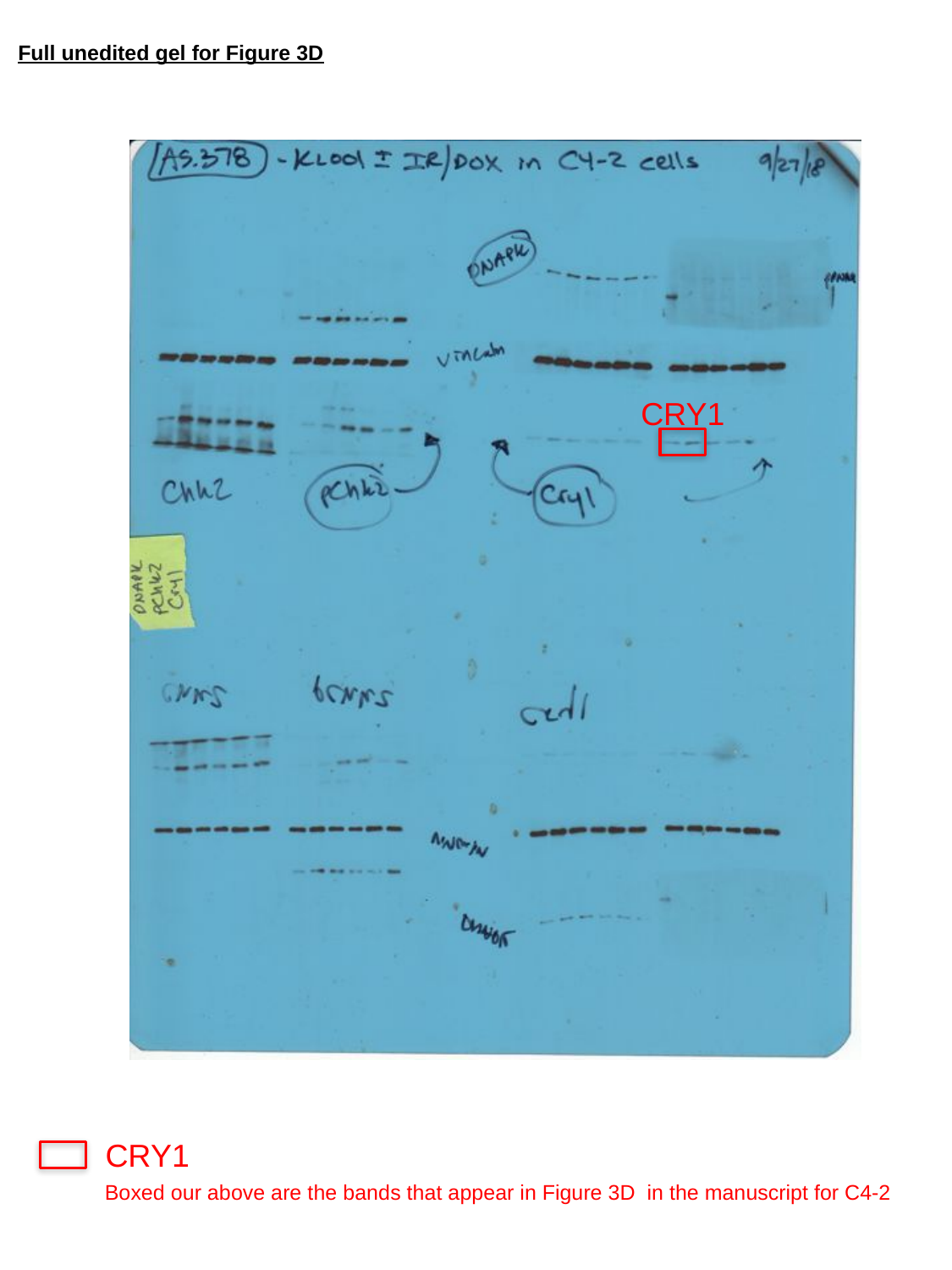

Full unedited gel for Figure 3D
CRY1
CRY1
Boxed our above are the bands that appear in Figure 3D in the manuscript for C4-2

## Slide 7
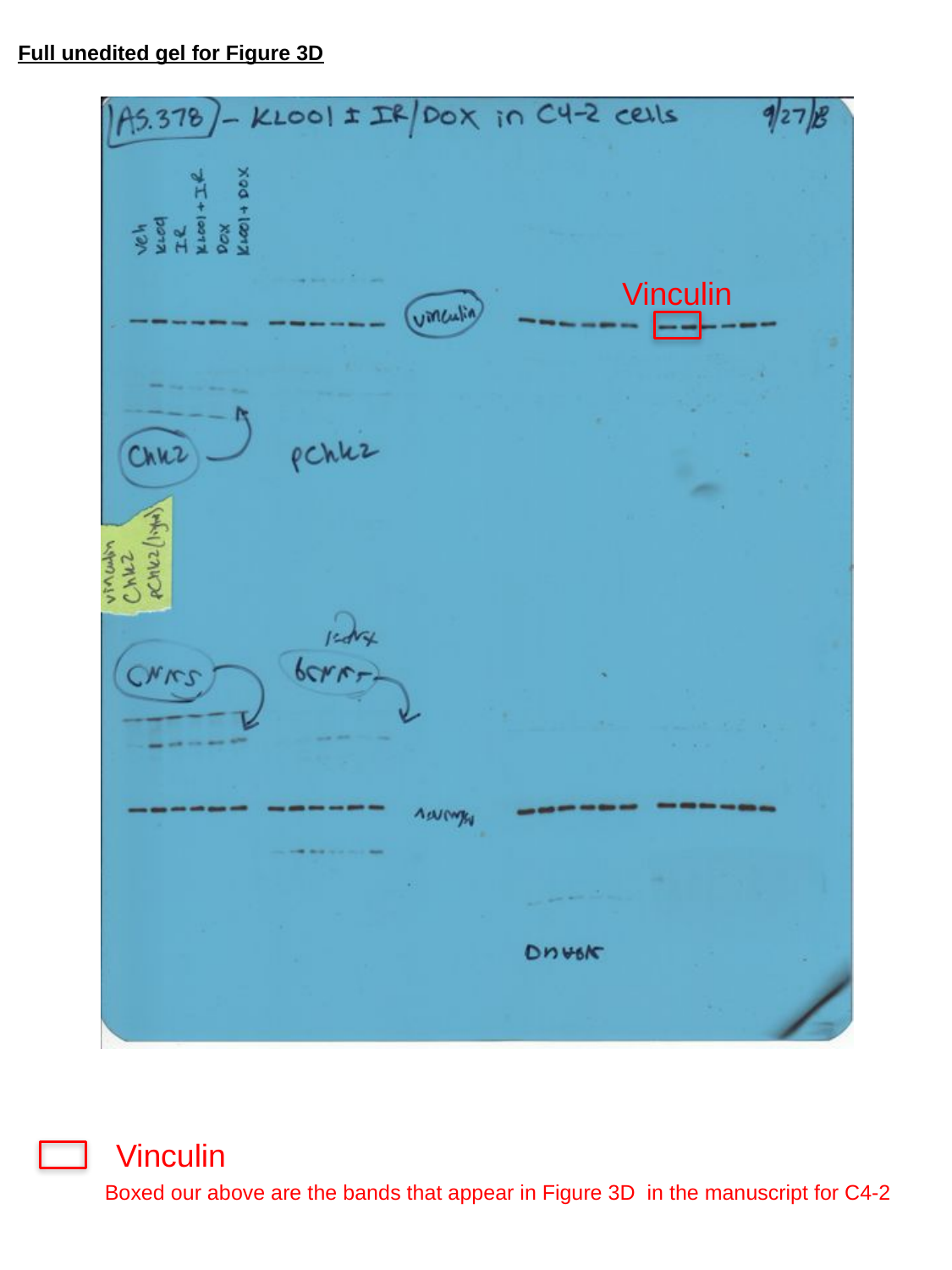

Full unedited gel for Figure 3D
Vinculin
Vinculin
Boxed our above are the bands that appear in Figure 3D in the manuscript for C4-2

## Slide 8
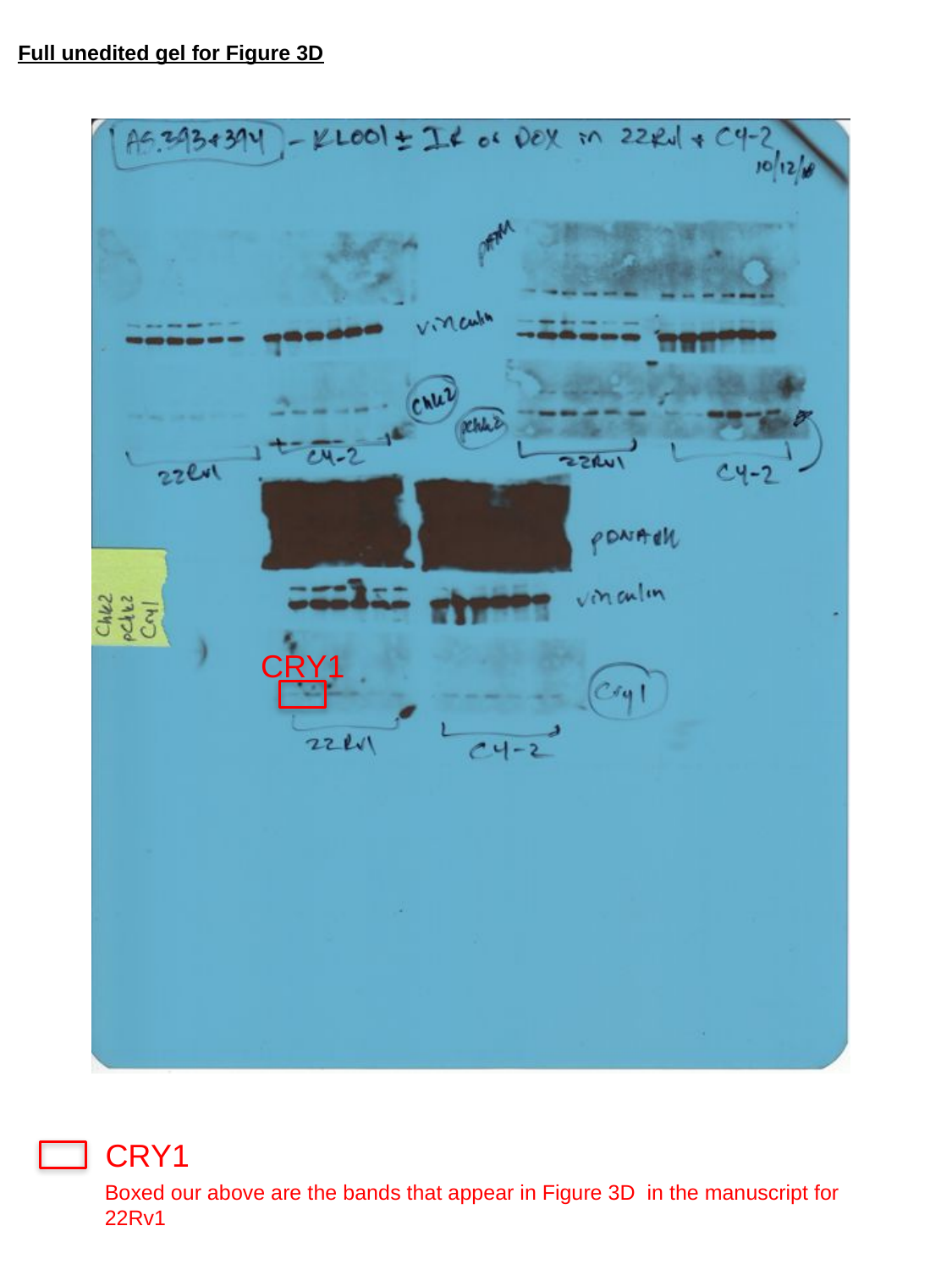

Full unedited gel for Figure 3D
CRY1
CRY1
Boxed our above are the bands that appear in Figure 3D in the manuscript for 22Rv1

## Slide 9
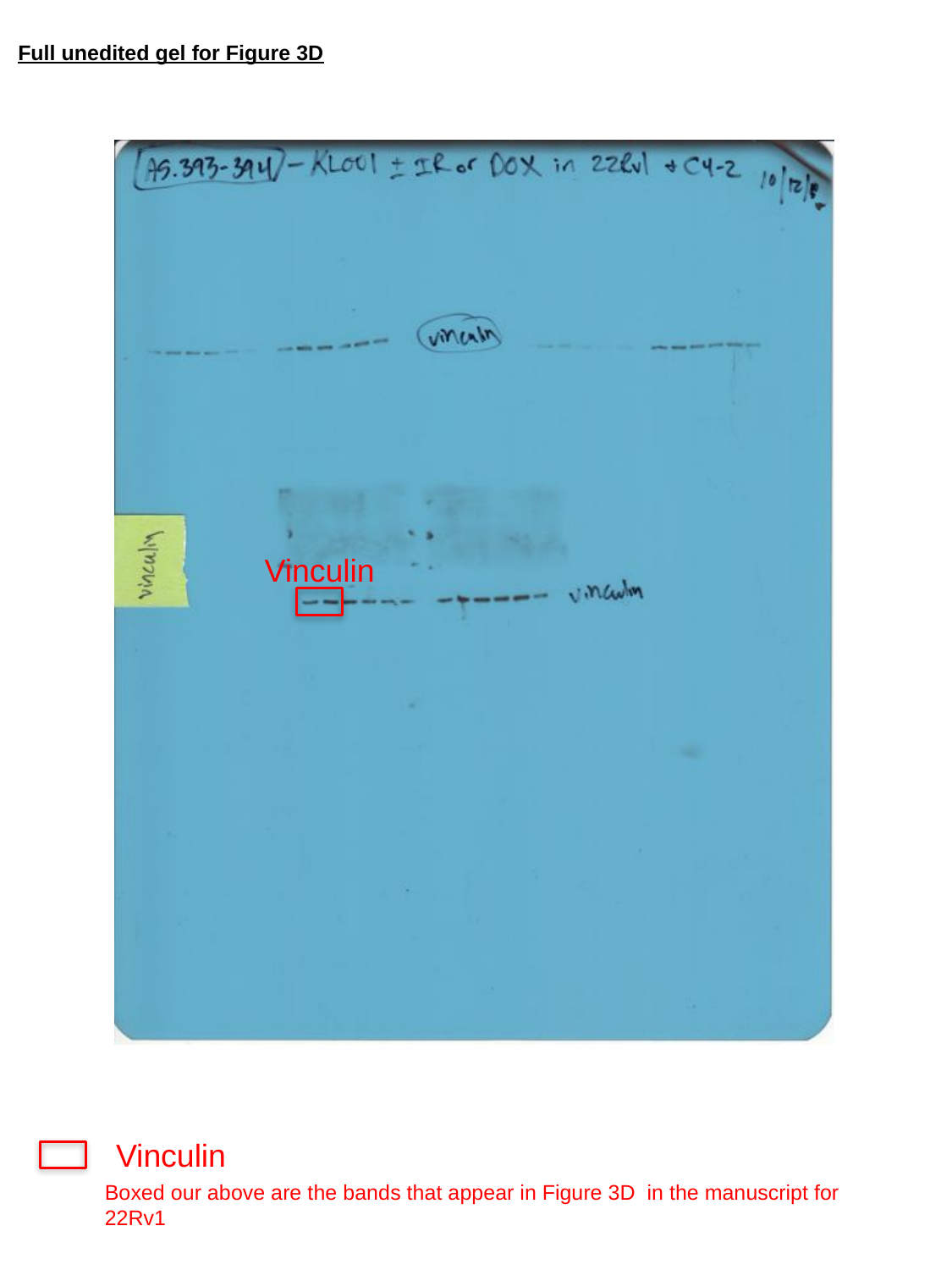

Full unedited gel for Figure 3D
Vinculin
Vinculin
Boxed our above are the bands that appear in Figure 3D in the manuscript for 22Rv1

## Slide 10
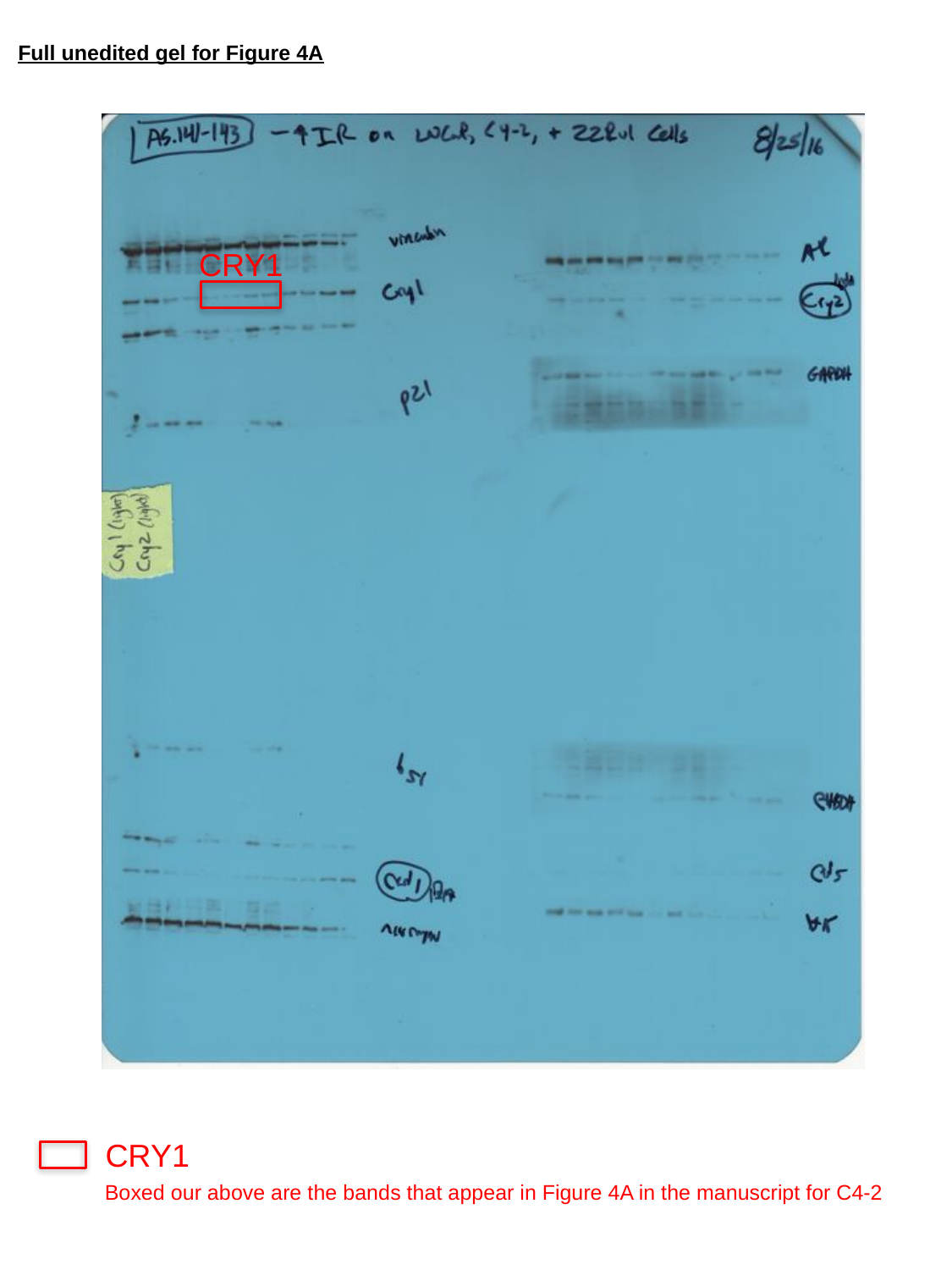

Full unedited gel for Figure 4A
CRY1
CRY1
Boxed our above are the bands that appear in Figure 4A in the manuscript for C4-2

## Slide 11
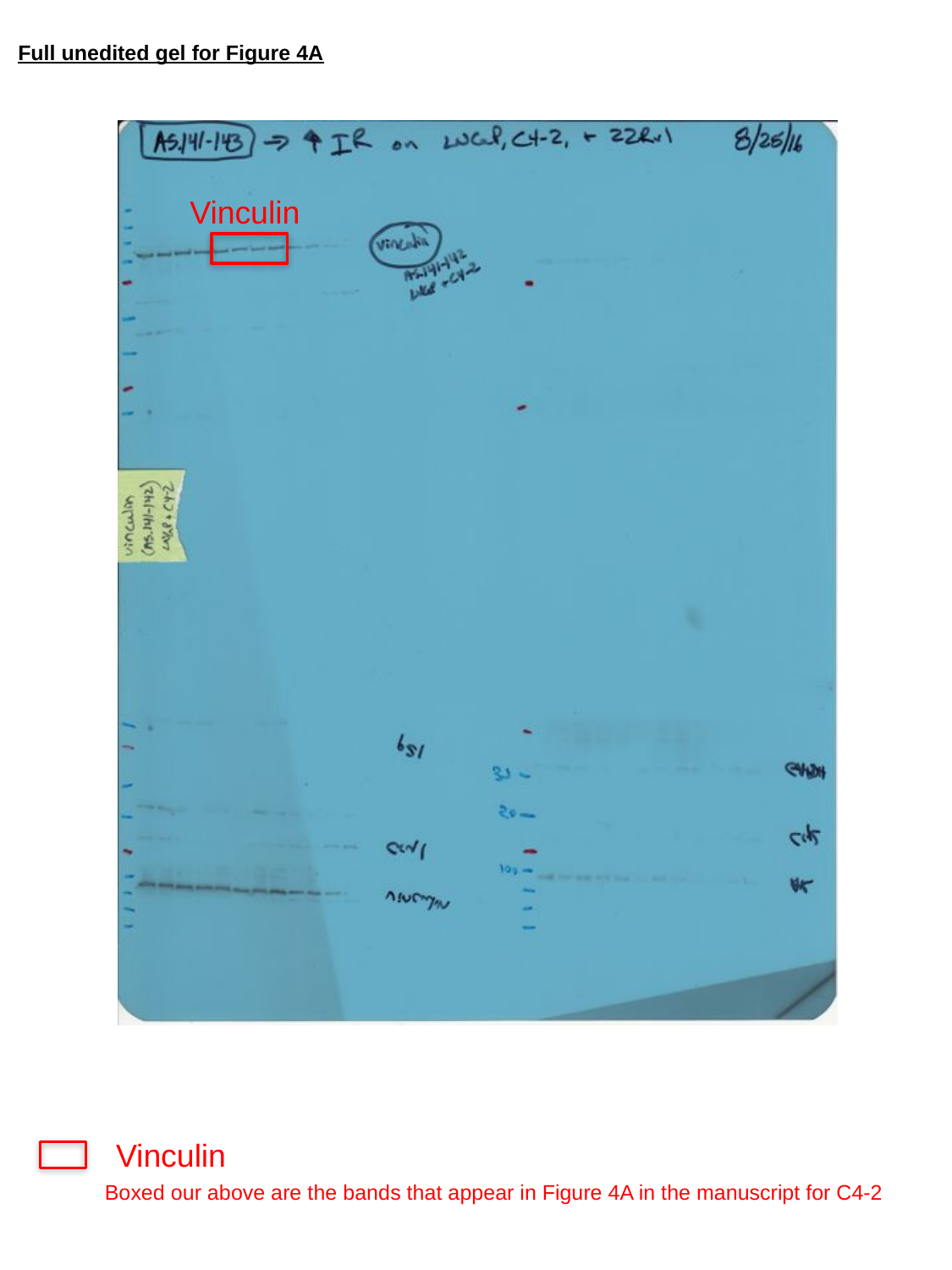

Full unedited gel for Figure 4A
Vinculin
Vinculin
Boxed our above are the bands that appear in Figure 4A in the manuscript for C4-2

## Slide 12
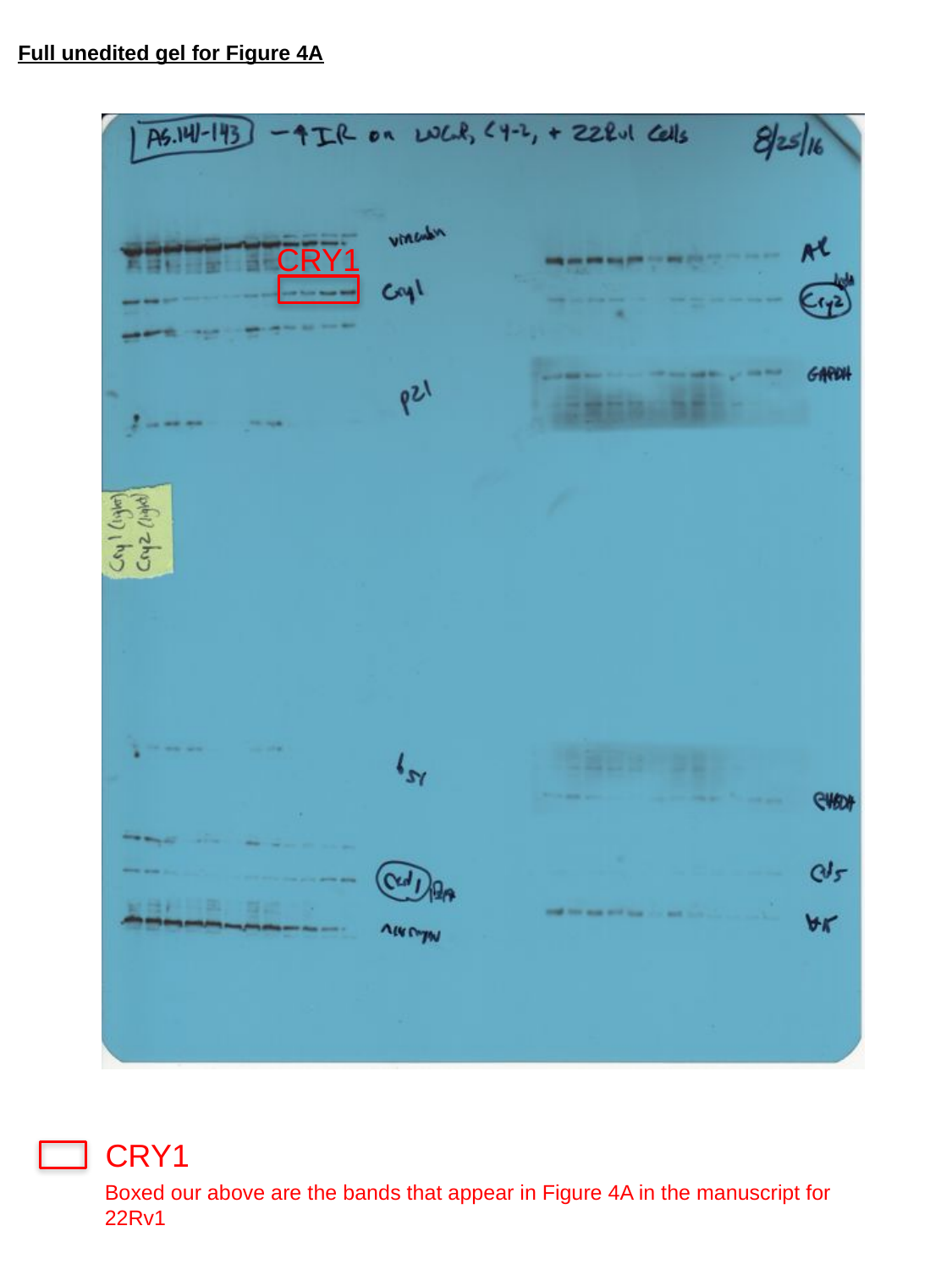

Full unedited gel for Figure 4A
CRY1
CRY1
Boxed our above are the bands that appear in Figure 4A in the manuscript for 22Rv1

## Slide 13
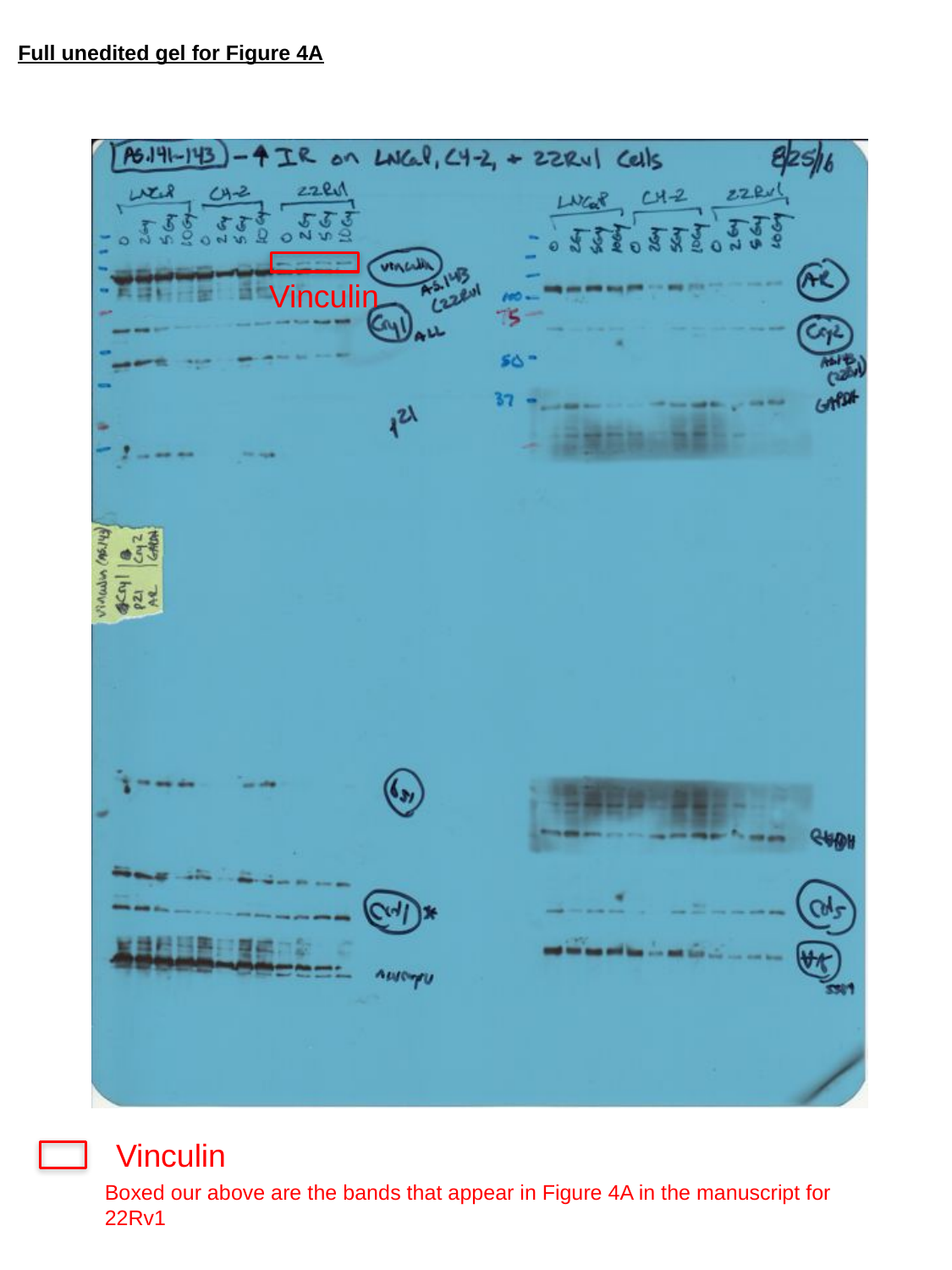

Full unedited gel for Figure 4A
Vinculin
Vinculin
Boxed our above are the bands that appear in Figure 4A in the manuscript for 22Rv1

## Slide 14
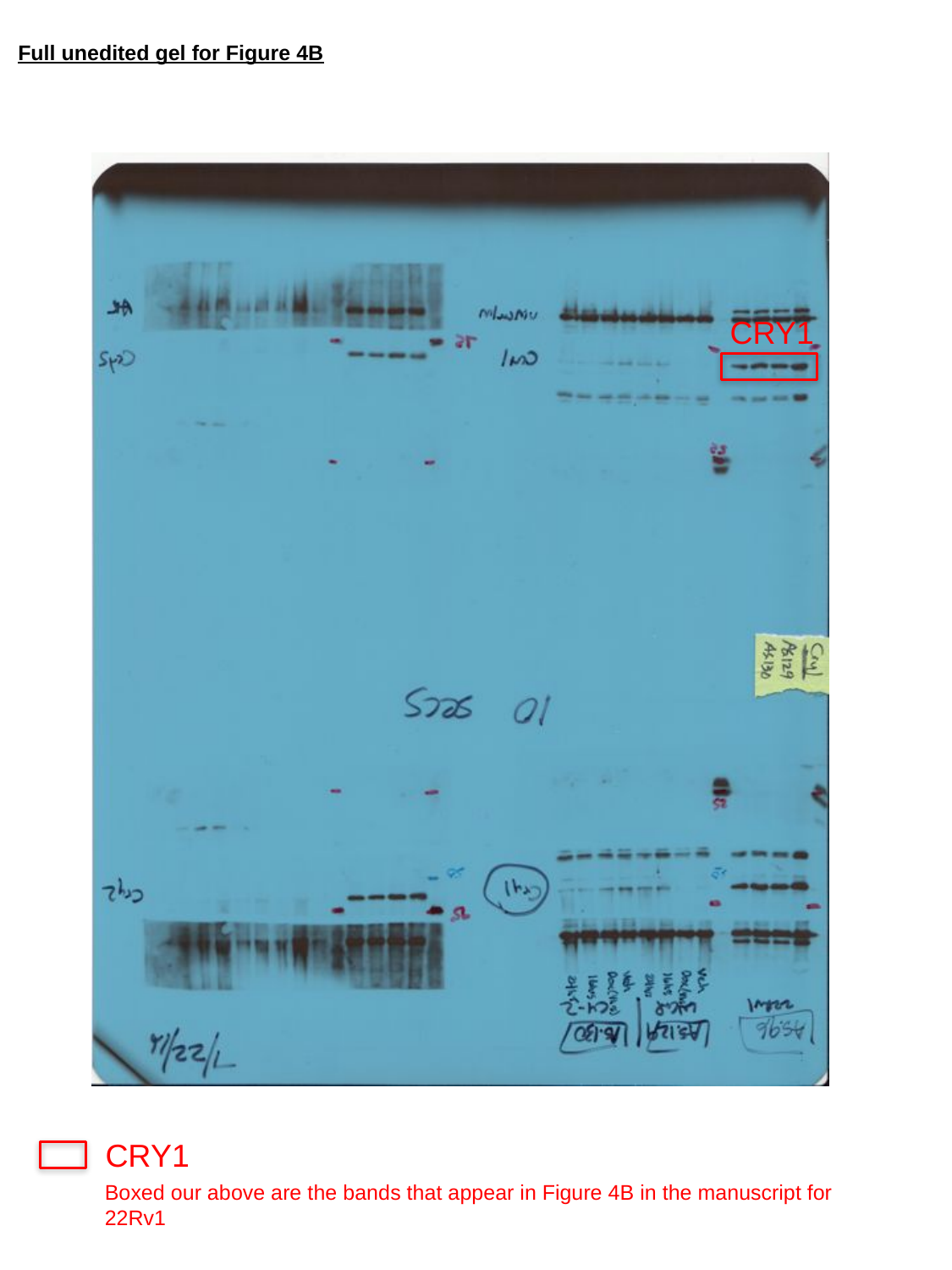

Full unedited gel for Figure 4B
CRY1
CRY1
Boxed our above are the bands that appear in Figure 4B in the manuscript for 22Rv1

## Slide 15
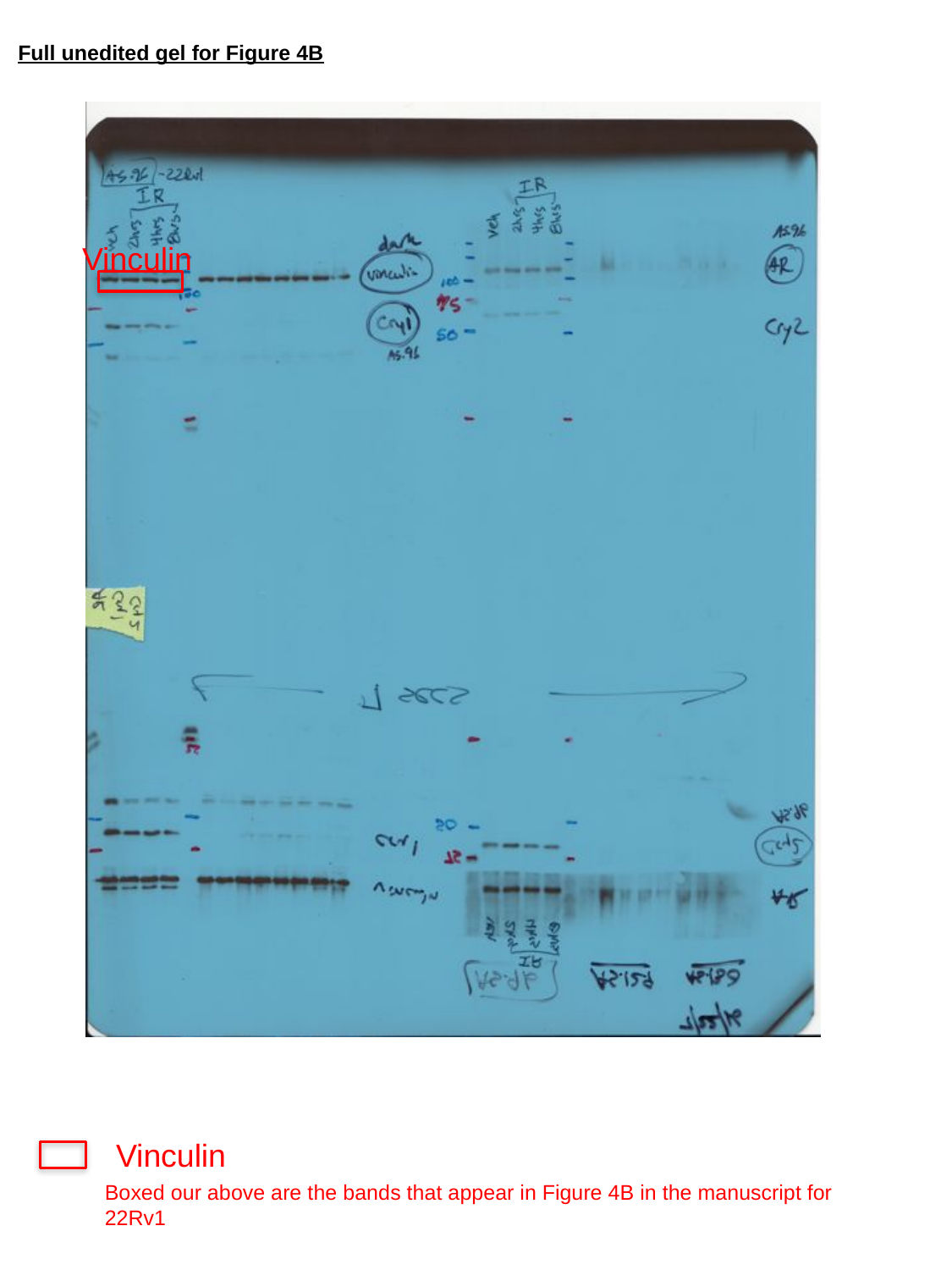

Full unedited gel for Figure 4B
Vinculin
Vinculin
Boxed our above are the bands that appear in Figure 4B in the manuscript for 22Rv1

## Slide 16
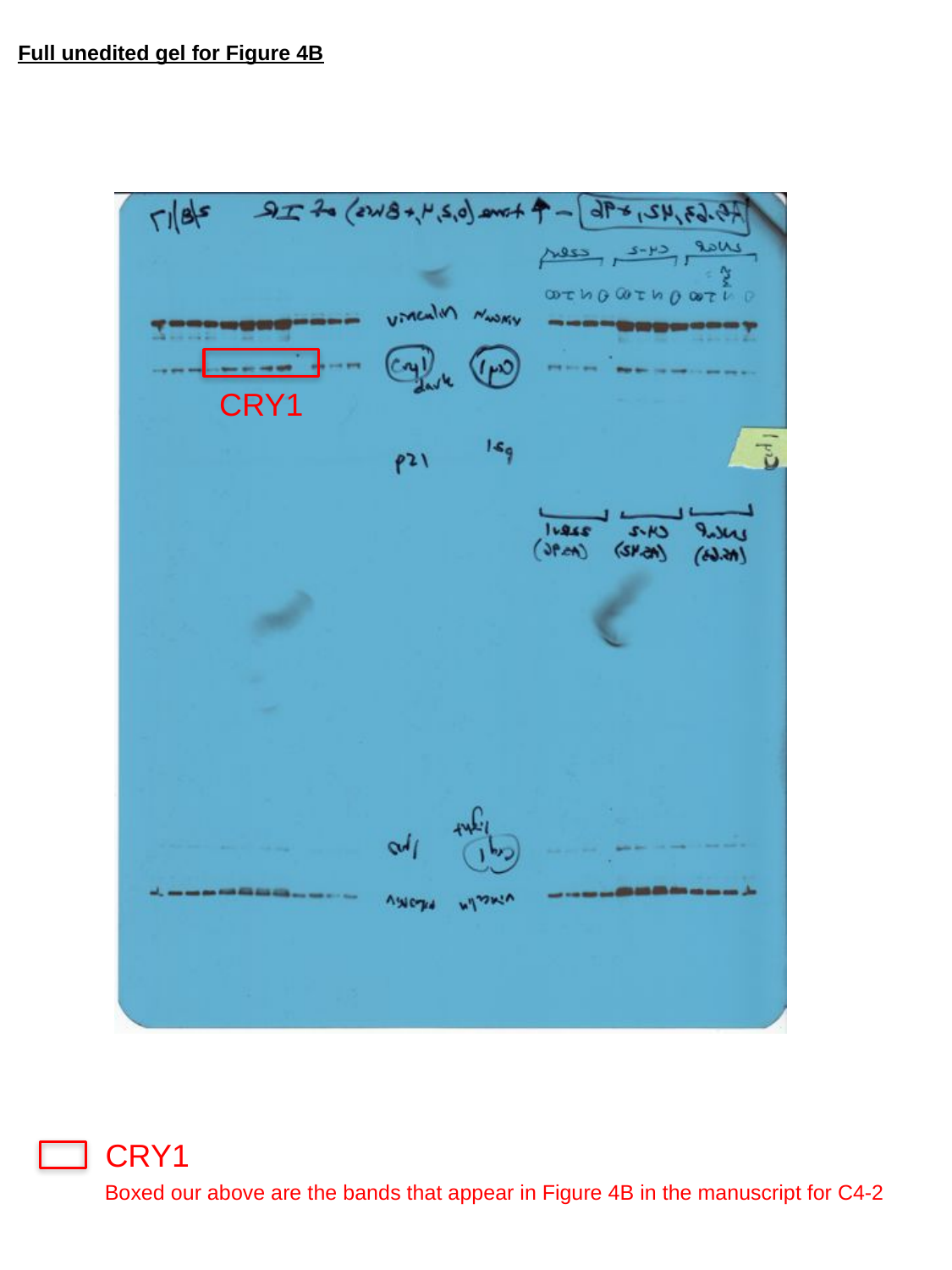

Full unedited gel for Figure 4B
CRY1
CRY1
Boxed our above are the bands that appear in Figure 4B in the manuscript for C4-2

## Slide 17
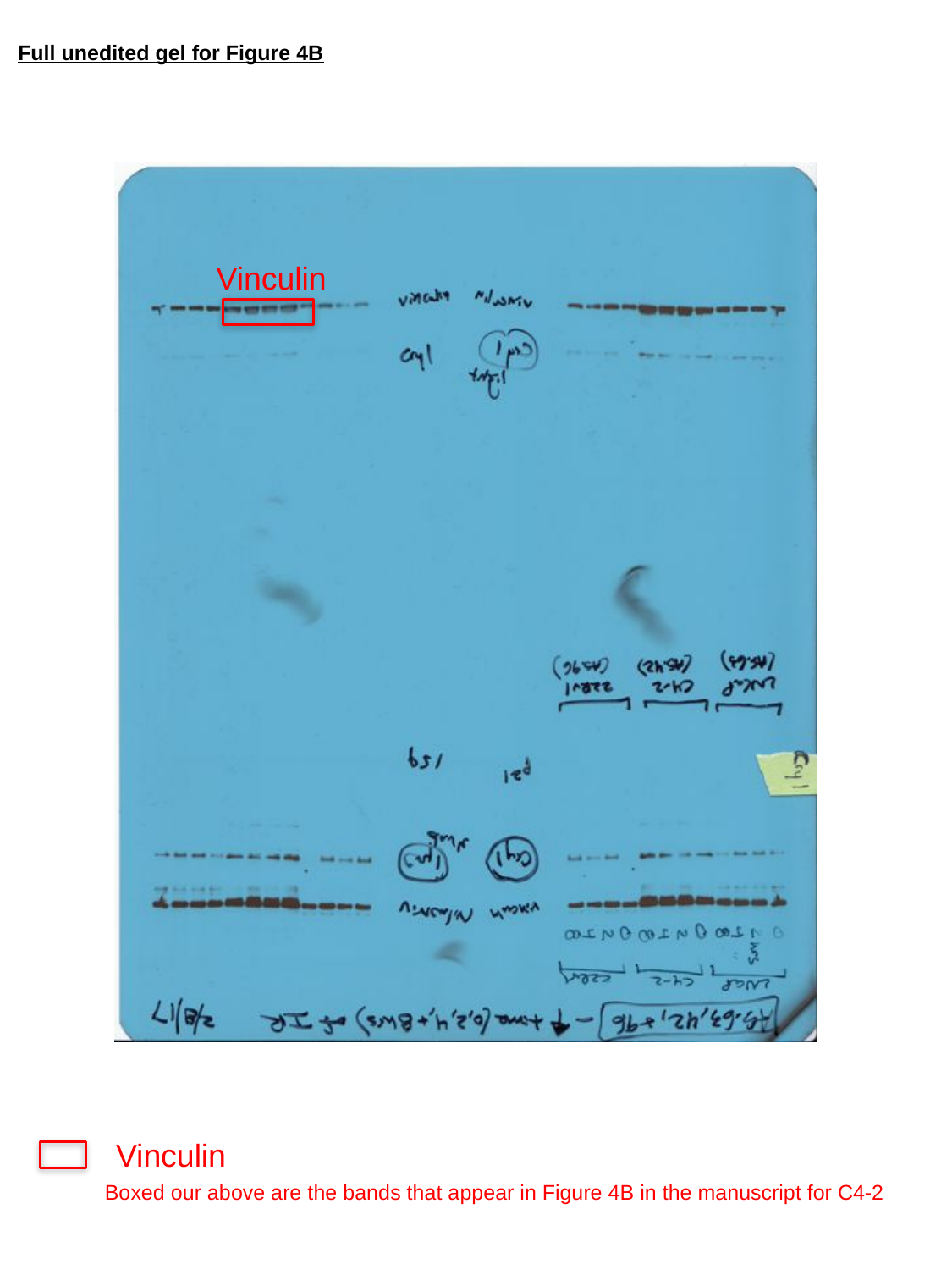

Full unedited gel for Figure 4B
Vinculin
Vinculin
Boxed our above are the bands that appear in Figure 4B in the manuscript for C4-2

## Slide 18
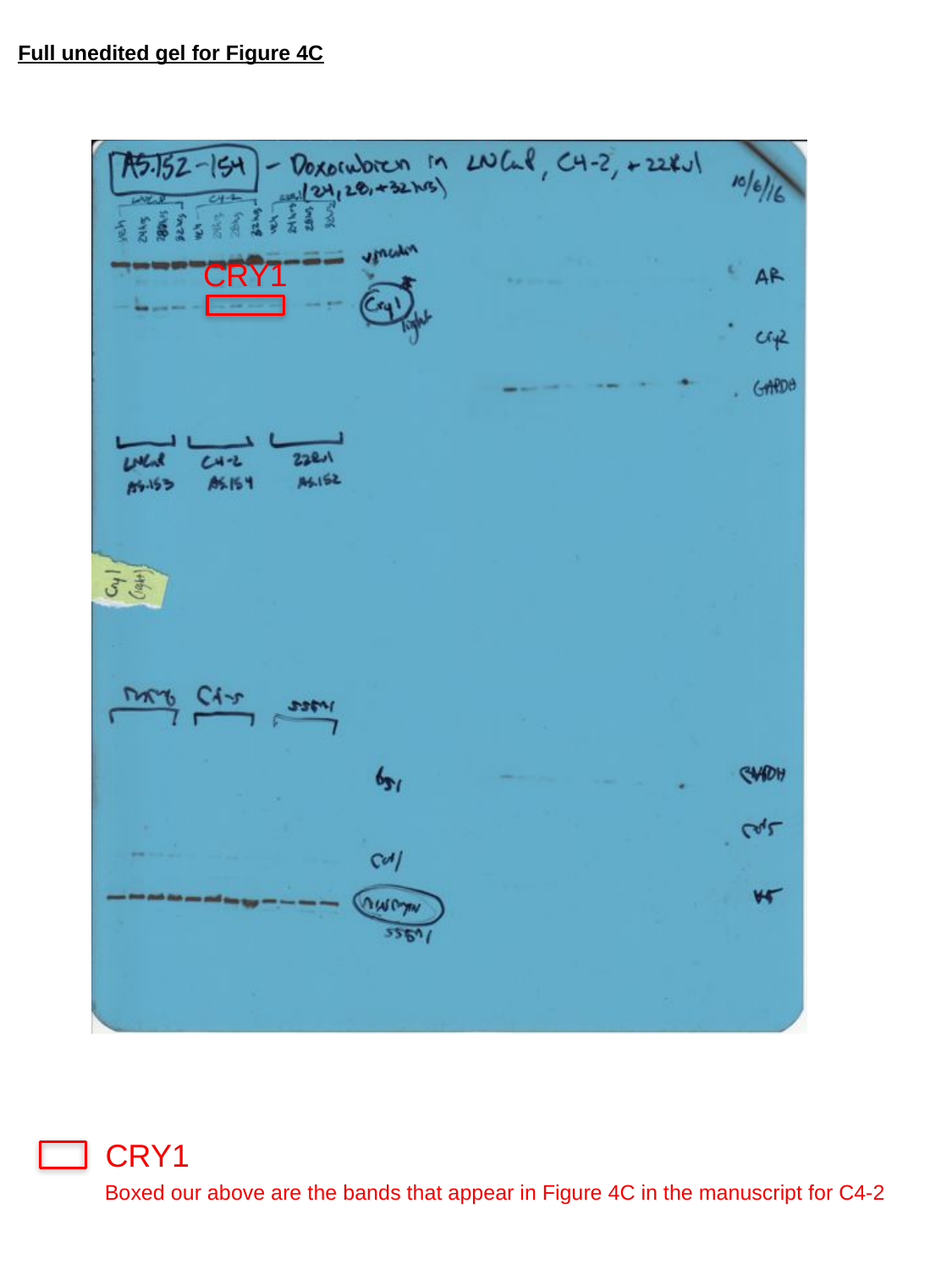

Full unedited gel for Figure 4C
CRY1
CRY1
Boxed our above are the bands that appear in Figure 4C in the manuscript for C4-2

## Slide 19
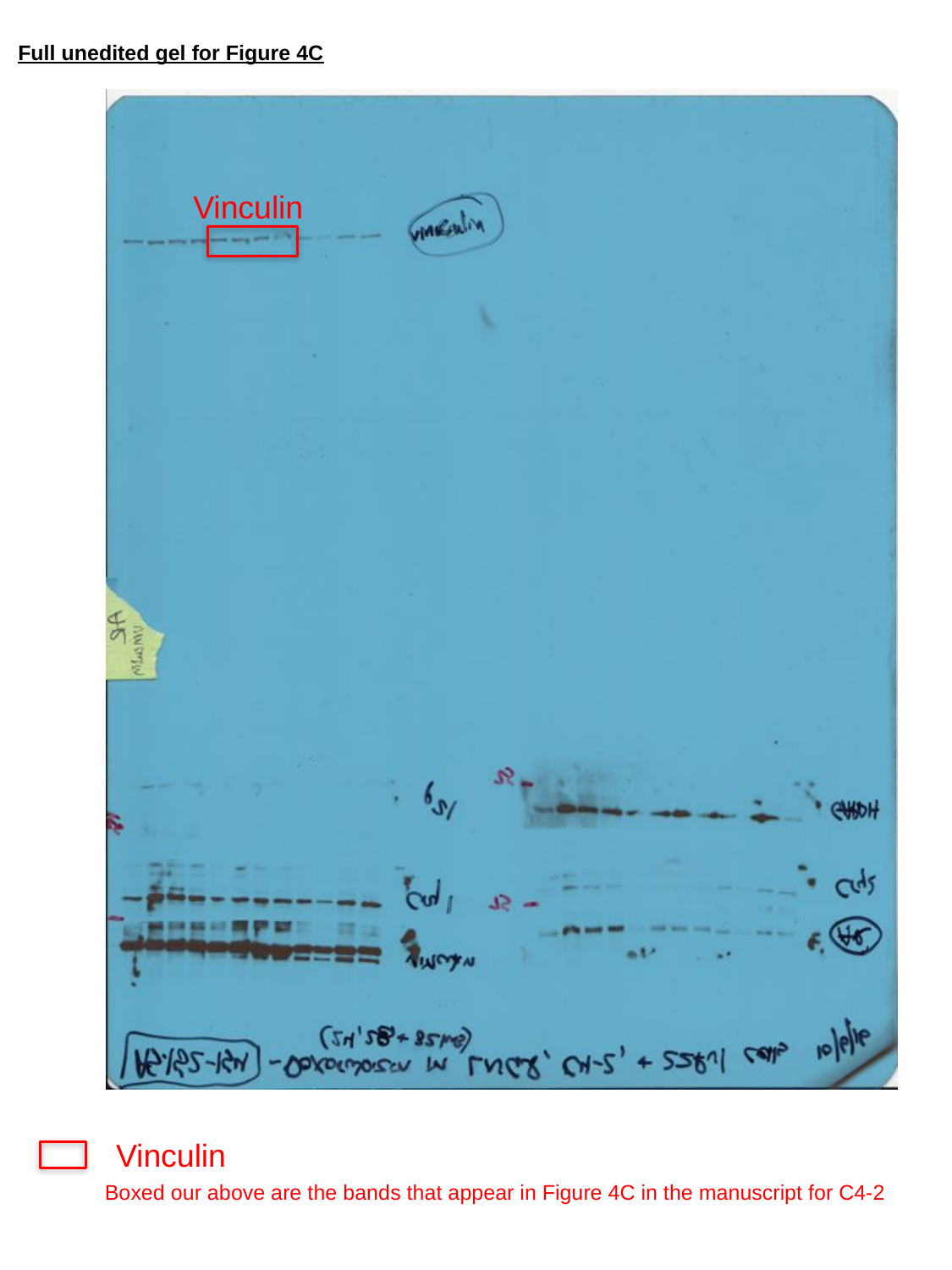

Full unedited gel for Figure 4C
Vinculin
Vinculin
Boxed our above are the bands that appear in Figure 4C in the manuscript for C4-2

## Slide 20
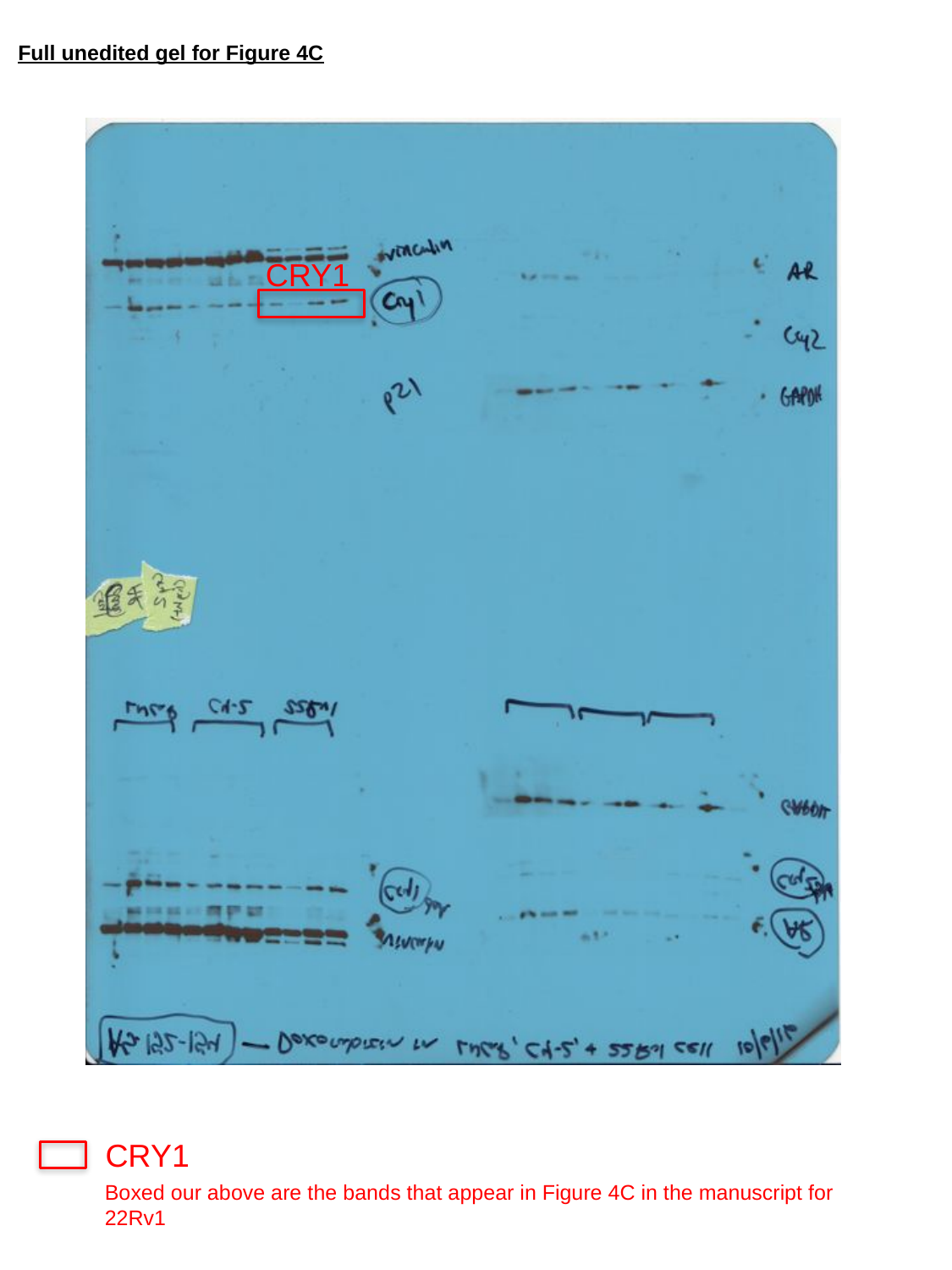

Full unedited gel for Figure 4C
CRY1
CRY1
Boxed our above are the bands that appear in Figure 4C in the manuscript for 22Rv1

## Slide 21
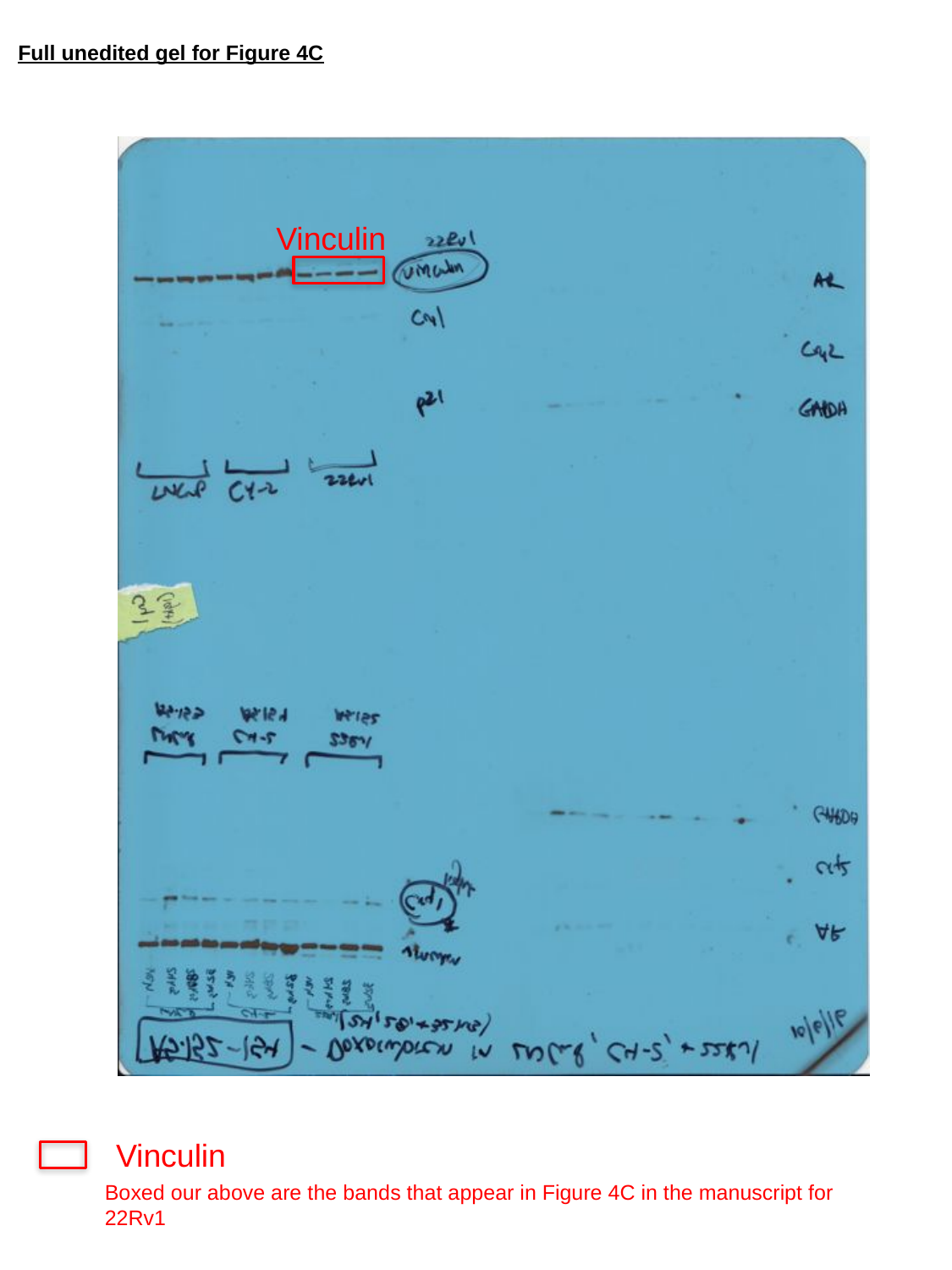

Full unedited gel for Figure 4C
Vinculin
Vinculin
Boxed our above are the bands that appear in Figure 4C in the manuscript for 22Rv1

## Slide 22
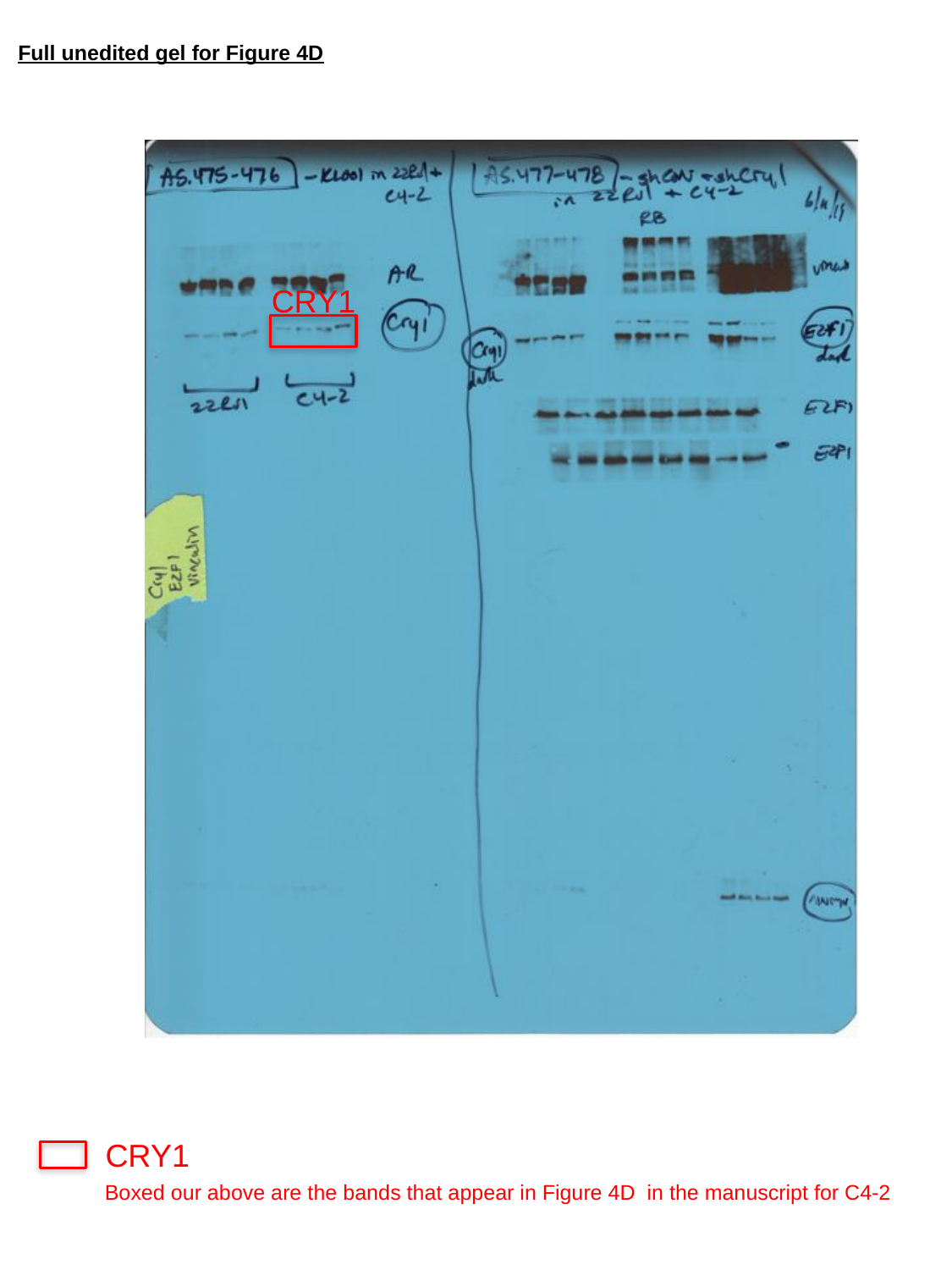

Full unedited gel for Figure 4D
CRY1
CRY1
Boxed our above are the bands that appear in Figure 4D in the manuscript for C4-2

## Slide 23
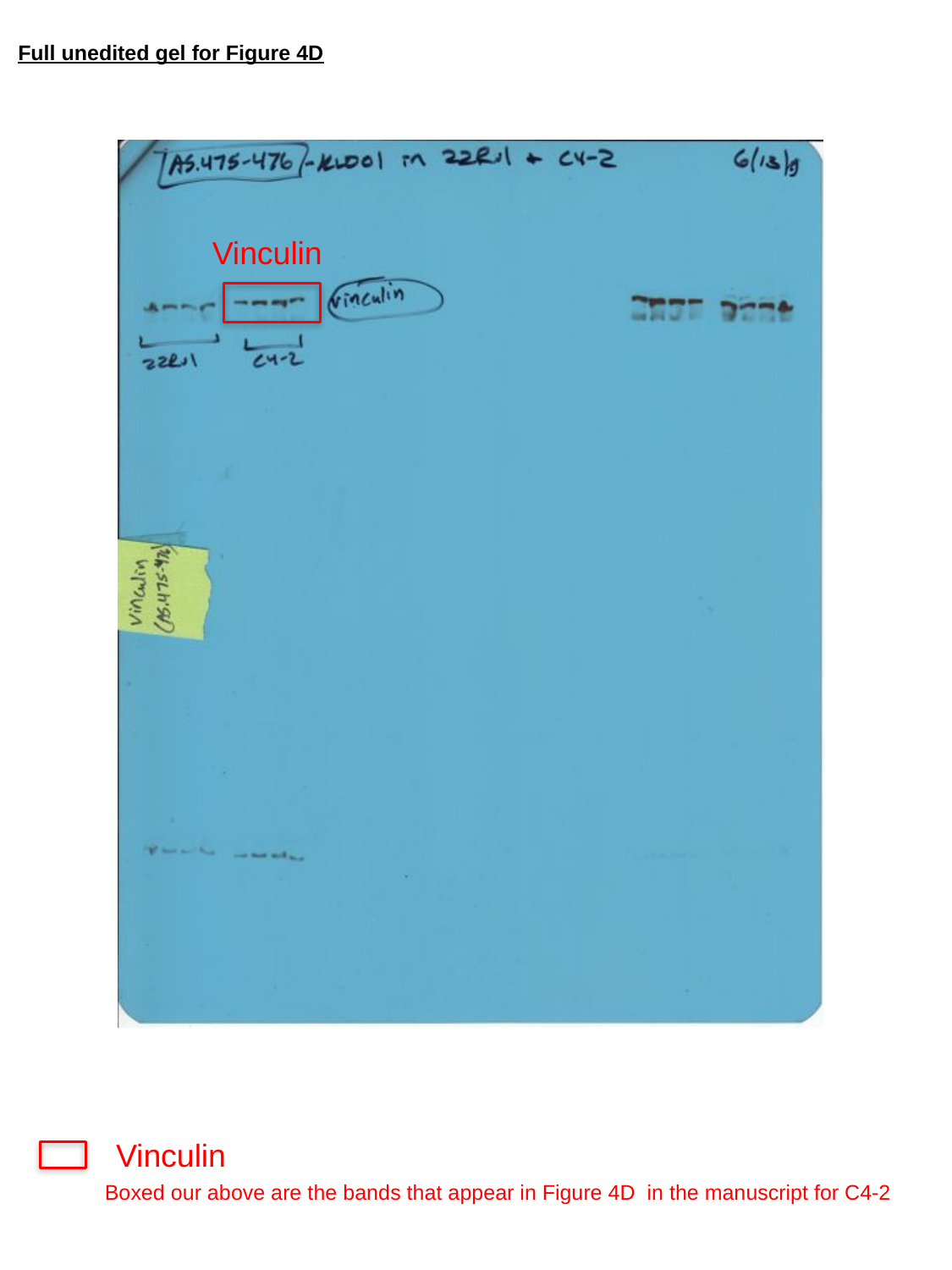

Full unedited gel for Figure 4D
Vinculin
Vinculin
Boxed our above are the bands that appear in Figure 4D in the manuscript for C4-2

## Slide 24
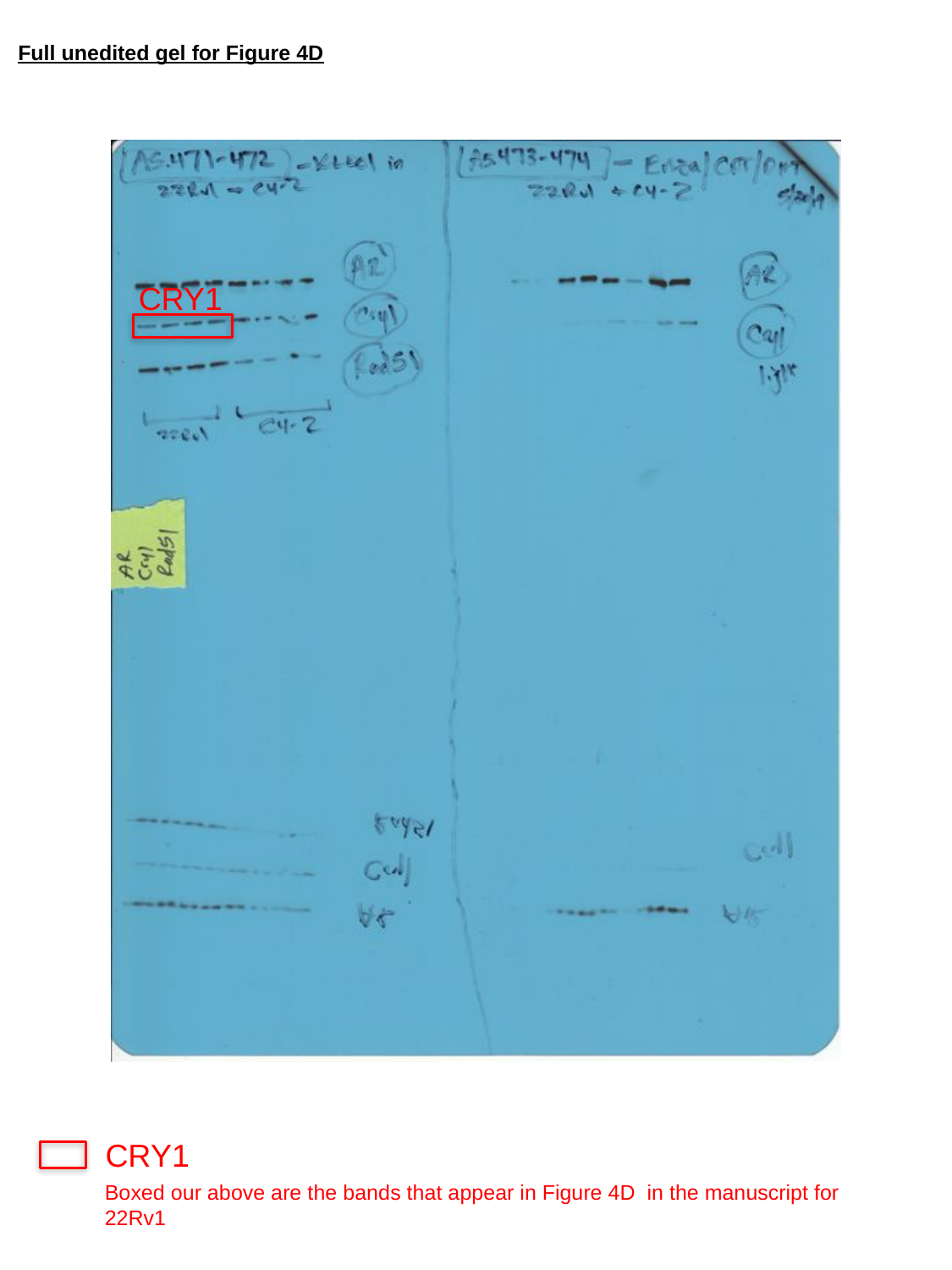

Full unedited gel for Figure 4D
CRY1
CRY1
Boxed our above are the bands that appear in Figure 4D in the manuscript for 22Rv1

## Slide 25
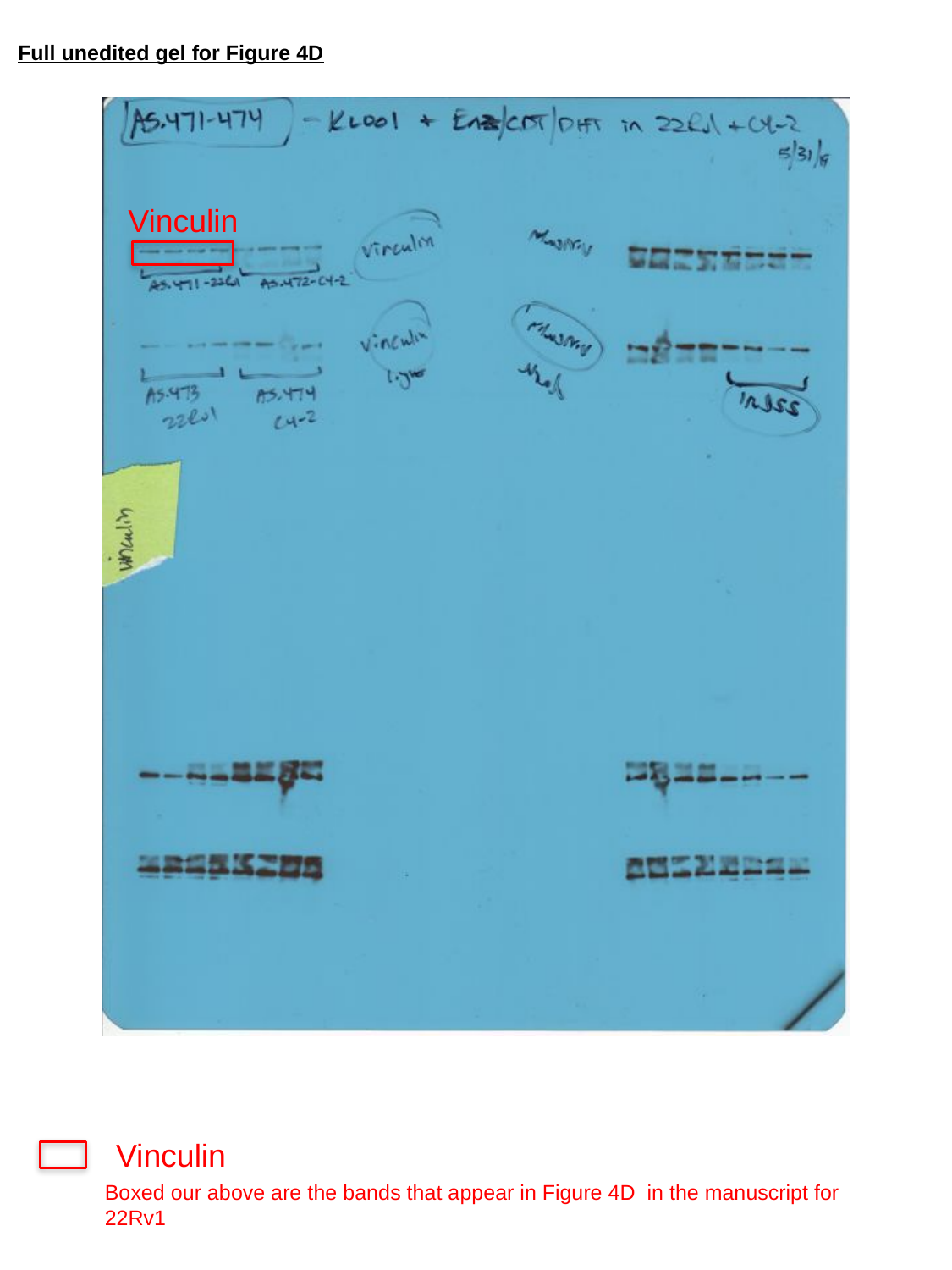

Full unedited gel for Figure 4D
Vinculin
Vinculin
Boxed our above are the bands that appear in Figure 4D in the manuscript for 22Rv1

## Slide 26
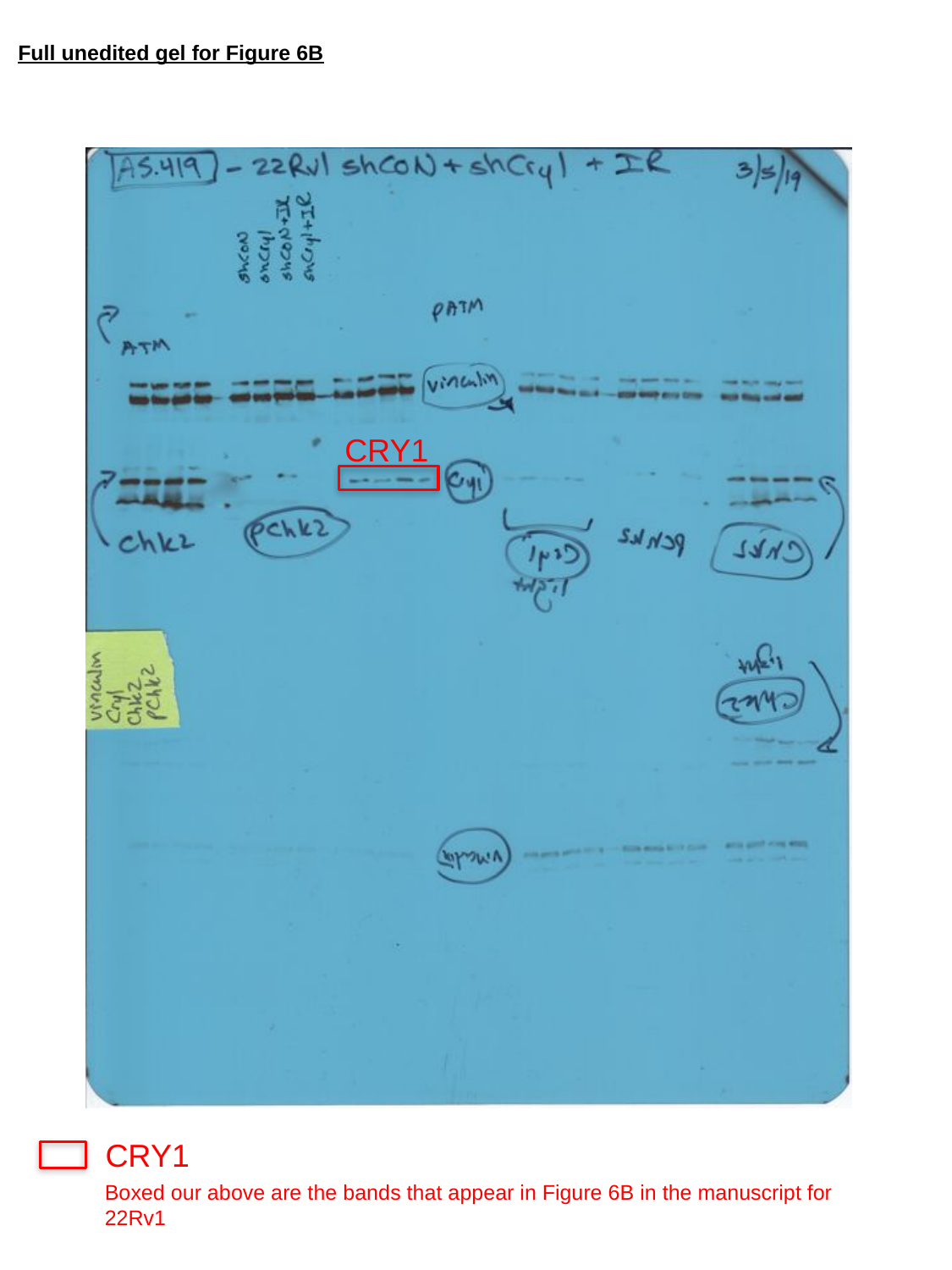

Full unedited gel for Figure 6B
CRY1
CRY1
Boxed our above are the bands that appear in Figure 6B in the manuscript for 22Rv1

## Slide 27
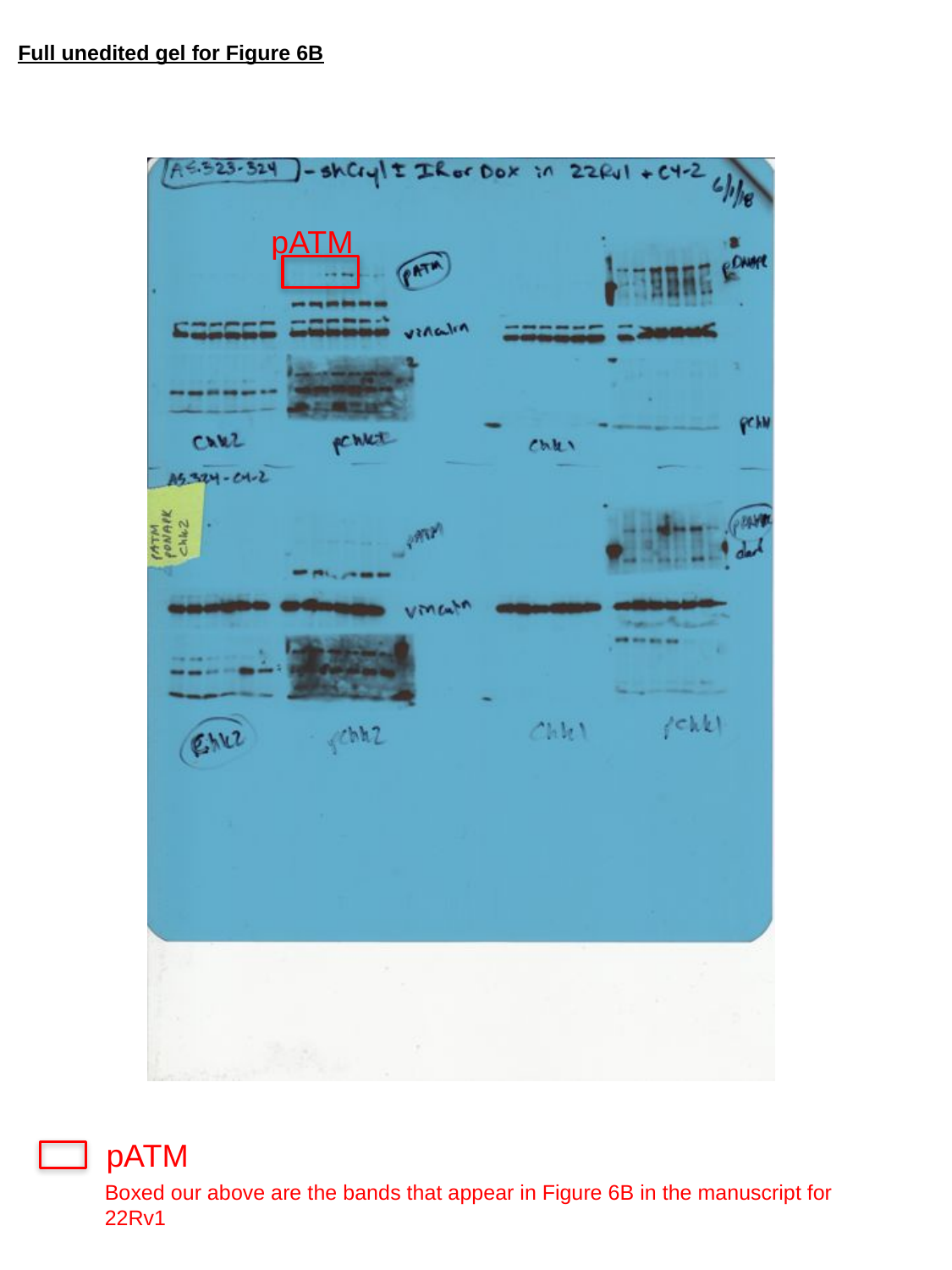

Full unedited gel for Figure 6B
pATM
pATM
Boxed our above are the bands that appear in Figure 6B in the manuscript for 22Rv1

## Slide 28
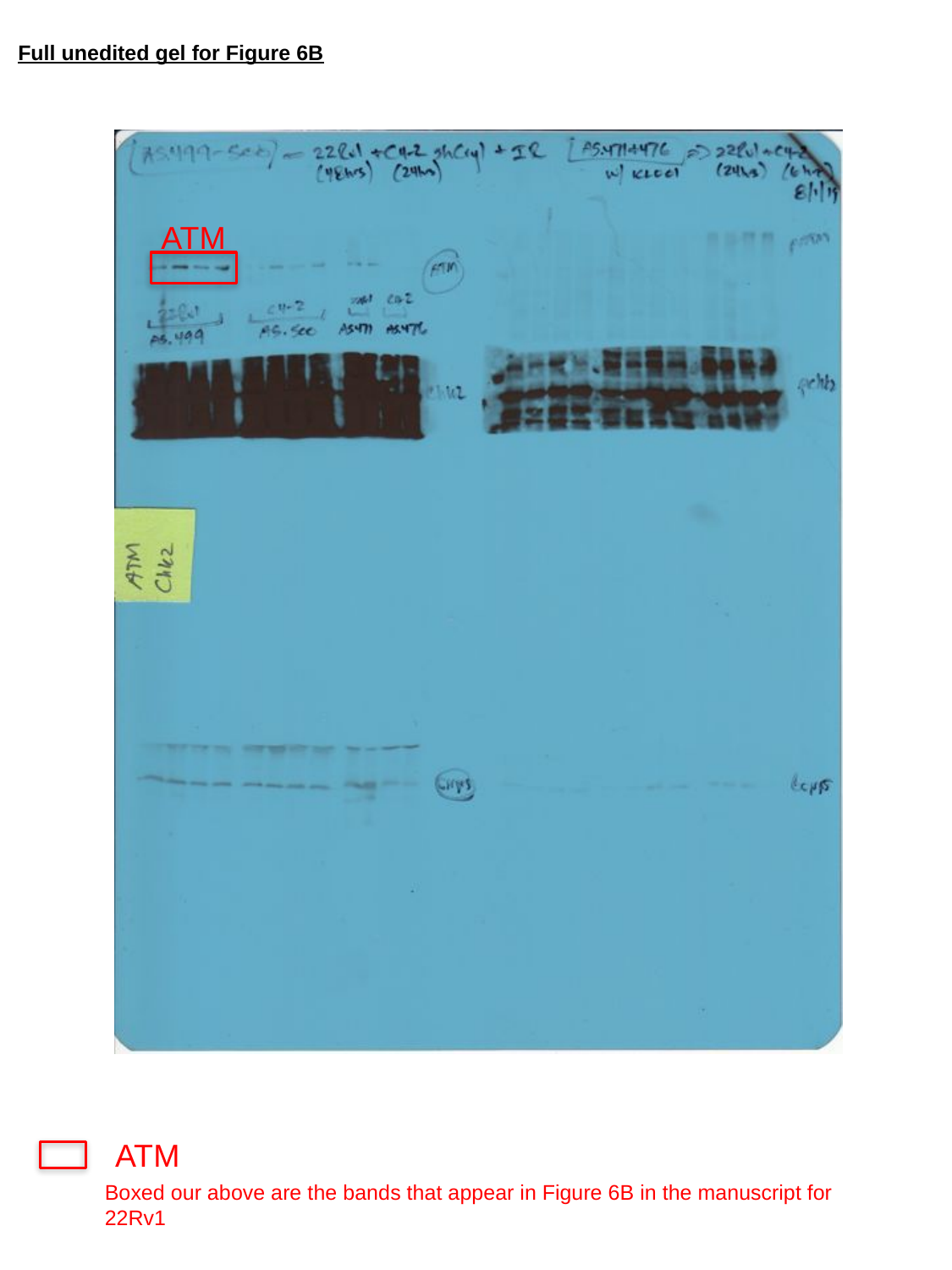

Full unedited gel for Figure 6B
ATM
ATM
Boxed our above are the bands that appear in Figure 6B in the manuscript for 22Rv1

## Slide 29
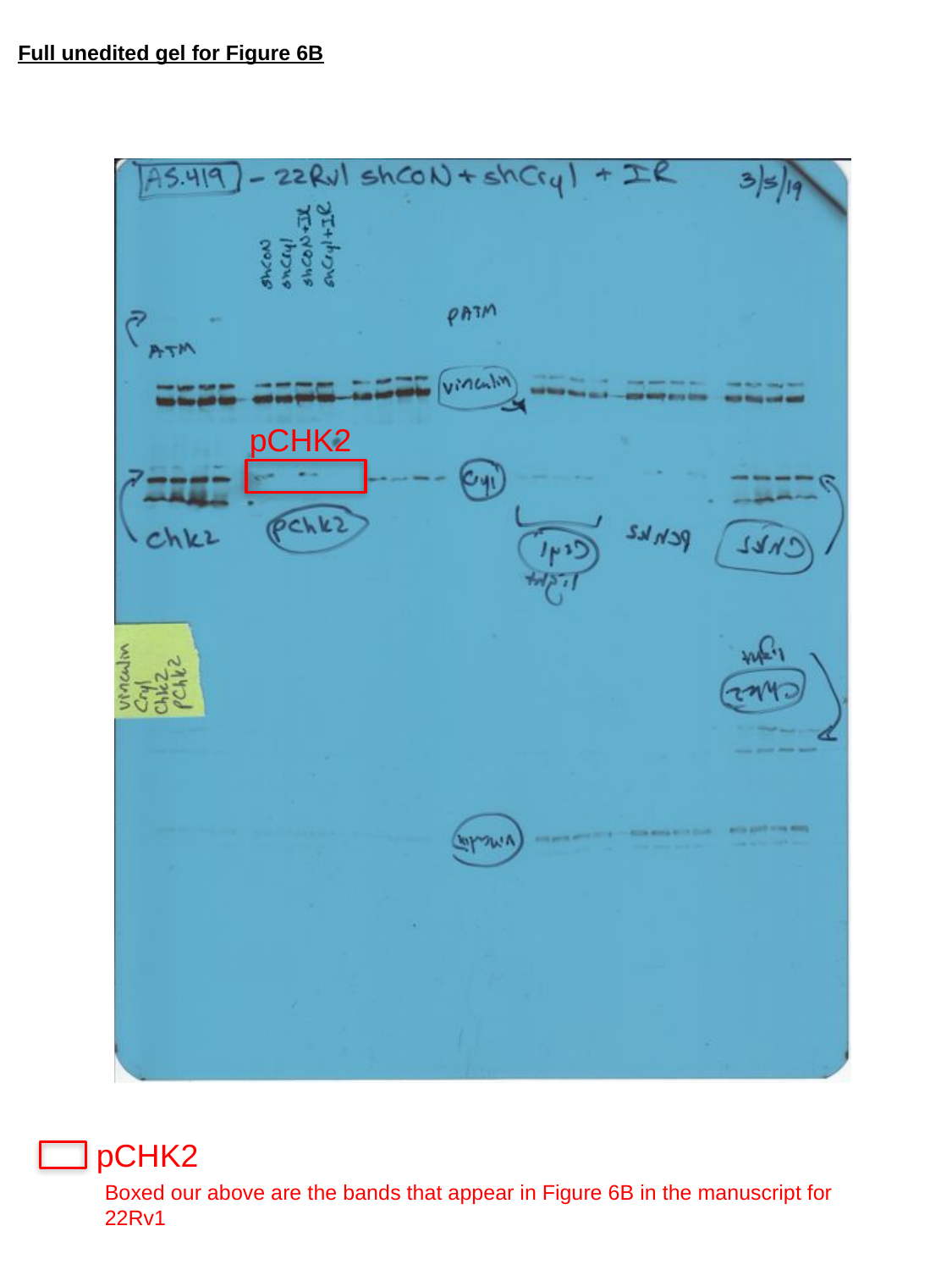

Full unedited gel for Figure 6B
pCHK2
pCHK2
Boxed our above are the bands that appear in Figure 6B in the manuscript for 22Rv1

## Slide 30
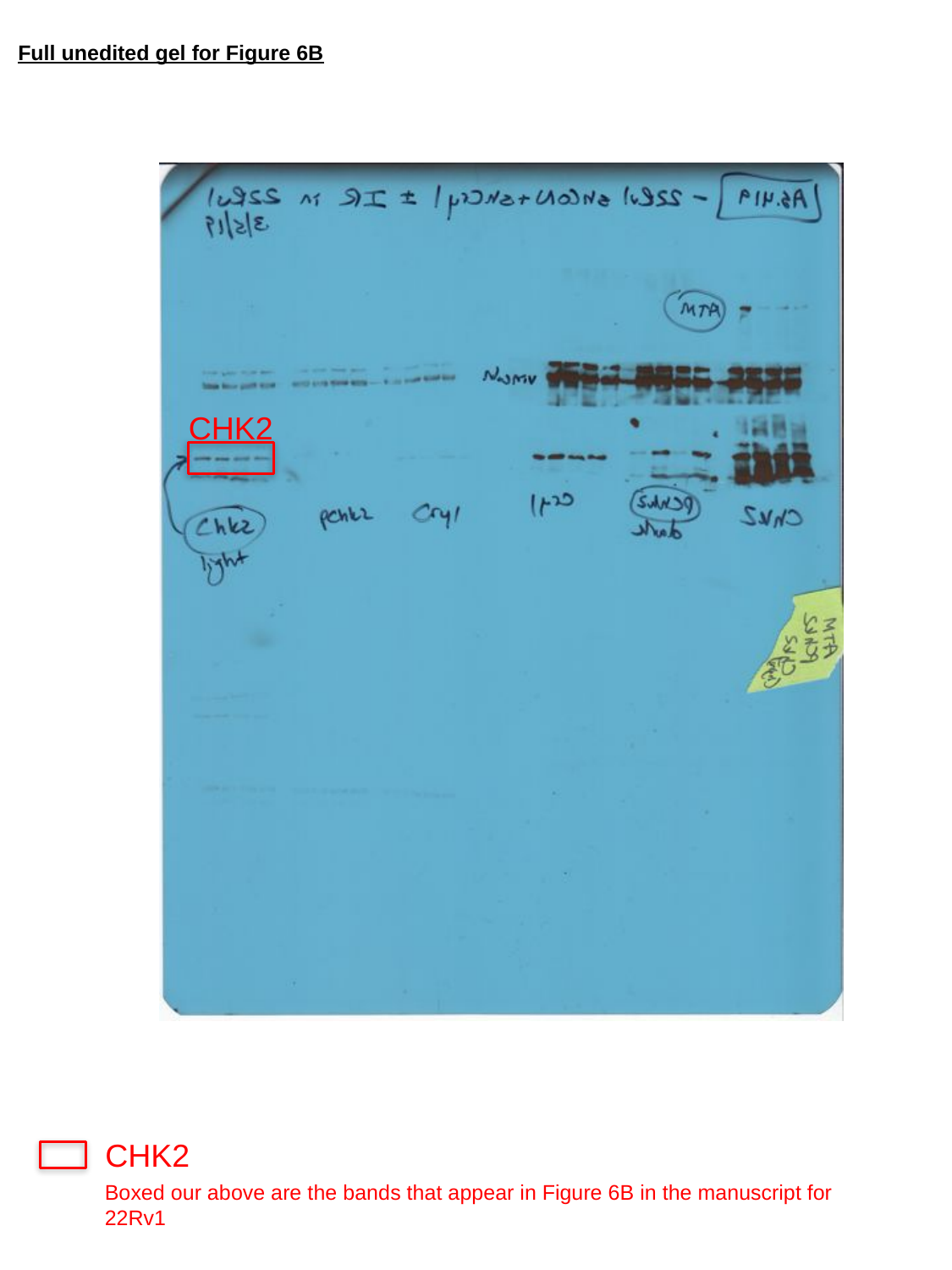

Full unedited gel for Figure 6B
CHK2
CHK2
Boxed our above are the bands that appear in Figure 6B in the manuscript for 22Rv1

## Slide 31
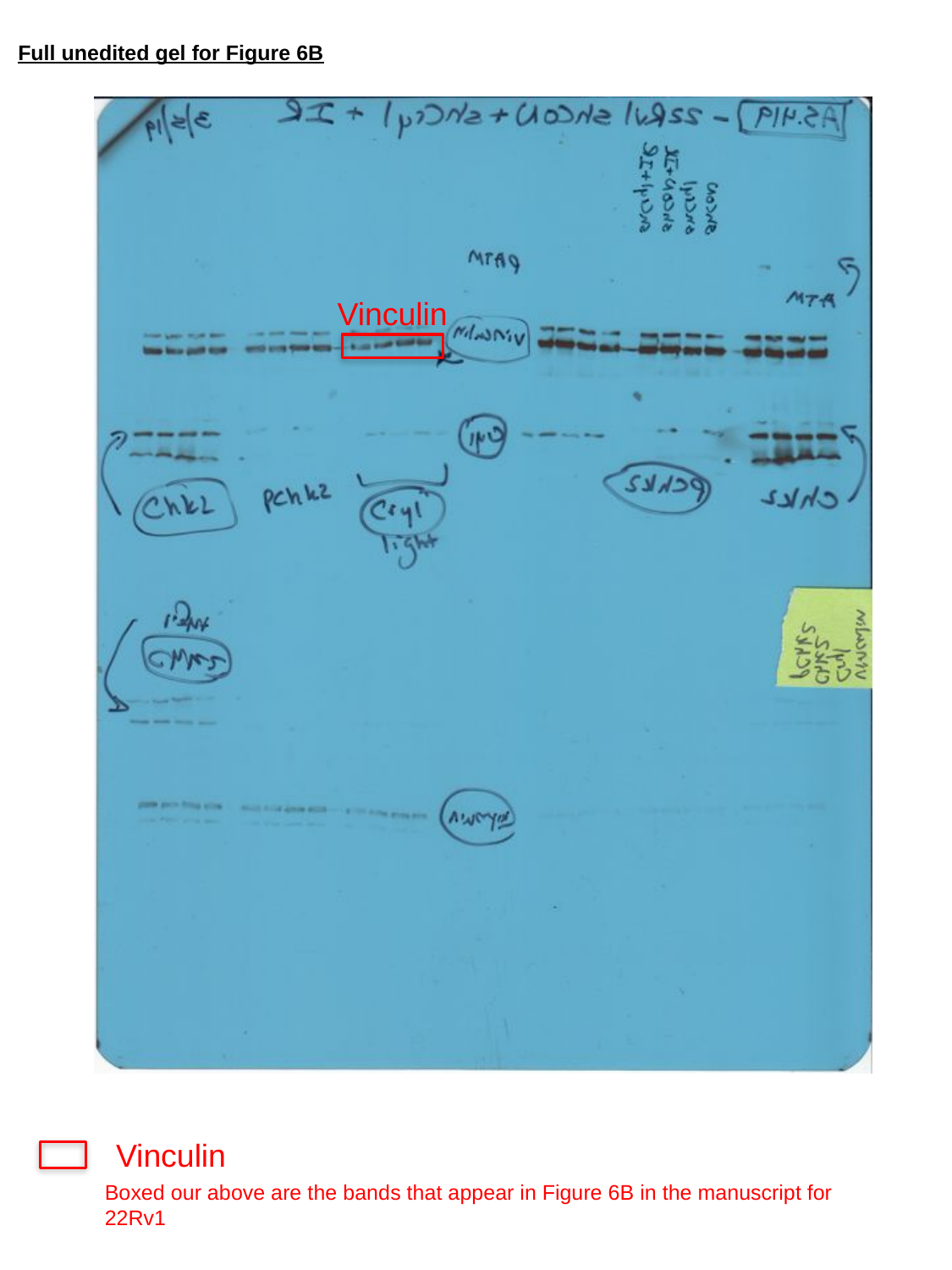

Full unedited gel for Figure 6B
Vinculin
Vinculin
Boxed our above are the bands that appear in Figure 6B in the manuscript for 22Rv1

## Slide 32
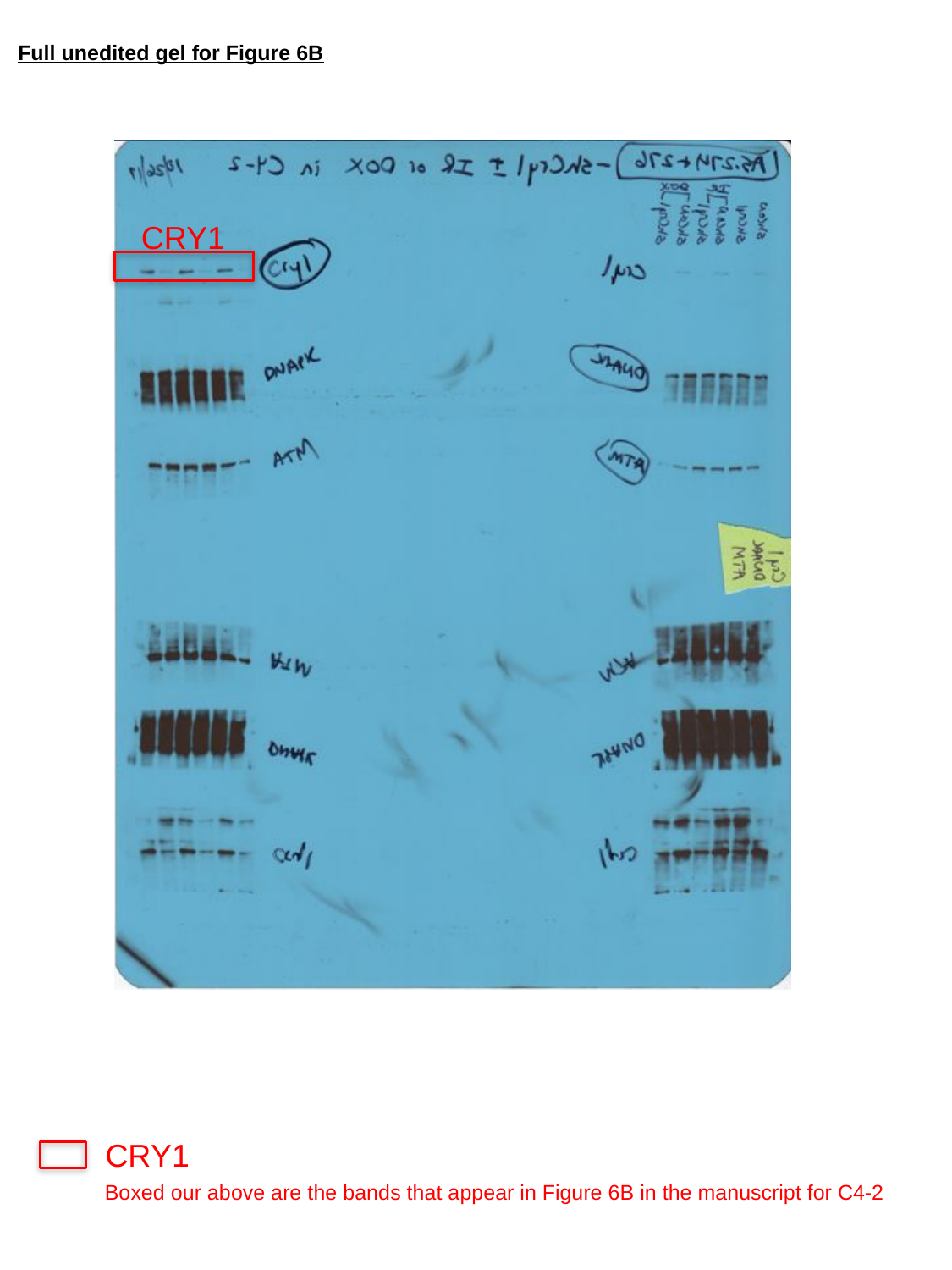

Full unedited gel for Figure 6B
CRY1
CRY1
Boxed our above are the bands that appear in Figure 6B in the manuscript for C4-2

## Slide 33
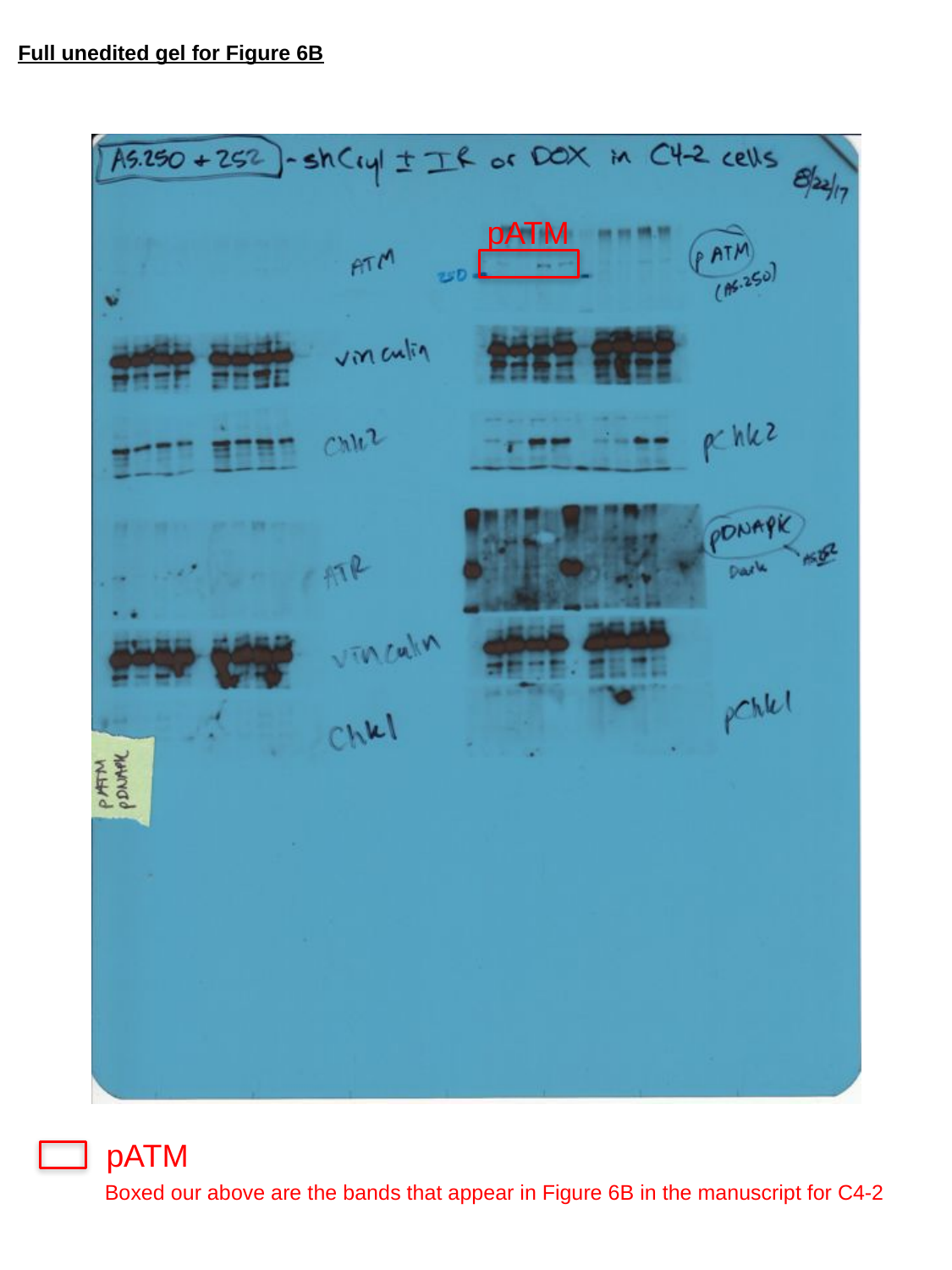

Full unedited gel for Figure 6B
pATM
pATM
Boxed our above are the bands that appear in Figure 6B in the manuscript for C4-2

## Slide 34
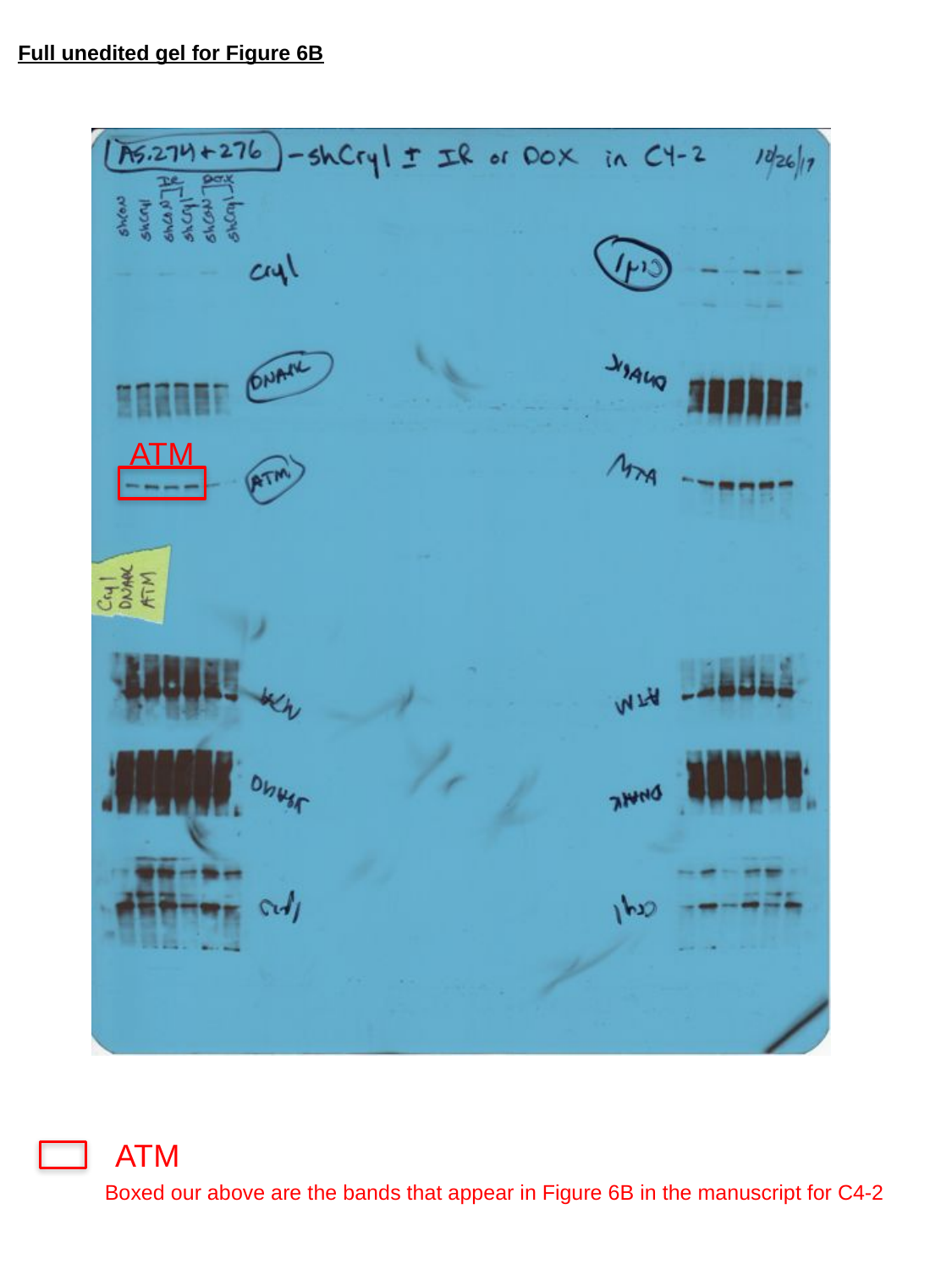

Full unedited gel for Figure 6B
ATM
ATM
Boxed our above are the bands that appear in Figure 6B in the manuscript for C4-2

## Slide 35
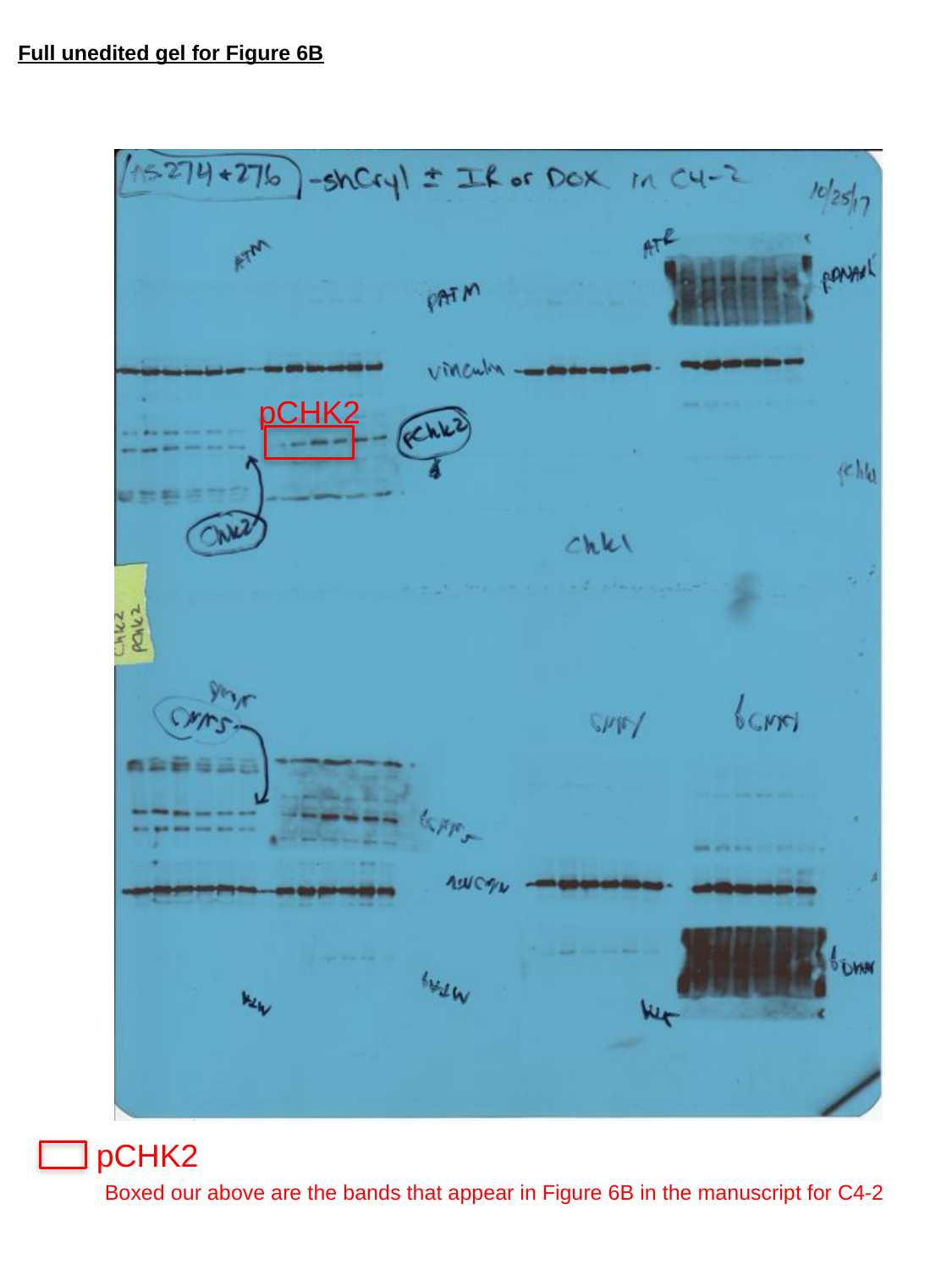

Full unedited gel for Figure 6B
pCHK2
pCHK2
Boxed our above are the bands that appear in Figure 6B in the manuscript for C4-2

## Slide 36
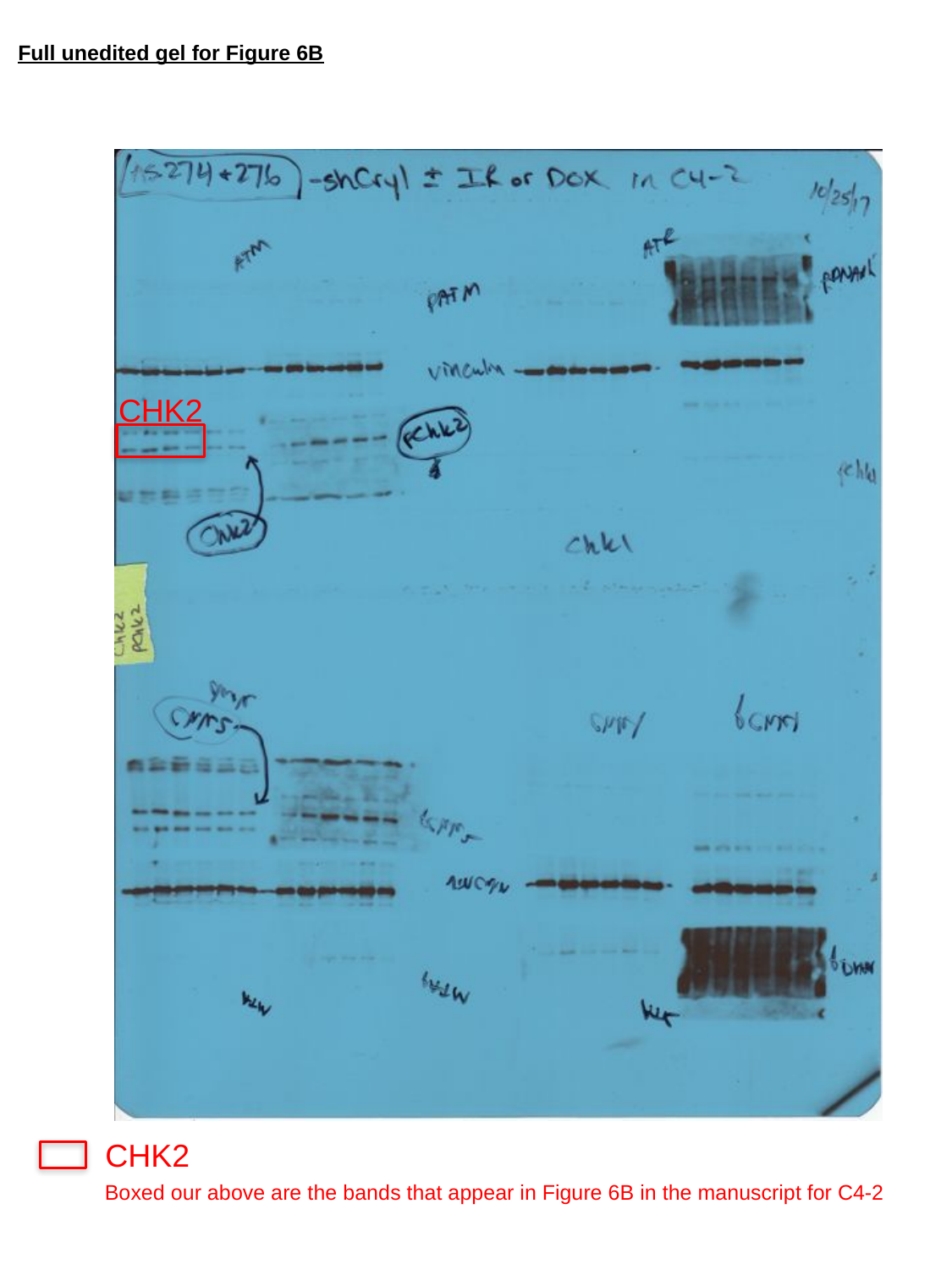

Full unedited gel for Figure 6B
CHK2
CHK2
Boxed our above are the bands that appear in Figure 6B in the manuscript for C4-2

## Slide 37
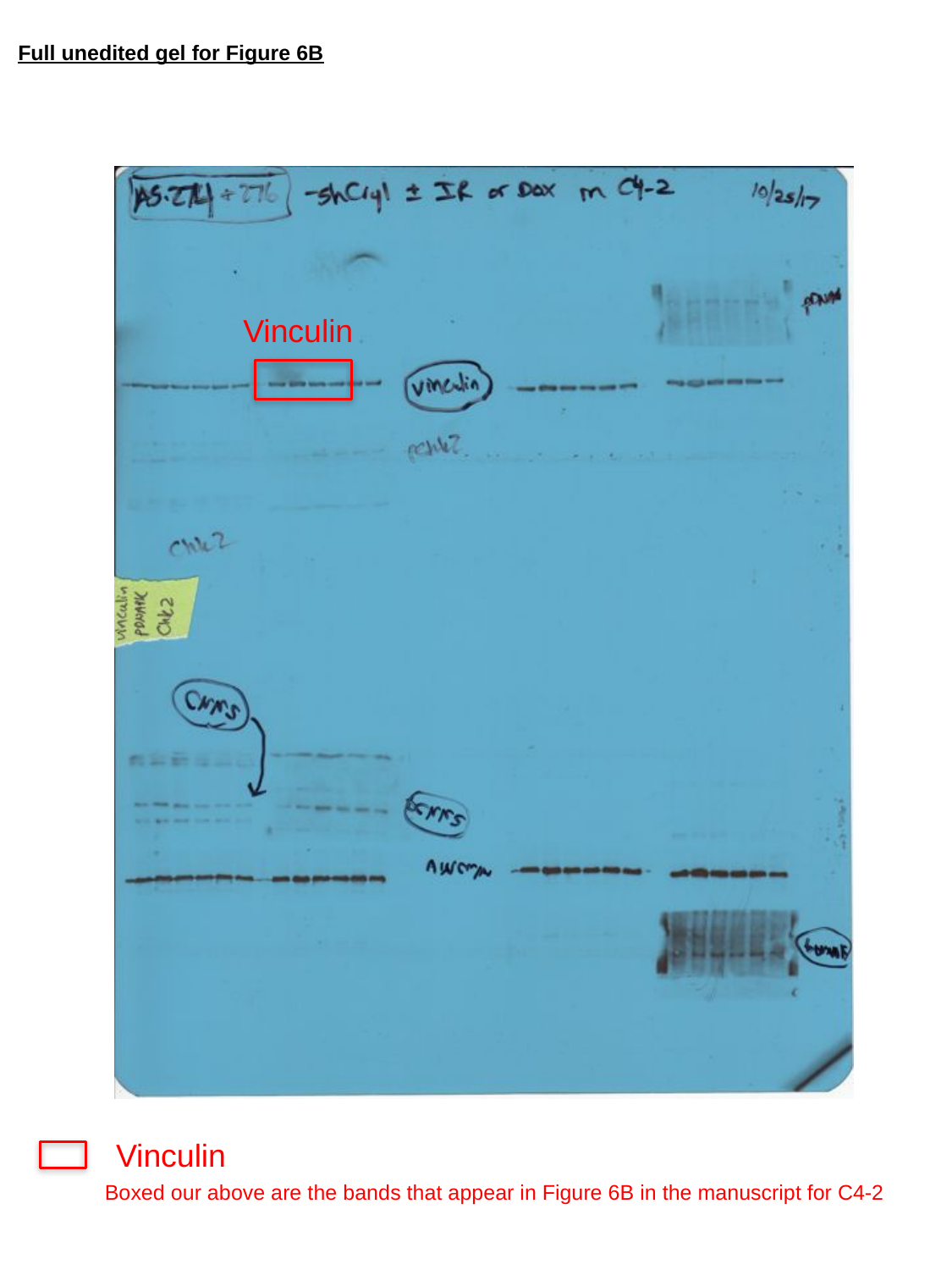

Full unedited gel for Figure 6B
Vinculin
Vinculin
Boxed our above are the bands that appear in Figure 6B in the manuscript for C4-2

## Slide 38
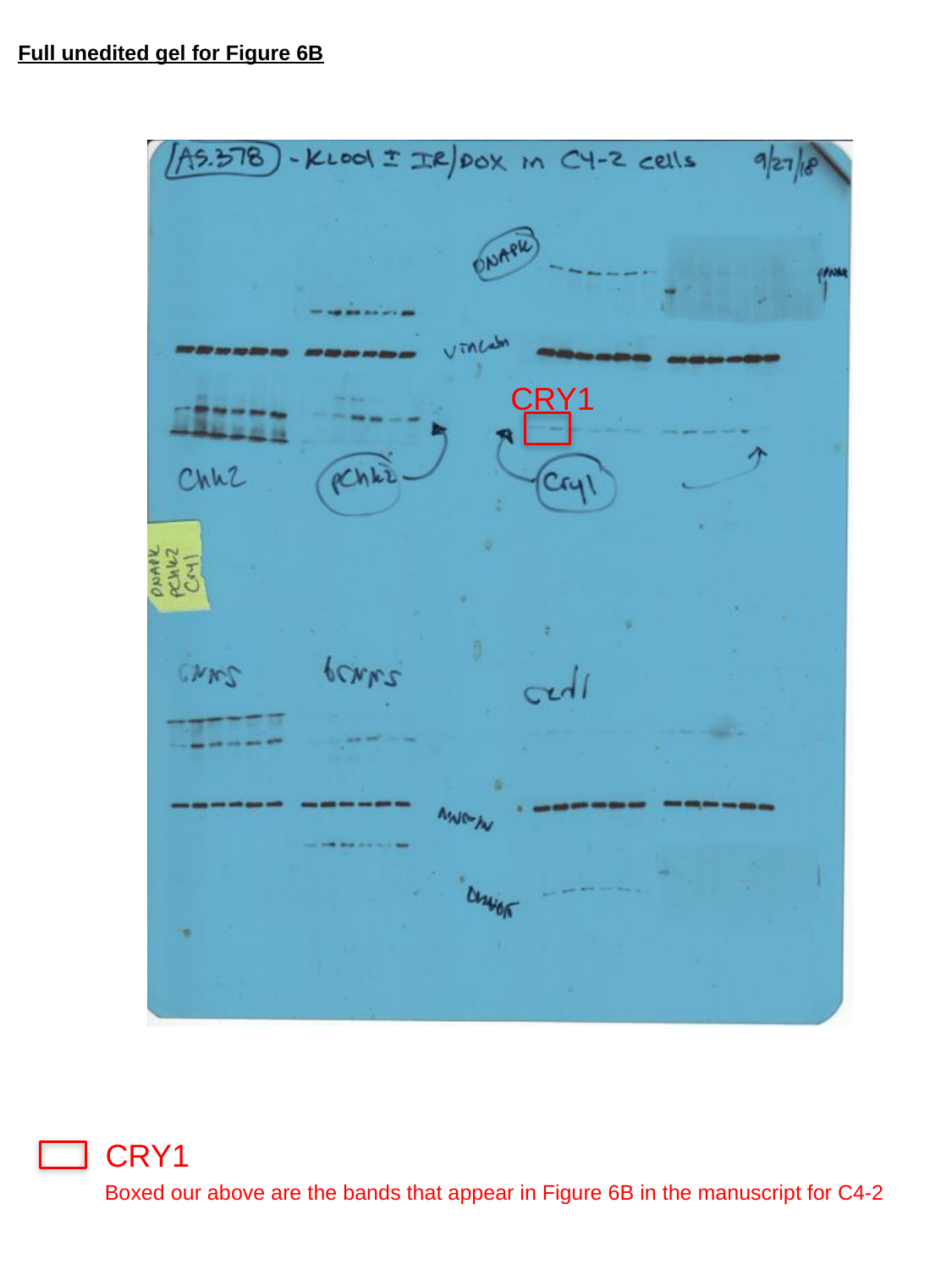

Full unedited gel for Figure 6B
CRY1
CRY1
Boxed our above are the bands that appear in Figure 6B in the manuscript for C4-2

## Slide 39
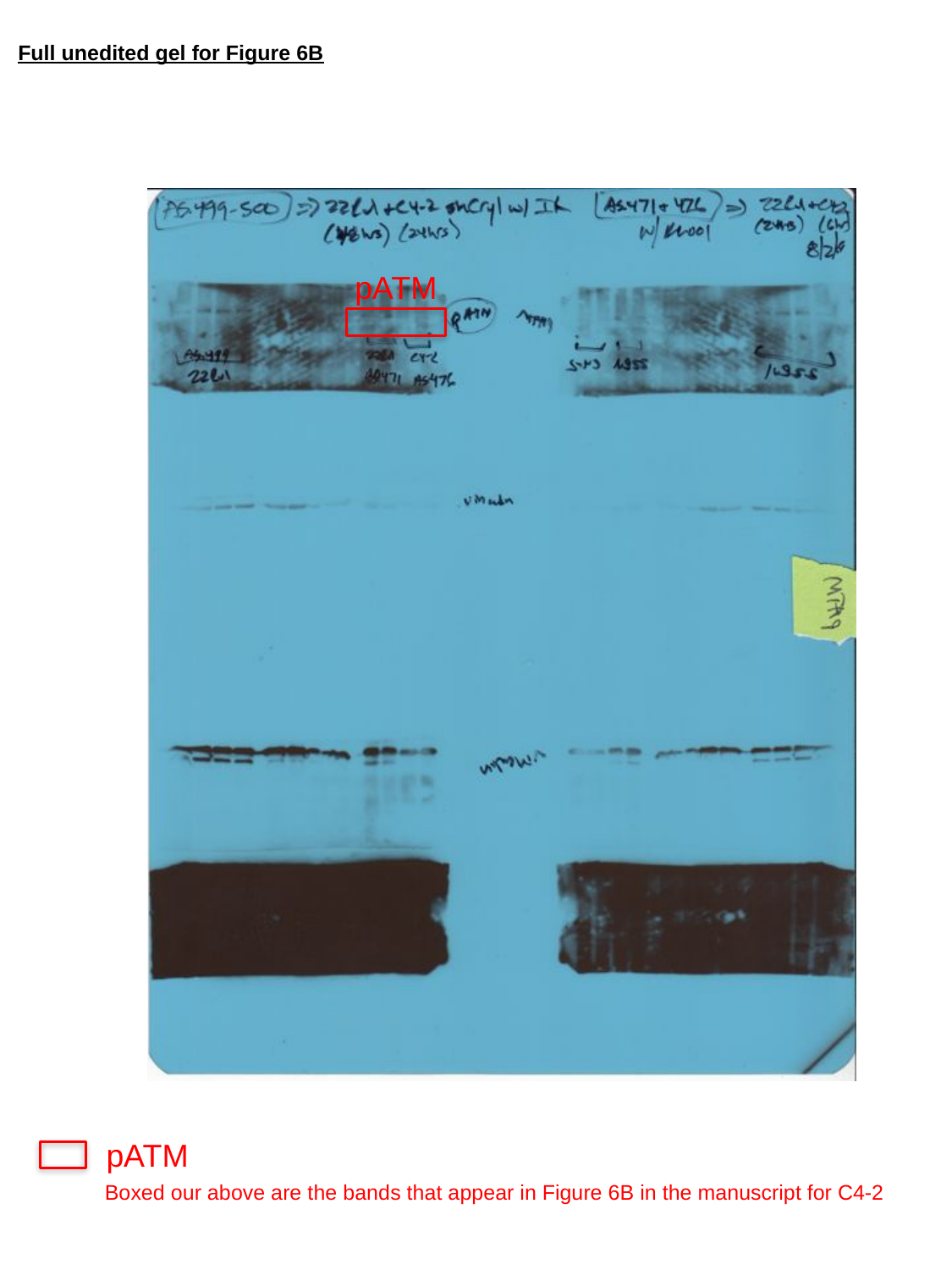

Full unedited gel for Figure 6B
pATM
pATM
Boxed our above are the bands that appear in Figure 6B in the manuscript for C4-2

## Slide 40
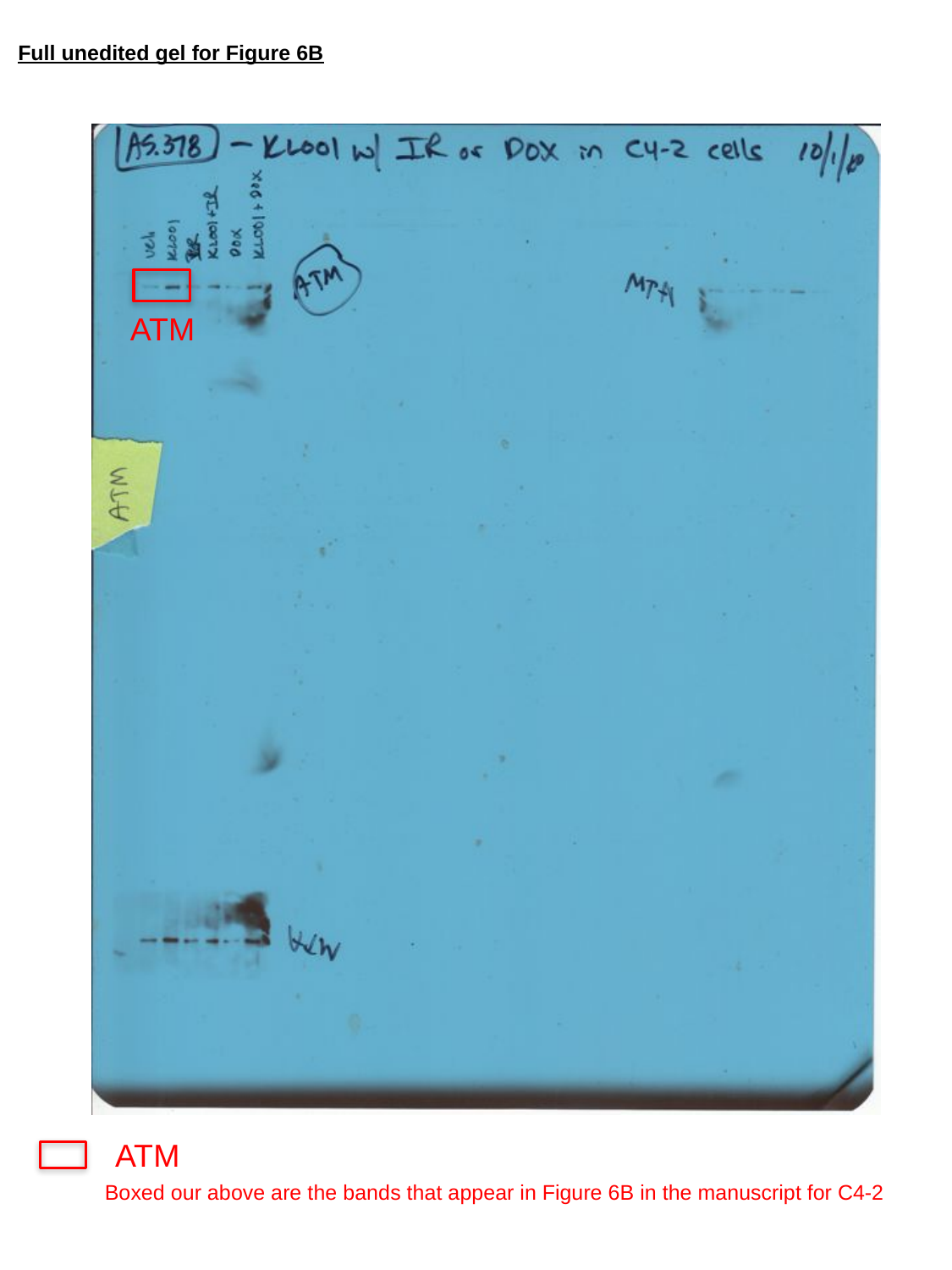

Full unedited gel for Figure 6B
ATM
ATM
Boxed our above are the bands that appear in Figure 6B in the manuscript for C4-2

## Slide 41
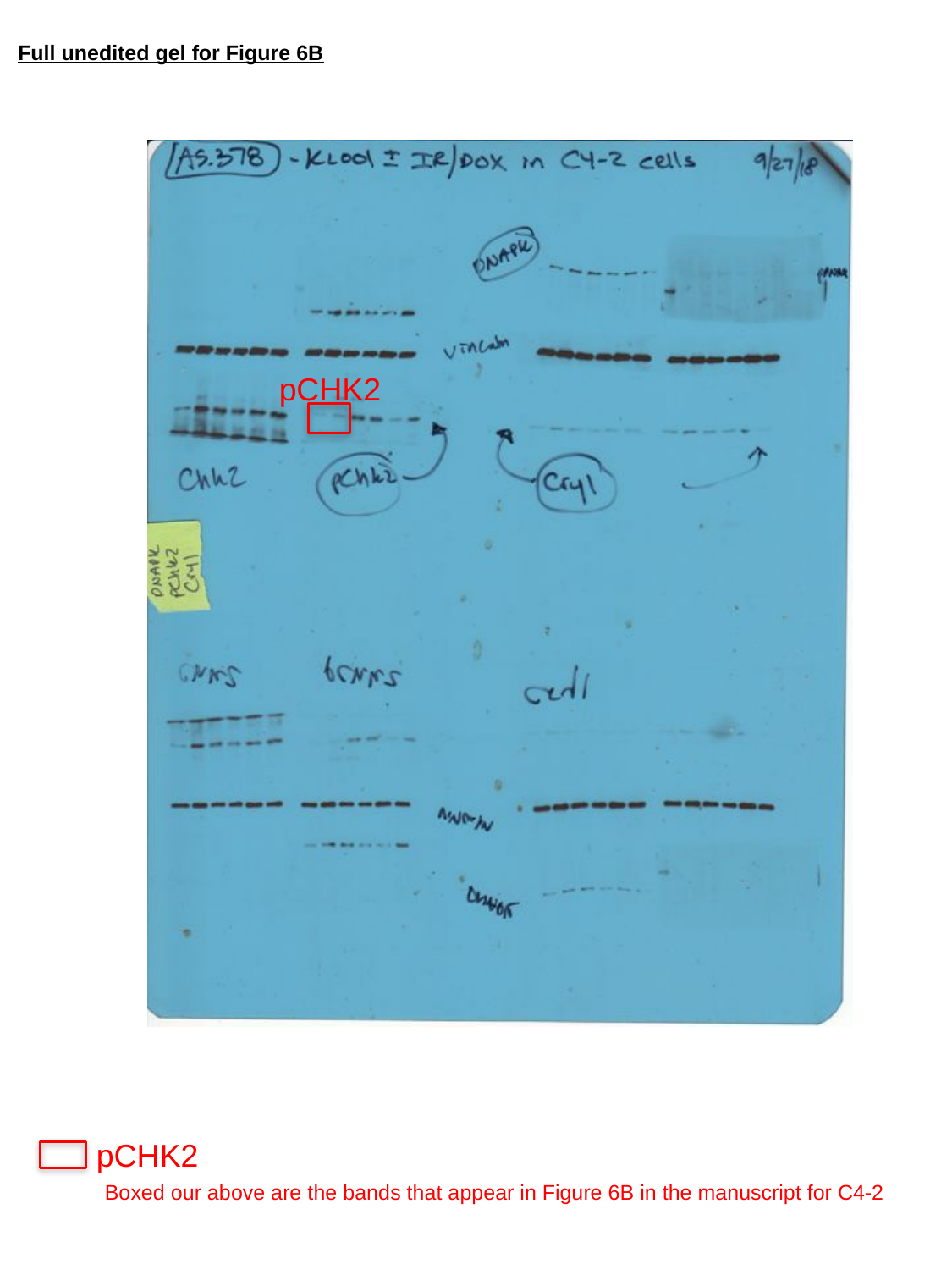

Full unedited gel for Figure 6B
pCHK2
pCHK2
Boxed our above are the bands that appear in Figure 6B in the manuscript for C4-2

## Slide 42
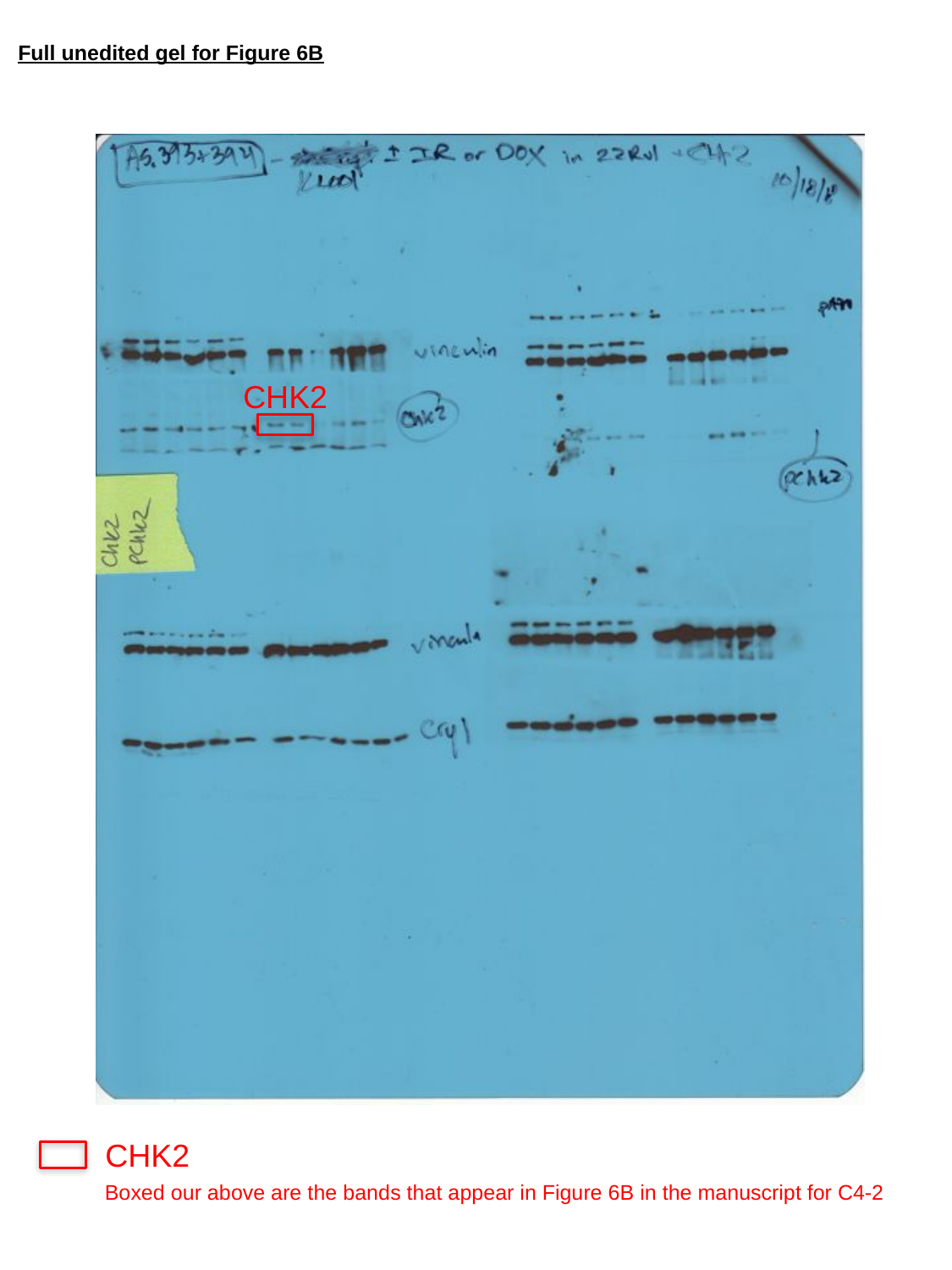

Full unedited gel for Figure 6B
CHK2
CHK2
Boxed our above are the bands that appear in Figure 6B in the manuscript for C4-2

## Slide 43
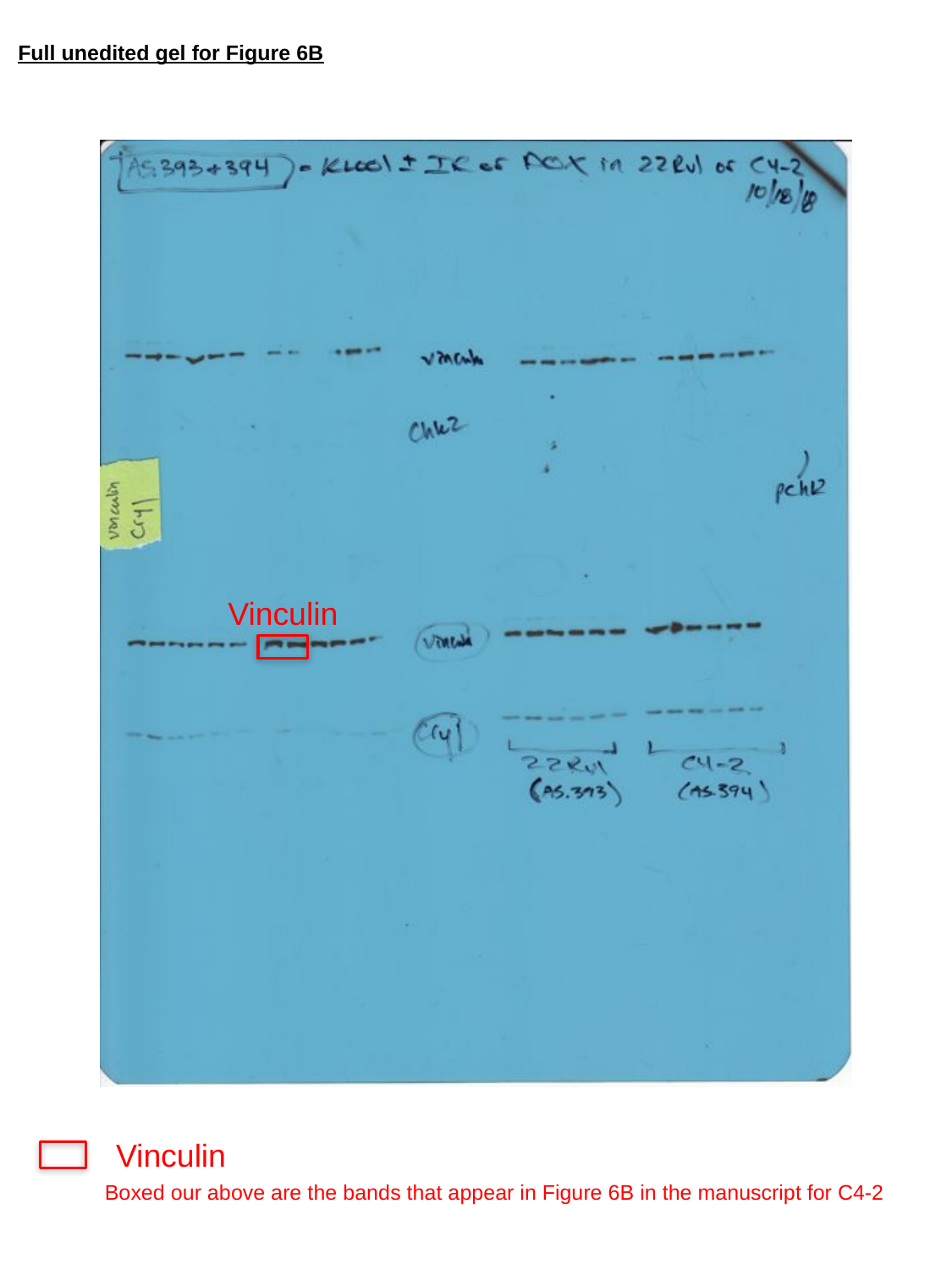

Full unedited gel for Figure 6B
Vinculin
Vinculin
Boxed our above are the bands that appear in Figure 6B in the manuscript for C4-2

## Slide 44
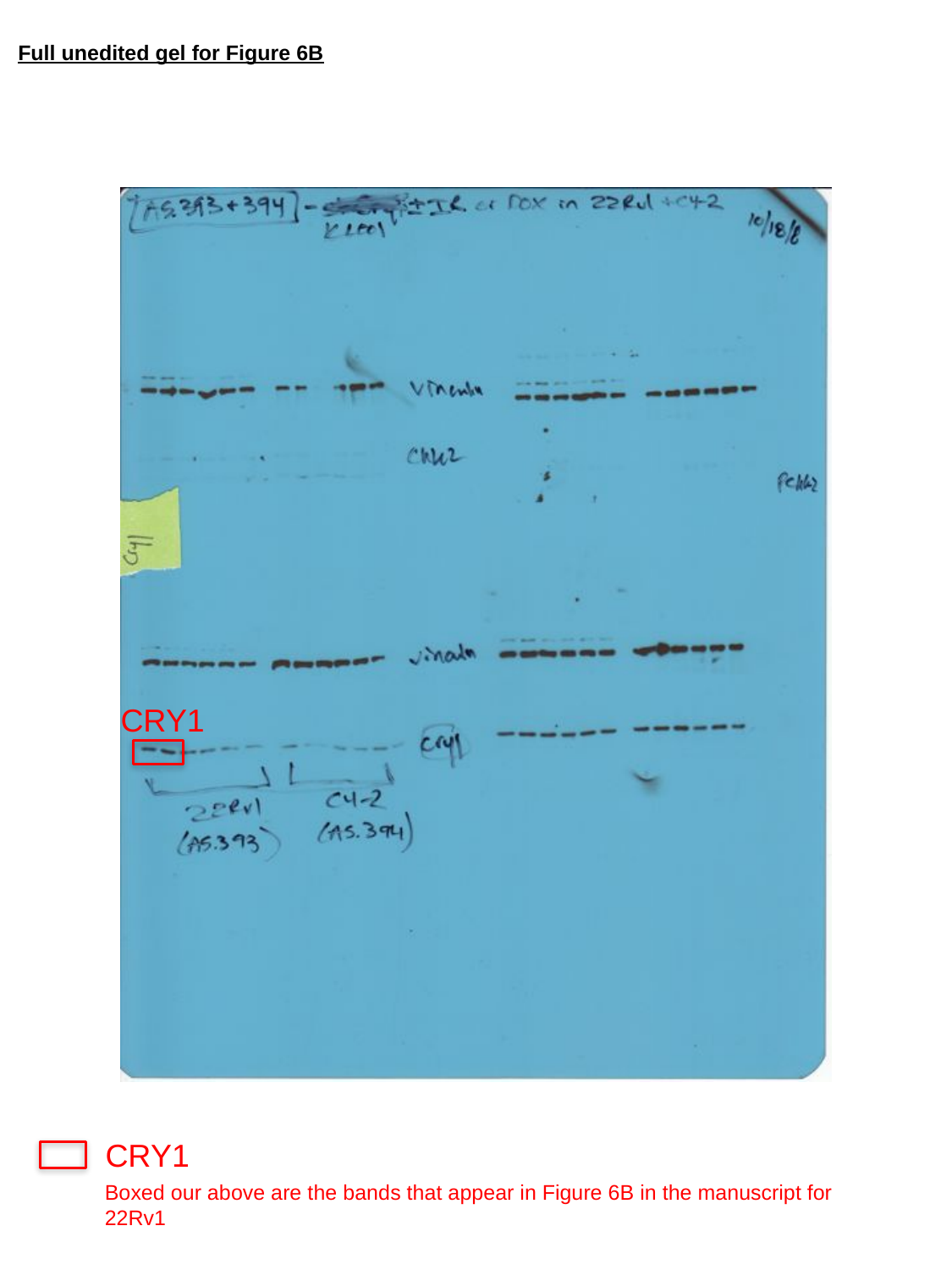

Full unedited gel for Figure 6B
CRY1
CRY1
Boxed our above are the bands that appear in Figure 6B in the manuscript for 22Rv1

## Slide 45
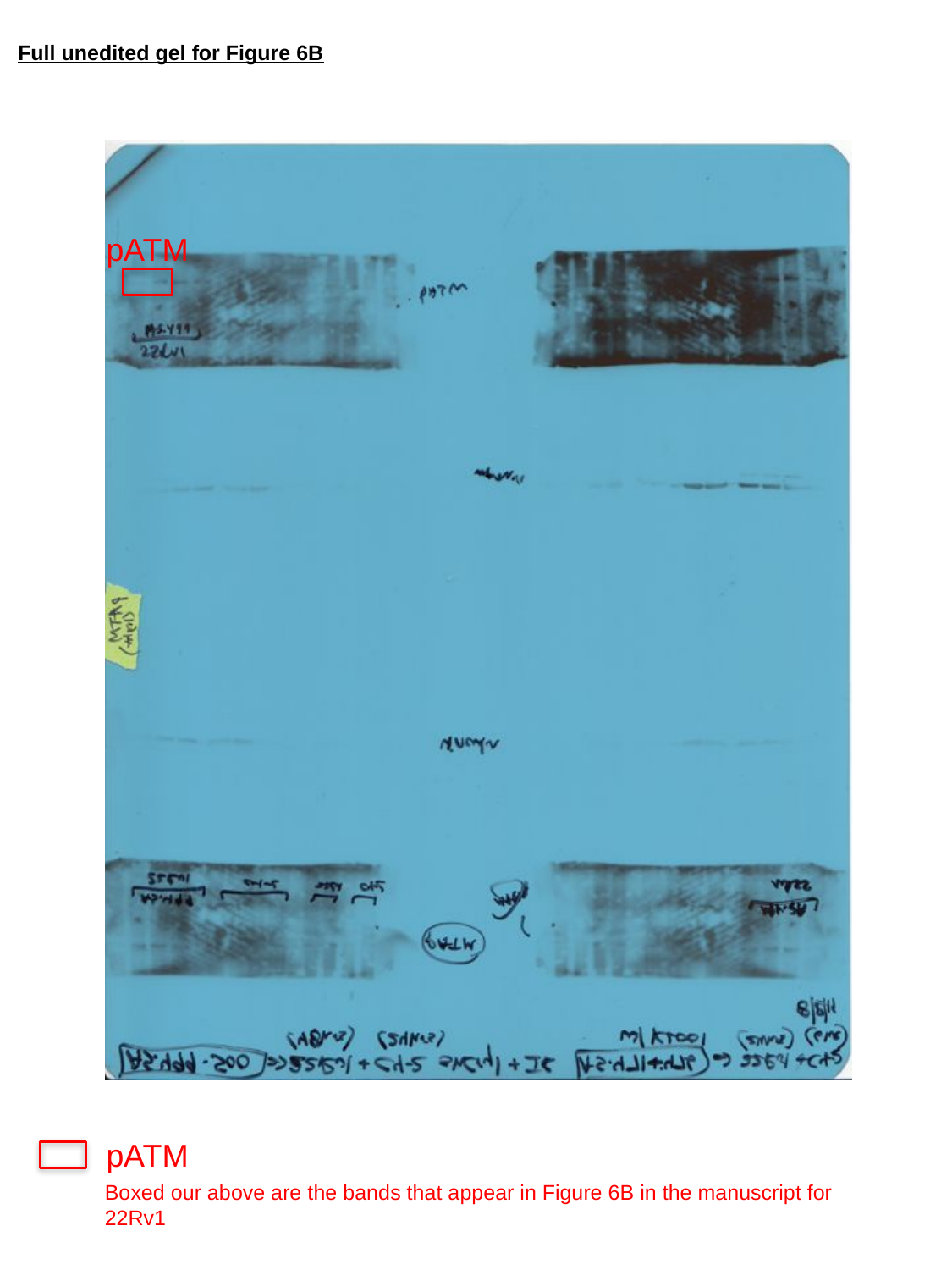

Full unedited gel for Figure 6B
pATM
pATM
Boxed our above are the bands that appear in Figure 6B in the manuscript for 22Rv1

## Slide 46
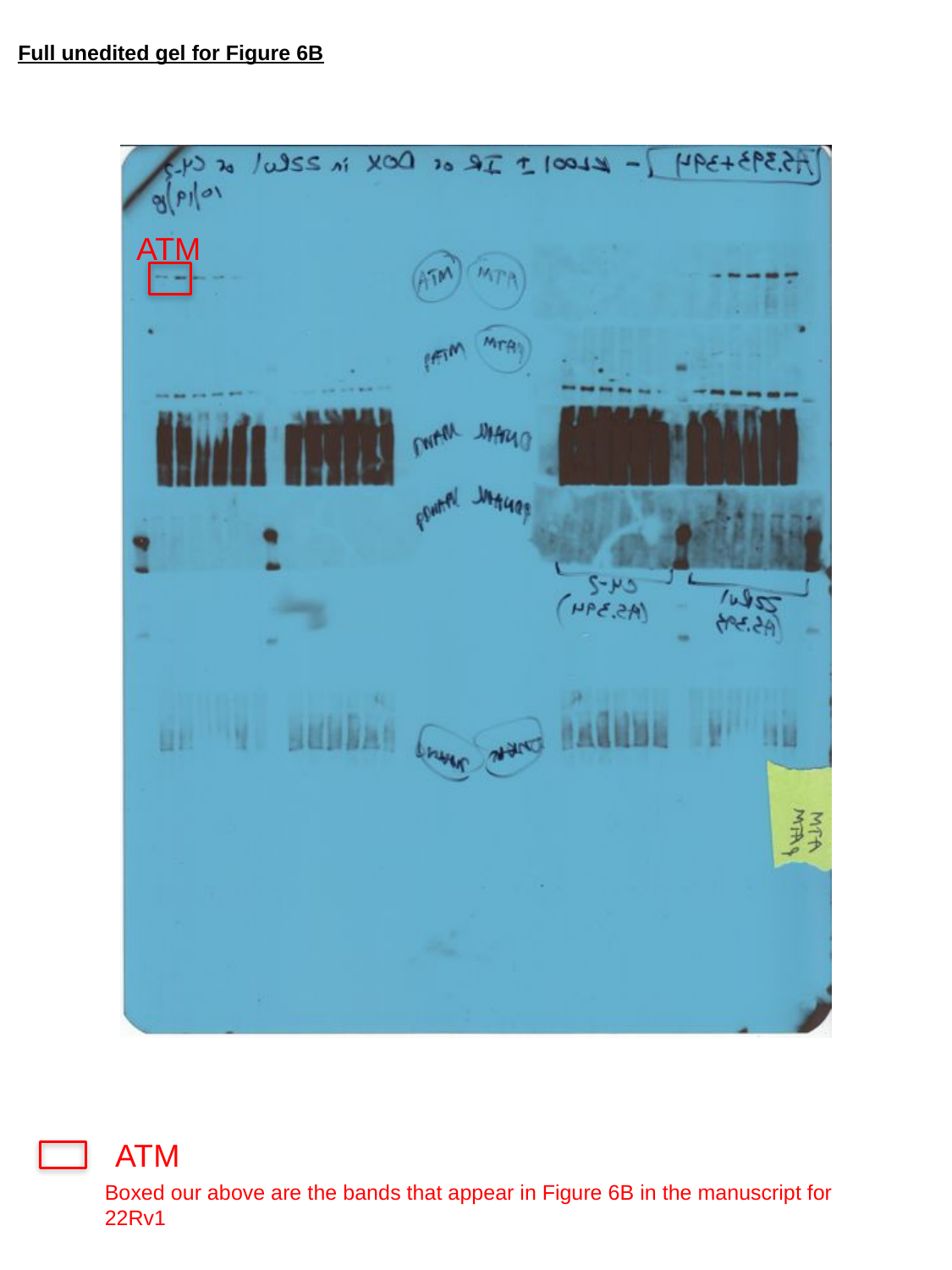

Full unedited gel for Figure 6B
ATM
ATM
Boxed our above are the bands that appear in Figure 6B in the manuscript for 22Rv1

## Slide 47
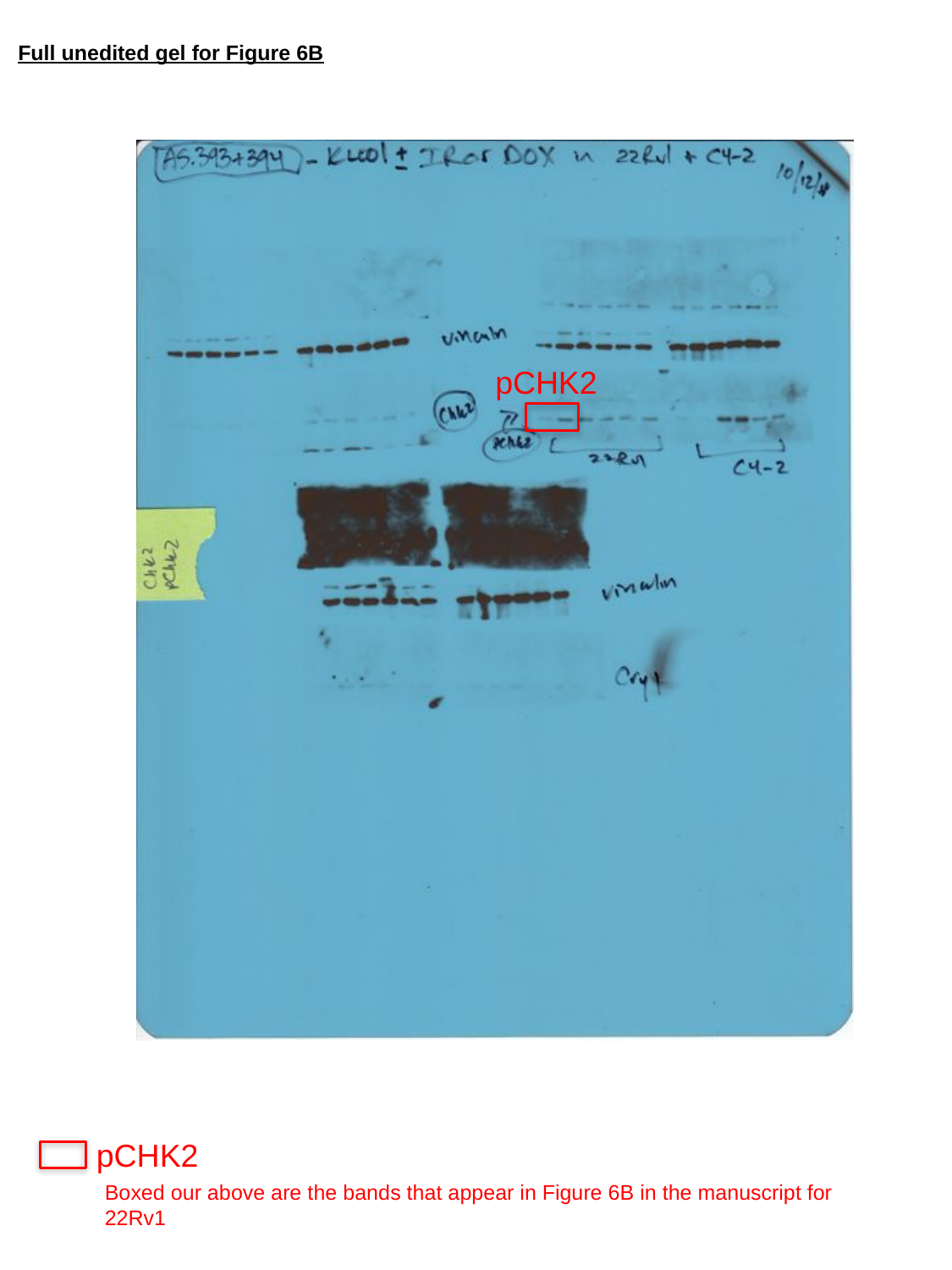

Full unedited gel for Figure 6B
pCHK2
pCHK2
Boxed our above are the bands that appear in Figure 6B in the manuscript for 22Rv1

## Slide 48
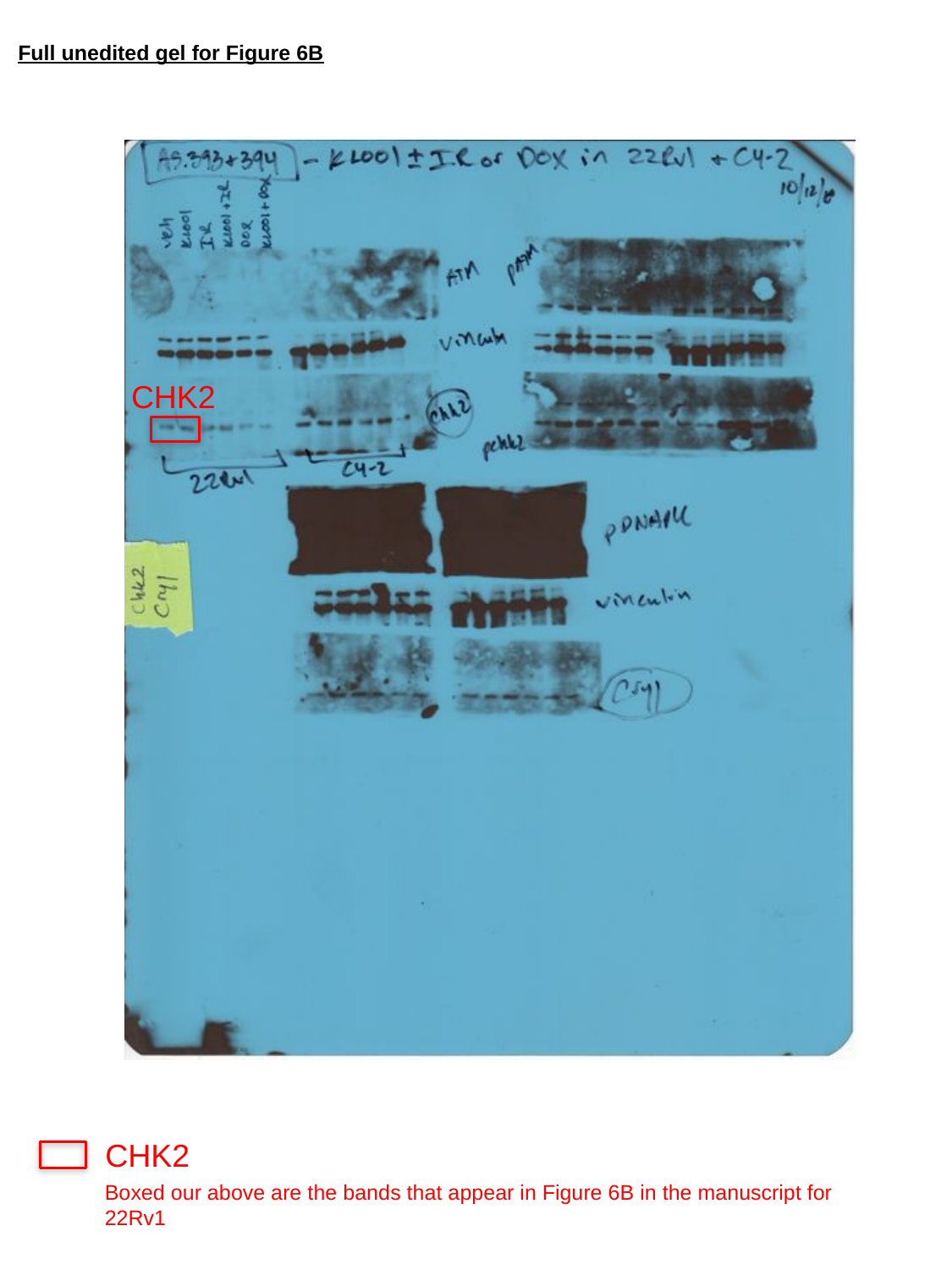

Full unedited gel for Figure 6B
CHK2
CHK2
Boxed our above are the bands that appear in Figure 6B in the manuscript for 22Rv1

## Slide 49
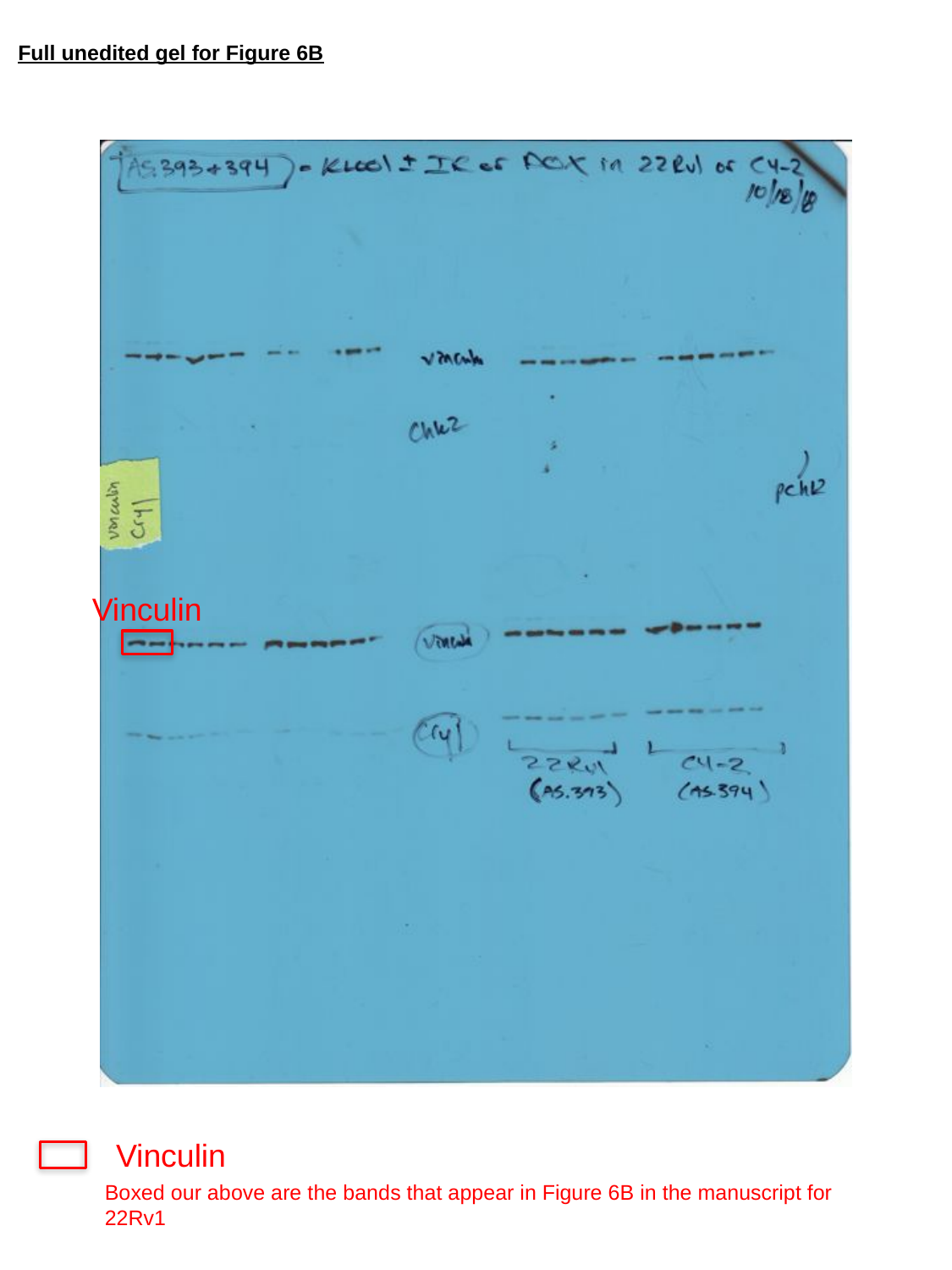

Full unedited gel for Figure 6B
Vinculin
Vinculin
Boxed our above are the bands that appear in Figure 6B in the manuscript for 22Rv1

## Slide 50
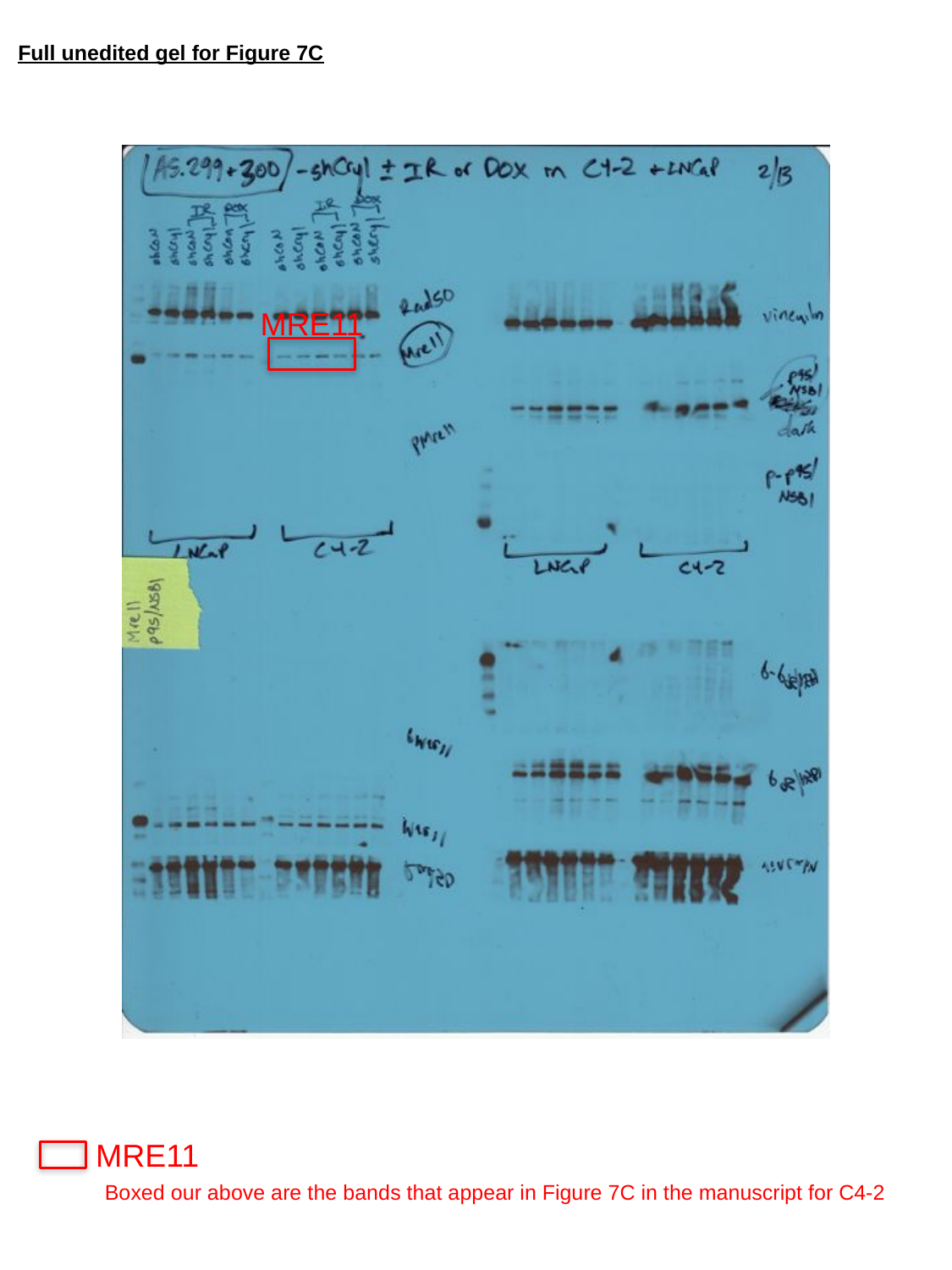

Full unedited gel for Figure 7C
MRE11
MRE11
Boxed our above are the bands that appear in Figure 7C in the manuscript for C4-2

## Slide 51
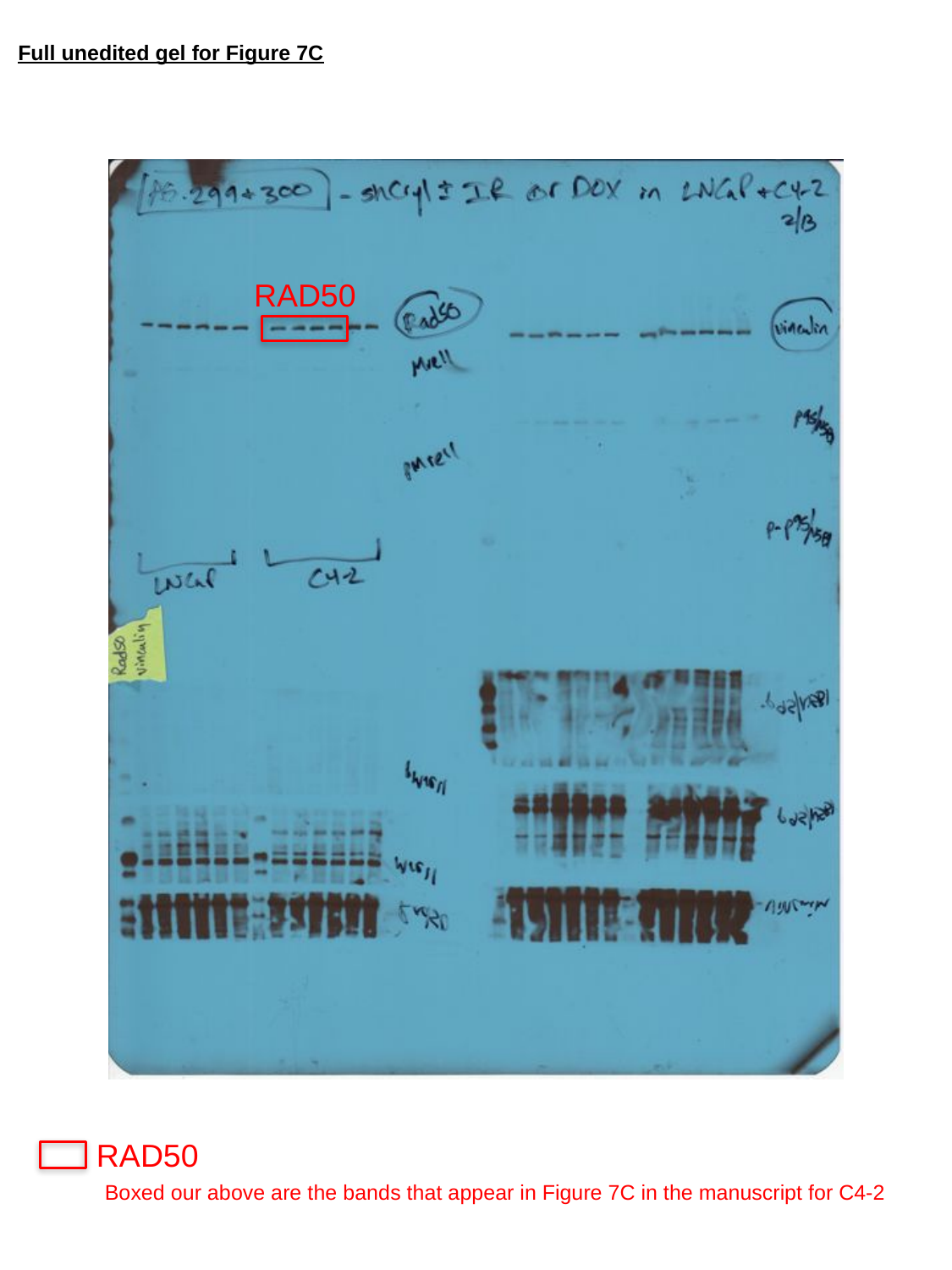

Full unedited gel for Figure 7C
RAD50
RAD50
Boxed our above are the bands that appear in Figure 7C in the manuscript for C4-2

## Slide 52
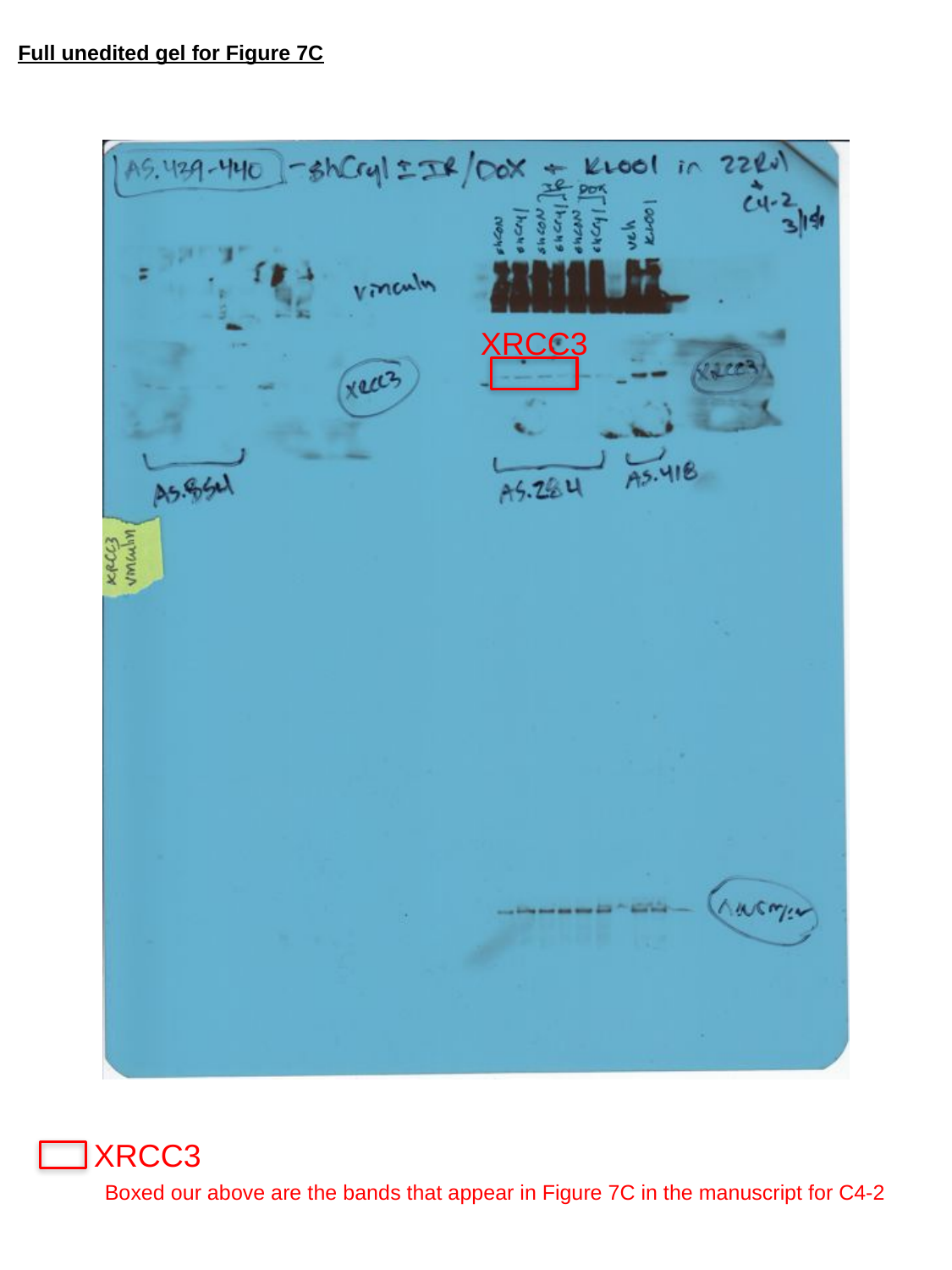

Full unedited gel for Figure 7C
XRCC3
XRCC3
Boxed our above are the bands that appear in Figure 7C in the manuscript for C4-2

## Slide 53
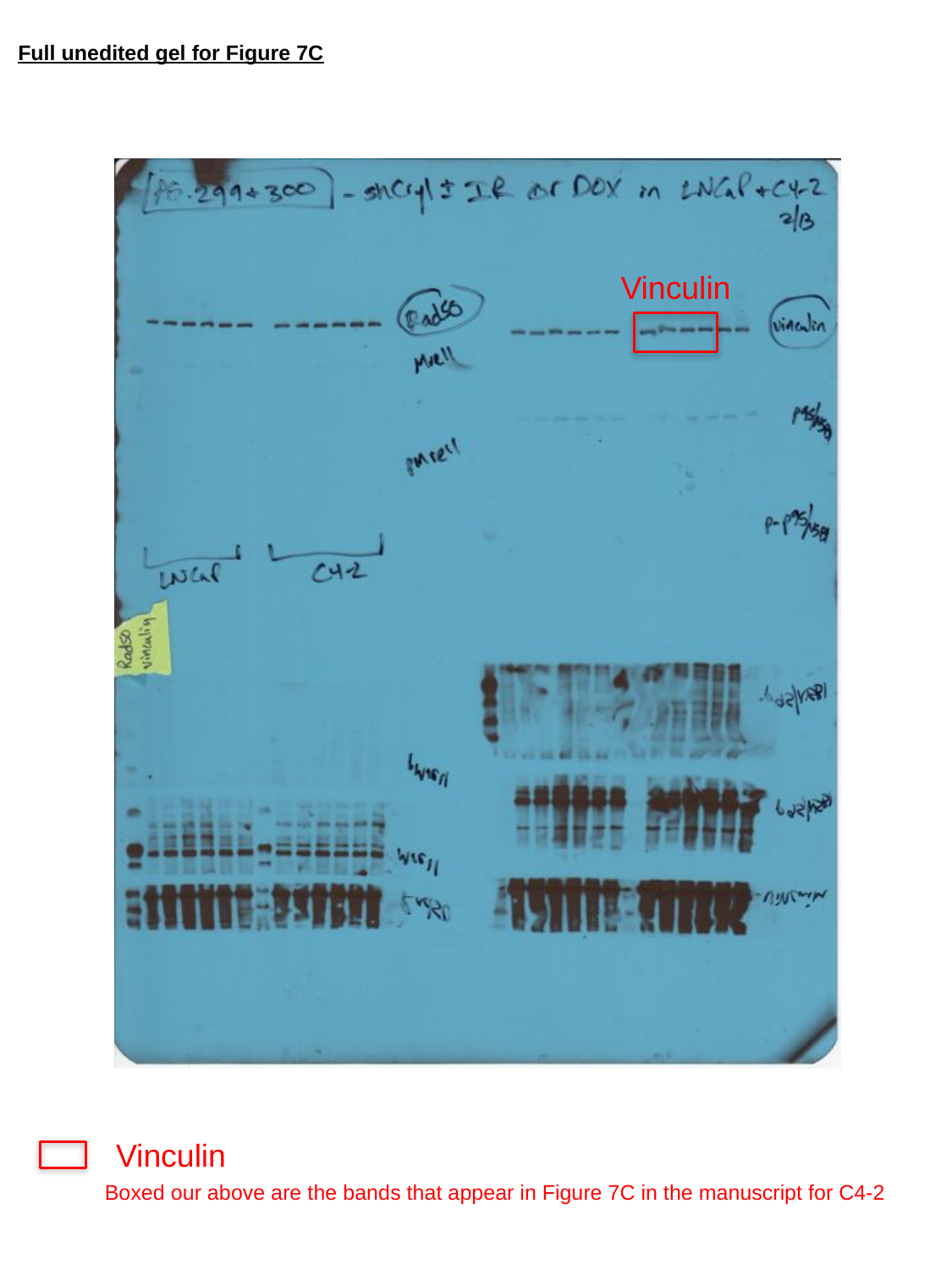

Full unedited gel for Figure 7C
Vinculin
Vinculin
Boxed our above are the bands that appear in Figure 7C in the manuscript for C4-2

## Slide 54
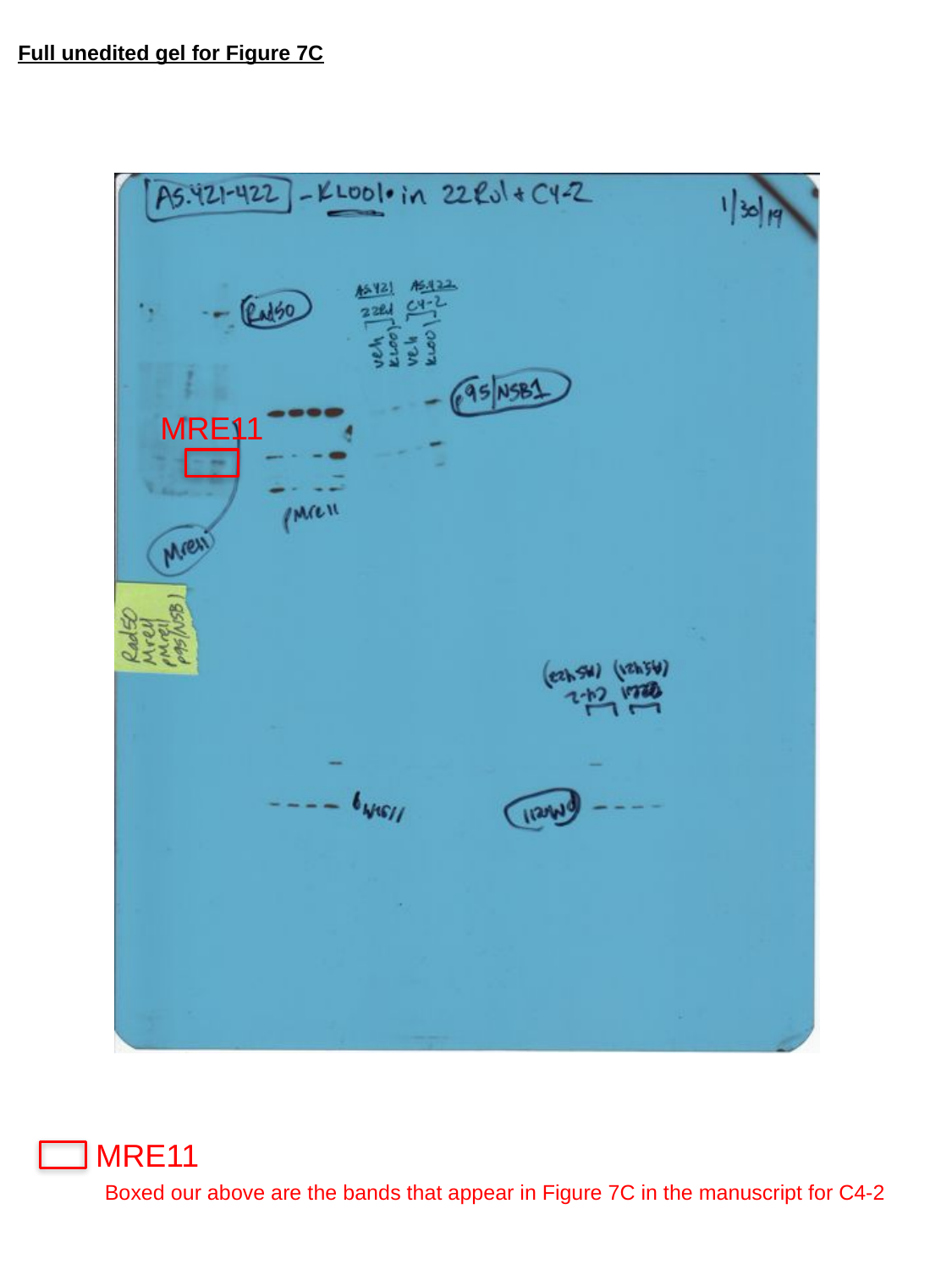

Full unedited gel for Figure 7C
MRE11
MRE11
Boxed our above are the bands that appear in Figure 7C in the manuscript for C4-2

## Slide 55
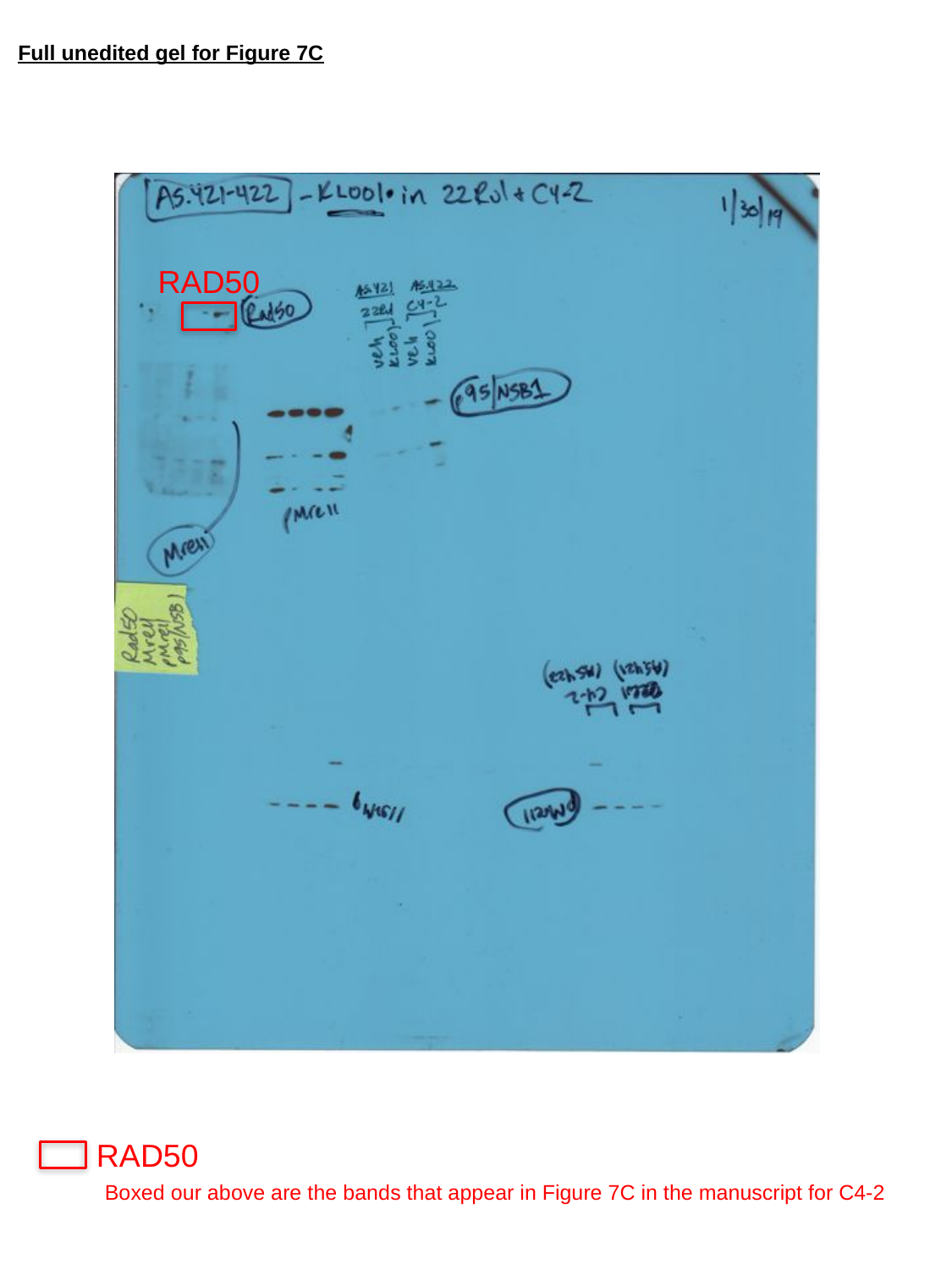

Full unedited gel for Figure 7C
RAD50
RAD50
Boxed our above are the bands that appear in Figure 7C in the manuscript for C4-2

## Slide 56
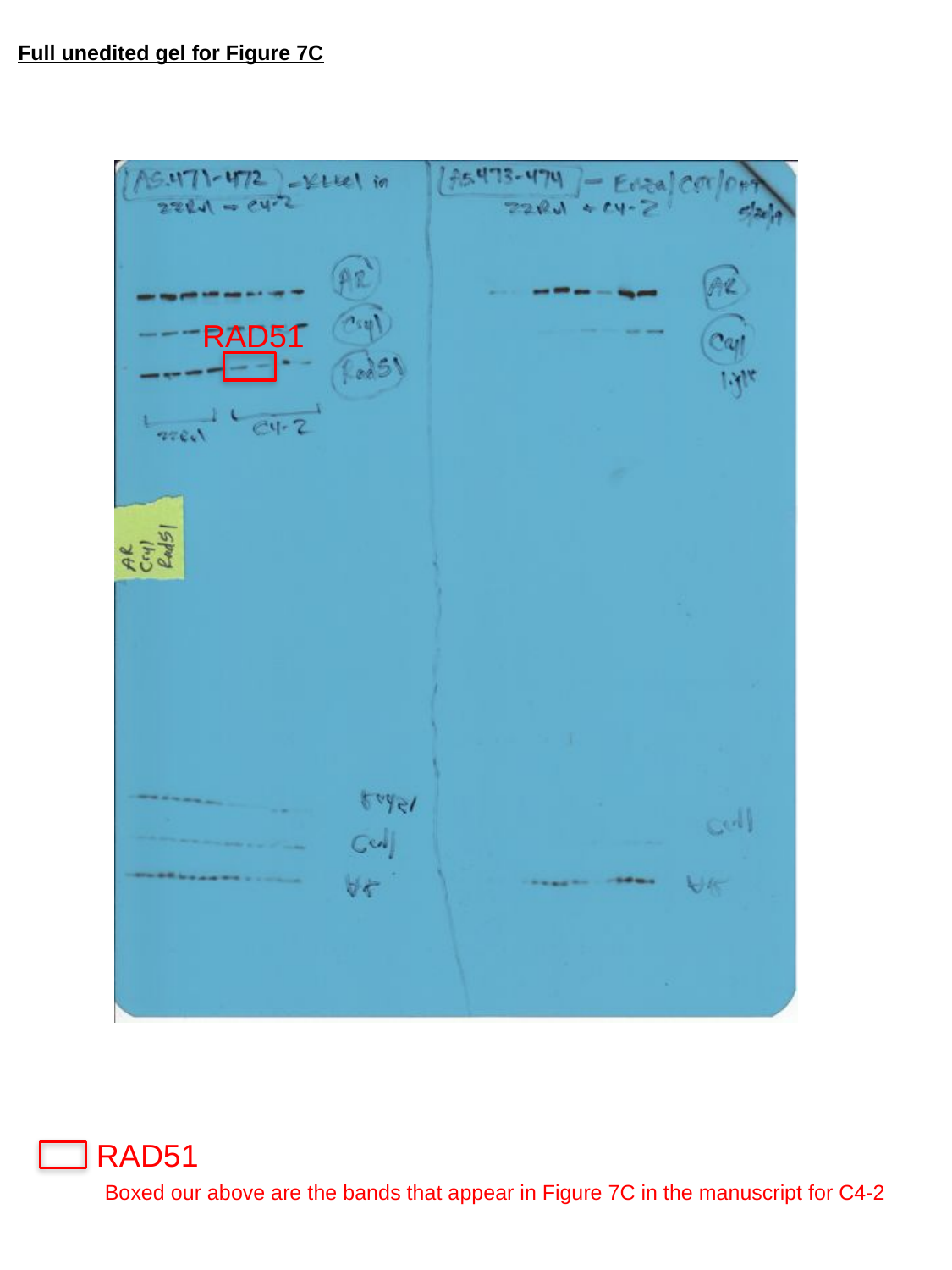

Full unedited gel for Figure 7C
RAD51
RAD51
Boxed our above are the bands that appear in Figure 7C in the manuscript for C4-2

## Slide 57
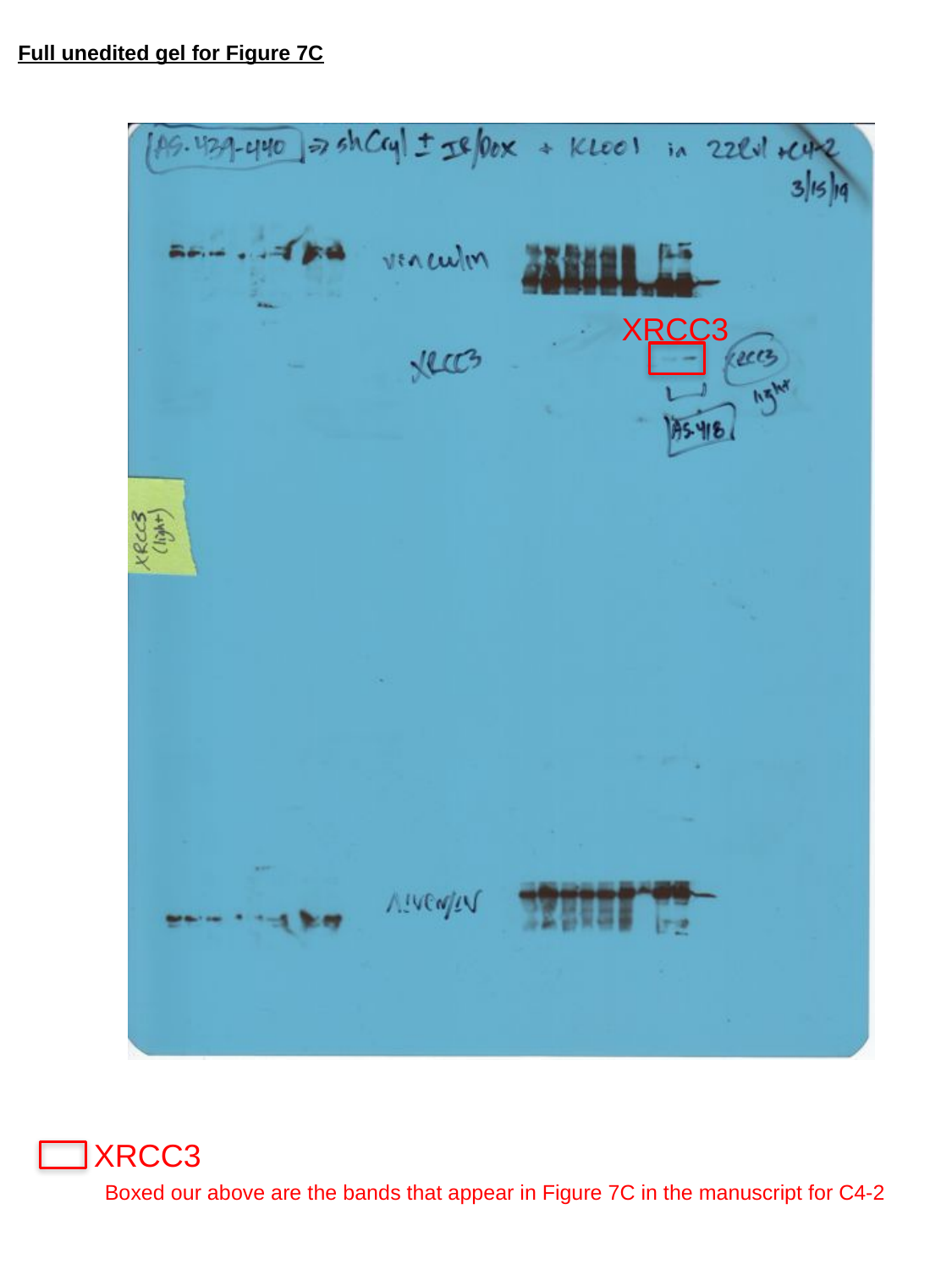

Full unedited gel for Figure 7C
XRCC3
XRCC3
Boxed our above are the bands that appear in Figure 7C in the manuscript for C4-2

## Slide 58
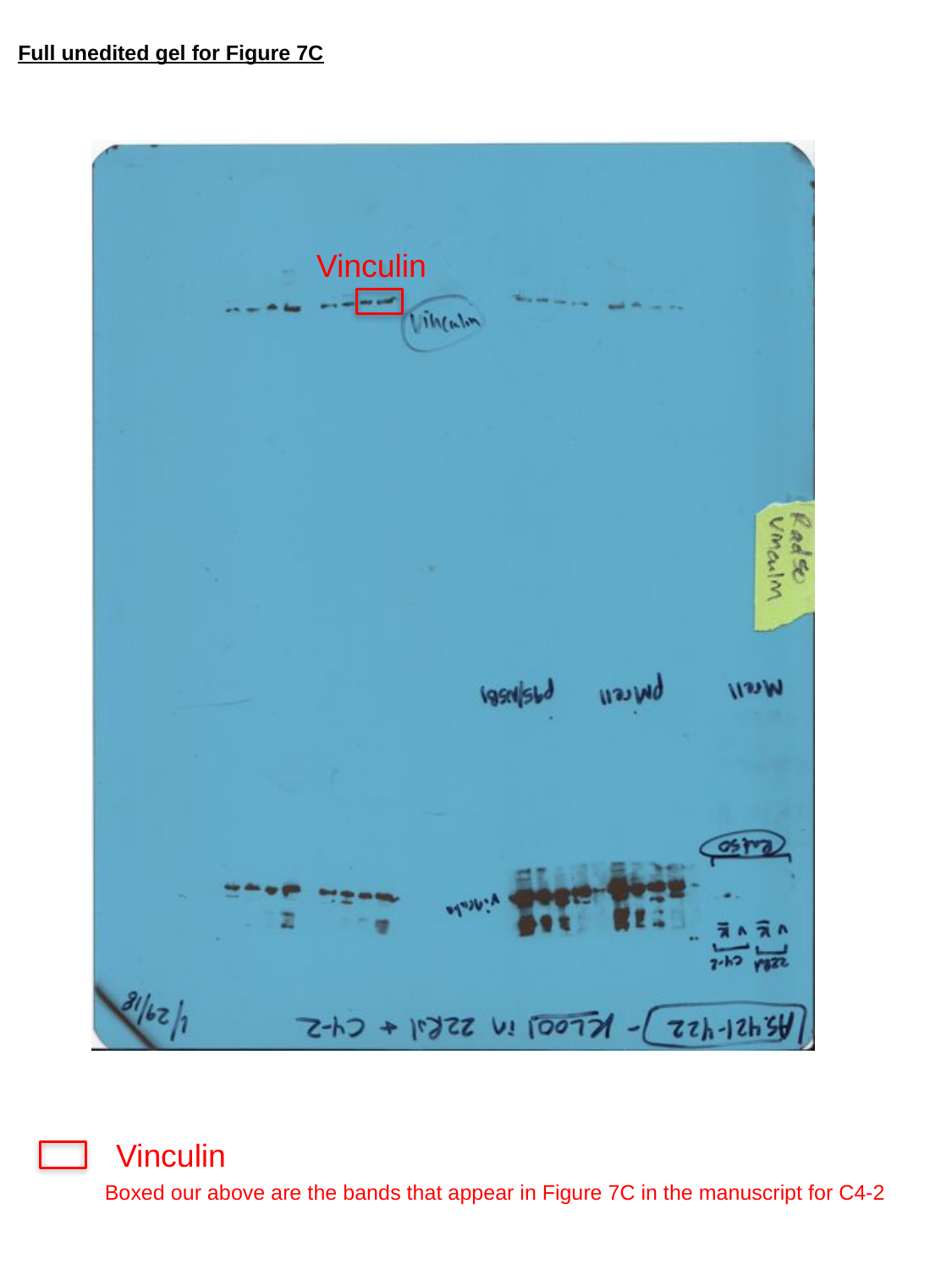

Full unedited gel for Figure 7C
Vinculin
Vinculin
Boxed our above are the bands that appear in Figure 7C in the manuscript for C4-2
